# Lipid exposure prediction enhances the inference of rotational angles of transmembrane helices

**DOI:** 10.1186/1471-2105-14-304

**Published:** 2013-10-11

**Authors:** Jhih-Siang Lai, Cheng-Wei Cheng, Allan Lo, Ting-Yi Sung, Wen-Lian Hsu

**Affiliations:** 1 Institute of Information Science, Academia Sinica, Taipei, Taiwan; 2 Verinata Health, an Illumina Company, Redwood City, CA 94063, USA

## Abstract

**Background:**

Since membrane protein structures are challenging to crystallize, computational approaches are essential for elucidating the sequence-to-structure relationships. Structural modeling of membrane proteins requires a multidimensional approach, and one critical geometric parameter is the rotational angle of transmembrane helices. Rotational angles of transmembrane helices are characterized by their folded structures and could be inferred by the hydrophobic moment; however, the folding mechanism of membrane proteins is not yet fully understood. The rotational angle of a transmembrane helix is related to the exposed surface of a transmembrane helix, since lipid exposure gives the degree of accessibility of each residue in lipid environment. To the best of our knowledge, there have been few advances in investigating whether an environment descriptor of lipid exposure could infer a geometric parameter of rotational angle.

**Results:**

Here, we present an analysis of the relationship between rotational angles and lipid exposure and a support-vector-machine method, called TMexpo, for predicting both structural features from sequences. First, we observed from the development set of 89 protein chains that the lipid exposure, i.e., the relative accessible surface area (rASA) of residues in the lipid environment, generated from high-resolution protein structures could infer the rotational angles with a mean absolute angular error (MAAE) of 46.32˚. More importantly, the predicted rASA from TMexpo achieved an MAAE of 51.05˚, which is better than 71.47˚ obtained by the best of the compared hydrophobicity scales. Lastly, TMexpo outperformed the compared methods in rASA prediction on the independent test set of 21 protein chains and achieved an overall Matthew’s correlation coefficient, accuracy, sensitivity, specificity, and precision of 0.51, 75.26%, 81.30%, 69.15%, and 72.73%, respectively. TMexpo is publicly available at http://bio-cluster.iis.sinica.edu.tw/TMexpo.

**Conclusions:**

TMexpo can better predict rASA and rotational angles than the compared methods. When rotational angles can be accurately predicted, free modeling of transmembrane protein structures in turn may benefit from a reduced complexity in ensembles with a significantly less number of packing arrangements. Furthermore, sequence-based prediction of both rotational angle and lipid exposure can provide essential information when high-resolution structures are unavailable and contribute to experimental design to elucidate transmembrane protein functions.

## Background

Integral membrane proteins participate in diverse cellular functions such as signal transductions, bioenergetics, ion transport, cell adhesion, and cell-cell recognition. It has also been estimated that about 20-30% of a typical genome encode for proteins with a transmembrane (TM) domain [1,2]. Despite their biological importance and abundance, the mechanism by which TM proteins fold into native structures remains poorly understood due to a limited number of solved structures, accounting for less than 1% of all deposited structures in the Protein Data Bank (PDB) [3]. Therefore, computational methods play an important role in deciphering the sequence-to-structure relationships and advancing our knowledge in this particular class of proteins.

Though recently solved structures of several amino-acid transporters, e.g., eukaryotic CLC Transporter (coded as 3ORG [4] in PDB) and potassium ion transporter (coded as 3PJZ [5] in PDB) revealed the existence of short helices in the reentrant region [6], the canonical topologies of TM proteins can be viewed as pairs of interacting transmembrane helices (TMHs), connecting loops and extramembraneous domains. In particular, the interaction between TMHs is an important determinant of folding and stability by the proposed two-stage model [7,8]. Such an interaction is mediated by structural contacts at the helical interfaces with the protein itself, the ligands, as well as the lipid environment. From the perspective of structural modeling, the rotational angle of a TMH is a strong determinant of its interacting faces with the rest of the protein structure and the lipids. At the stage of conformation space sampling, we could filter out decoys that severely deviate from the predicted rotational angles. To elucidate rotational angles, Eisenberg et al. [9,10] showed that hydrophobic scales can be used to estimate the hydrophobic moment direction to approximate the lipid-facing direction and proposed equations to calculate the rotational angles of TMHs based on such property. Later, several hydrophobicity scales or propensities have been proposed [11-15] to predict exposed residues or faces. To the best of our knowledge, there have been few advances to use lipid exposure, specifically the relative accessible surface area (rASA) in the lipid environment, to predict rotational angles. Henceforth, we use rASA for convenience to represent rASA in the lipid environment since in this paper we focus on the residues in such environment.

To determine the rotational angle of each TMH in the tertiary structure of a TM protein requires the information of lipid-facing direction, which has been defined differently in the literature. Pilpel et al. [12] described the lipid-facing direction as the vector opposite to the bisector of the acute angle formed by the two lines from the geometric center of the target TMH pointing to the geometric centers of the two nearest TMHs in the whole molecule. Stevens and Arkin [16] defined lipid-facing direction of a TMH as the vector connecting the geometric centers of the target helix and the whole molecule. The molecule could be a single chain or the complete protein. Dastmalchi et al. [15] accepted both the above definitions for lipid-facing direction.

Lipid exposure of each TMH has been shown useful in distinguishing between surfaces and interior interfaces, identifying potential functional residues buried in the protein core and exposed residues for protein-protein interactions [14,17], and therefore facilitates the prediction of helix-packing conformations [17,18]. In this work, we propose to use predicted rASA to estimate lipid-facing direction and then determine rotational angles of TMHs.

In order to train machine learning models by observed rASA from solved structures, we calculated the accessible surface area (ASA) by rolling a spherical probe along the van der Waal’s (VDW) surface of a protein molecule [19]. The observed rASA for each residue is defined as dividing ASA by its reference value in an extended Gly-X-Gly tripeptide conformation. Several methods have been proposed to predict ASA instead of rASA in the lipid environment. For example, ASAP [20] uses evolutionary profiles to predict solvent accessibility by the support vector regression (SVR) and reports a Pearson correlation coefficient (PCC) of 0.62 for ASA prediction. The MPRAP [21] uses the support vector machine (SVM) to predict ASA of complete TM proteins by evolutionary profiles and residue distance from membrane center [22]. A number of methods have been proposed to predict the burial or exposed status of each TM residue, where the status is determined by rASA whether below a predefined threshold. Several methods predict the status of TM residues directly from sequences without predicting their ASA; most of them rely on sequence conservation or knowledge-based propensities, including kPROT [12], ProperTM [11], TMLIP [13], MO [14] and TMX [23]. RHYTHM [24] predicts the burial status of TM residues by matrix-based helix-helix contact prediction and sequence conservation, but this method requires prior knowledge of TMHs such as membrane coils or transporter/channels.

In this paper, we present TMexpo, a method to predict rotational angles of TMHs. For each TM residue, TMexpo first predicts rASA by SVR and predicts the burial or exposed status by SVM; both models use evolutionary profiles, sequence conservation, helix insertion energy and biochemical properties as features. Next, TMexpo determines rotational angles of TMHs based on the predicted rASA. In rotational angle prediction, TMexpo outperformed predictors using hydrophobicity scales and propensities by at least 19.2˚ in terms of mean absolute angular error (MAAE) on the independent test set of 21 protein chains. Notably, the prediction results showed that rotational angles of TMHs could be better inferred by predicted rASA than by existing scales. We expect the rotational angle prediction could benefit the structure prediction, especially for free modeling of transmembrane protein structures due to its difficulty and the necessities of reducing the number of packing arrangements.

## Results and discussion

### Observed rASA can better infer the lipid-facing direction than hydrophobicity scales and lipid-facing propensities

The packing mechanism in which TM protein assembles in the lipids is not fully understood. One significant advance in this area is seen in the mechanism of the Sec translocon which demonstrates how TM proteins enter the membrane [25-27]. A commonly accepted model of TM protein folding is the two-stage model [7]. Later, White and colleagues extended the two-stage model by including a four-step thermodynamic cycle of folding energy description [28]. Several scales and propensities were developed to understand the lipid-facing direction of TMHs based on sequence analysis or knowledge-based information; however, using these scales to predict the rotational angle of TM proteins often results in low prediction accuracy. Therefore, we investigated whether an environment descriptor such as lipid exposure could better infer a geometric parameter such as the rotational angles of TMHs.

To gain insights into the relationship between lipid exposure and rotational angles, we analyzed a dataset of 110 multi-spanning protein chains with high-resolution structures, which were divided into a development set of 89 protein chains and an independent test set of 21 protein chains (described in Methods). We first used the NACCESS program [19,29] to determine the observed rASA of each TM residue. We then calculated the rotational angle of each TMH by the rASA moment (Equations 6, 7, 8, 9 described in Methods) derived from the observed rASA and compared it with the rotational angle calculated from existing hydrophobicity scales or propensities. The comparison results on the dataset in terms of MAAE are shown in Table 1. It can be observed from Table 1 that the rotational angle calculated from the observed rASA achieves the best MAAE, outperforming the propensity-based methods by at least 25.15˚ on the development set and 21.96˚ on the independent test set. It demonstrates that the observed rASA could better infer the rotational angles of TMHs.

**Table 1:** The MAAE of rotational angles determined by various approaches

**Methods**	**MAAE in development set (554 TMHs)**	**MAAE in independent test set (188 TMHs)**
NACCESS (observed rASA)	**46.32°**	**45.55°**
ES	72.85°	68.35°
kPROT	74.26°	68.84°
ProperTM	76.59°	70.69°
TMLIP1H	81.02°	76.35°
TMLIP2H	81.33°	76.90°
TMLIP1C	73.24°	67.51°
TMLIP2C	74.23°	68.27°
MO	71.47°	69.43°
TMexpo (predicted rASA)	**51.05° (LOOCV)**	**48.31°**

Comparison of mean absolute angular errors (MAAE) of rotational angles obtained by different methods on the development set and the independent test.

The success of determining the rotational angle via the observed rASA calculated by NACCESS may be due to the following two reasons. First, the description of observed rASA is derived from known protein structures, but the descriptions of hydrophobicity and lipid-facing propensities are derived from the sequence. Therefore, hydrophobicity and lipid-facing propensities alone are insufficient for accurate inference of helical packing, thereby rendering worse rotational angle estimation. Second, researchers had a simplified view of membrane proteins being “inside-out” proteins, which have interior polar core and exterior apolar surface [30,31]. However, this paradigm was challenged since biased distribution of hydrophobic residues could not be detected in every membrane protein [32]. As more solved structures become available, statistical analyses on these structures also support the above finding [33-36]. The canonical view of the “inside-out” property of membrane proteins based on hydrophobicity is challenged. On the contrary, observed rASA is a structural environment descriptor of helical packing and better infer the rotational angle.

### Relative accessible surface area predicted by TMexpo can also better infer the lipid-facing direction than hydrophobicity scales and lipid-facing propensities

To evaluate the capability of our proposed method for TM proteins with unknown structures, we used rASA predicted by TMexpo to determine the rotational angles of TMHs. Then we evaluated how predicted rASA could infer the rotational angle in terms of MAAE by the following two experiments. First, we used the development set of 89 chains to develop the TMexpo prediction model and tested on the independent test set of 21 chains. Second, we performed leave-one-out cross validation (LOOCV) on the development set. The results of both experiments are shown in Table 1. Both rASA prediction results of the development set and the independent test set are provided in the Additional file 1: Dataset S1. Particularly, detailed prediction results of 188 TMHs in the independent test set are reported in the Additional file 2: Table S1. Notably, 44 of 188 TMHs had the angular error less than 15˚ and their rotational angles were predicted precisely by TMexpo.

Observed from the first experiment, TMexpo-predicted rASA is shown to be comparable to the observed rASA derived from NACESS for inferring the rational angles; the predicted rASA achieved an MAAE of 48.31˚, slightly worse than the MAAE of 45.55˚ achieved by the observed rASA. Nevertheless, TMexpo-predicted rASA achieved much better MAAE than the other predictors using different hydrophobicity scales and propensities, including Eisenberg et al.’s consensus hydrophobicity scale (ES) [10], kPROT [12], ProperTM [11], TMLIP [13], and MO [14] by at least 19.2˚. The second experiment reported consistent results with the first experiment. Specifically, performing LOOCV on the development set resulted in the MAAE of 51.05˚, slightly worse than that inferred by the observed rASA, but better than the compared predictors by at least 20.42˚. Our results show that without known structures, TMexpo can effectively infer the rotational angle within a close margin to that inferred by the observed rASA and improve the prediction compared to hydrophobicity scales or lipid-facing propensities.

### Comparison of relative accessible surface area prediction methods

The proposed method TMexpo is capable of predicting not only the rotational angles of TMHs but also rASA of TM residues. We evaluated real-number rASA and binary classification of burial and exposed status predicted by TMexpo on the independent test set of 21 protein chains. At first, we demonstrated the performances of TMexpo in which TM residues participate in interchain contacts were excluded from the independent test set. The results are shown in Table 2, where exposed residues defined by observed rASA ≥5% are regarded as positive cases for evaluating the classification performance. TMexpo achieved an overall PCC of 0.66, mean absolute error (MAE) of 0.12 and root mean squared error (RMSE) of 0.16 in predicting rASA. With respect to the binary classification, TMexpo achieved an overall MCC, accuracy, sensitivity, specificity, and precision of 0.51, 75.26%, 81.30%, 69.15%, and 72.73%, respectively.

**Table 2:** Comparison of different methods for classifying exposed/buried residues on the independent test set without interface TM residues

**Measurements**	**TMexpo**	**MPRAP**	**TMX**	**Rhythm (membrane-coil)**	**Rhythm (channel)**
MCC	**0.51**	0.35	0.44	0.32	0.29
Sensitivity	**81.30%**	64.24%	75.60%	73.53%	67.25%
Specificity	69.15%	**70.31%**	67.99%	58.03%	61.80%
Precision	**72.73%**	68.48%	68.69%	65.02%	65.17%
Accuracy	**75.26%**	67.27%	71.66%	66.01%	64.61%

The boldface indicates the best performance among various methods.

Next, we compared TMexpo’s performance of predicting rASA with existing methods, including TMX [23], RHYTHM [24] and MPRAP [21]. One distinction among these methods is that RHYTHM requires prior knowledge of protein types as membrane-coil or channel for prediction. For comparison, we retrieved the prediction results for TMX, RHYTHM and MPRAP from their web servers by using their default parameters. Table 2 shows that TMexpo outperforms the compared methods across most of the measures except a slightly lower specificity compared to MPRAP by 1.16%. The specificity of TMexpo is lower than its sensitivity by 12.15%, and most of the predictors except MPRAP have the same trend as TMexpo’s results. This observation implies that the detection of buried residues may be more difficult than that of exposed residues in the TM domains. To further gain insights into this issue, we extracted buried residues in our dataset of 110 chains from the helix-packing database TMPad [37], and found that over 77% residues have at least one interhelical contact. This suggests that prediction of buried residues could very likely be improved by detecting contacts of interhelical interactions; however, TMexpo and most of the compared predictors retrieve features by local information of subsequences only. Interestingly, interhelical contacts may be conserved in sequences and discovered from evolutionary information such as PSSM profiles [38]. We consider that evolutionary information is an effective feature for capturing interhelical interactions that contributes to rASA/burial status prediction; however, interhelical interaction prediction is still a challenging problem.

Moreover, since we excluded TM residues that participate in interchain contacts, i.e., interface TM residues, in the training of TMexpo (as described in Model Development of the subsection entitled “An SVM-based predictor for lipid exposure of TM helices” in Methods), we also compared the methods for classifying burial status of each interface TM residue in the independent test set. After removing TM residues with missing atoms, there are 392 interface TM residues out of the remained entire 3,553 TM residues in the independent test set of 21 protein chains. Since observed rASA derived from subunit structure and complete structure, respectively, can be different for interface TM residues, we used both the observed rASA to determine the “true” status of each residue. We reported the performance of each method based on the “true” observed rASA for both the 392 interface TM residues and the 3,553 entire TM residues in Table 3 and Table 4, respectively. The tables demonstrate that most rASA predictors, except MPRAP, have lower specificity than sensitivity. For the 392 interface TM residues, most predictors have better MCC calculated from complete structure than that calculated from subunit structure. Furthermore, Table 4 shows that TMexpo achieved slightly better MCC, sensitivity, and accuracy on the 3,553 entire TM residues.

**Table 3:** Comparison of different methods for classifying exposed/buried residues on 392 interface TM residues of the independent test set by rASA derived from both subunit structure and complete structure (in parentheses)

**Measurements**	**TMexpo**	**MPRAP**	**TMX**	**Rhythm (membrane-coil)**	**Rhythm (channel)**
MCC	0.14 (0.22)	0.12 (0.25)	0.13 (0.17)	0.08 (−0.06)	0.19 (0.28)
Sensitivity	73.11% (81.25%)	56.29% (67.23%)	66.17% (72.78%)	67.72% (63.22%)	66.10% (77.97%)
Specificity	48.57% (38.50%)	64.71% (57.59%)	55.88% (43.98%)	45.00% (30.77%)	64.29% (49.32%)
Precision	93.55% (55.91%)	94.00% (59.50%)	93.70% (55.04%)	90.68% (46.61%)	93.98% (55.42%)
Accuracy	70.92% (59.44%)	57.07% (62.23%)	65.23% (57.95%)	65.17% (46.63%)	65.91% (62.12%)

**Table 4:** Comparison of different methods for classifying exposed/buried residues on 3,553 entire TM residues of the independent test set by rASA derived from both subunit structure and complete structure (in parentheses)

**Measurements**	**TMexpo**	**MPRAP**	**TMX**	**Rhythm (membrane-coil)**	**Rhythm (channel)**
MCC	0.49 (0.48)	0.33 (0.34)	0.41 (0.41)	0.30 (0.27)	0.29 (0.29)
Sensitivity	79.79% (81.29%)	62.85% (64.55%)	73.70% (73.70%)	72.43% (72.36%)	67.07% (68.18%)
Specificity	68.70% (65.69%)	70.19% (68.93%)	67.71% (67.71%)	57.64% (54.64%)	61.86% (60.42%)
Precision	75.56% (70.45%)	71.53% (67.40%)	72.18% (72.18%)	68.43% (62.57%)	68.45% (64.06%)
Accuracy	74.78% (73.51%)	66.20% (66.74%)	70.90% (70.90%)	65.91% (63.71%)	64.74% (64.37%)

### Rotational angle can help in determining helical packing in transmembrane proteins

Harrington and Ben-Tal [39] characterized five structural features of interhelical interactions, namely, aromatic interactions, hydrogen bonds, salt bridges, and two interactions from packing motifs, that are useful for helical packing. They proposed an algorithm to pack the TMHs of TM proteins, as follows: First, the algorithm ordered the TMHs by the sequence from the N-terminus, and then iteratively grouped sequential TMHs by a scoring function based on the five types of interactions. They demonstrated helical packing on 15 diverse proteins, and the average RMSD of C_α_ in the native structure of the 15 reconstructed TM proteins ranged from 0.51 Å to 1.35 Å. In this subsection, we reexamined these proteins to study the rotational angle of TMHs and its relationship to helical packing. Since the protein 1AFO discussed in their work contains only one TMH, we excluded this protein in our analysis.

Table 5 shows angular error of the 14 proteins. The MAAE of all 73 TMHs of the 14 proteins is 41.04˚, and 60% of all TMHs were predicted with MAAE ≤43˚. The worst MAAE (113.71˚) was found in protein 2OAR, and the best case is 2UUH (10.47˚). Out of all 73 TMHs, 13 TMHs were predicted with angular errors less than 10˚. With further investigation, we observed some common features from TMPad. First, most of TMHs are linear or curved, and three TMHs are slightly kinked in their structures. Second, we observed strong interhelical interactions constrained in a single helical interface in most of these cases. For example, in the acid-sensing ion channel 2QTS:A [40], its TMH1 (curved) shares 7 contact pairs with TMH2. The second example is an avian mitochondrial complex II (2H88:C) [41], where its TMH3 (curved) shares 13 contact pairs with TMH2. Lastly, in the structure of aquaporin-0 (2B6O:A) [42], its TMH6 (linear) associates with TMH4 via a dense cluster of 16 contacts. Based on the above findings, regular TMHs with strong and constrained interhelical interactions in a single helical interface reveal periodicity in rASA of their residues, thus making rotational angle prediction highly accurate.

**Table 5:** Rotational angle prediction on the protein chains from Harrington and Ben-Tal’s work

**PDB:Chain (MAAE)**	**TM helix sequence**	**Observed angle**	**Predicted angle**	**Angular error**	$\left|\hat{M}\right|$	**PDB:Chain (MAAE)**	**TM helix sequence**	**Observed angle**	**Predicted angle**	**Angular error**	$\left|\hat{M}\right|$
**1BL8:A**	HWRAAGAATVLLVIVLLAGSYLAVLA	199.55°	203.38°	**3.83°**	1.46	**2H88:D**	VSALLLGLLPAAYLYPG	229.60°	234.10°	**4.50°**	1.06
(30.86)	WGRCVAVVVMVAGITSFGLVTAALAT	240.88°	298.78°	57.90°	1.87	(27.22)	AVDYSLAAALTLHGHWGL	8.24°	354.67°	13.57°	0.95
**1C3W:A**	IWLALGTALMGLGTLYFLVKGMG	318.98°	355.18°	36.19°	3.38		GLYVLSAITFTGLCYFNYYDV	334.89°	271.30°	63.59°	1.49
(42.28)	KFYAITTLVPAIAFTMYLSMLL	262.69°	313.32°	50.63°	2.74	**2OAR:A**	VAVVIGTAFTALVTKFTDSIITPLINRIG	319.36°	203.39°	115.96°	0.37
	WARYADWLFTTPLLLLDLALL	118.84°	44.89°	73.95°	1.60	(113.71)	TIDLNVLLSAAINFFLIAFAVYFL	105.75°	354.30°	111.46°	1.06
	GTILALVGADGIMIGTGLVGAL	357.99°	332.94°	25.05°	2.80	**2QTS:A**	VWALCFMGSLALLALVCTNRIQ	285.04°	280.66°	**4.38°**	2.32
	RFVWWAISTAAMLYILYVLFFGF	154.03°	169.74°	15.71°	2.25	(11.07)	AGLLGDIGGQMGLFIGASILTVL	41.15°	23.39°	17.76°	2.22
	FKVLRNVTVVLWSAYPVVWLIG	133.56°	164.23°	30.67°	2.70	**2RH1:A**	WVVGMGIVMSLIVLAIVFGNVLVITAIA	253.60°	259.99°	**6.39°**	1.71
	ETLLFMVLDVSAKVGFGLILLRS	214.98°	278.73°	63.75°	1.93	(35.73)	YFITSLACADLVMGLAVVPFGAAHIL	293.99°	341.90°	47.91°	1.06
**1OKC:A**	LSFLKDFLAGGVAAAISKTAVAPIER	24.46°	46.33°	21.87°	1.59		WCEFWTSIDVLCVTASIETLCVIAV	279.34°	285.10°	**5.77°**	0.68
(65.78)	NLANVIRYFPTQALNFAFKDKYKQIFL	207.77°	114.64°	93.14°	1.75		RVIILMVWIVSGLTSFLPIQMHWYR	88.76°	102.09°	13.33°	1.93
	WRYFAGNLASGGAAGATSLCFVYPLDFART	112.40°	70.30°	42.09°	1.32		FTNQAYAIASSIVSFYVPLVIMVFVYS	158.19°	84.71°	73.47°	1.88
	YQGFNVSVQGIIIYRAAYFGVYDTAKGMLP	179.94°	59.44°	120.50°	1.40		LGIIMGTFTLCWLPFFIVNIVHVIQ	106.50°	159.31°	52.82°	1.40
	HIIVSWMIAQTVTAVAGLVSYPFDTVRR	264.34°	315.20°	50.85°	0.98		IRKEVYILLNWIGYVNSGFNPLIYC	271.42°	321.81°	50.39°	1.50
	AWSNVLRGMGGAFVLVLYDEI	172.16°	105.90°	66.25°	1.87	**2UUH:A**	AAVTLLGVLLQAYF	60.17°	77.84°	17.67°	1.38
**1ORS:C**	VELGVSYAALLSVIVVVVEYTMQL	268.82°	191.51°	77.31°	0.70	(10.47)	SEYFPLFLATLWVAG	96.55°	80.38°	16.17°	0.77
(62.09)	LVRLYLVDLILVIILWADYAY	167.76°	133.29°	34.46°	1.71		AALCGLVYLFARLR	191.79°	197.96°	**6.17°**	2.39
	KKTLYEIPALVPAGLLALIE	27.58°	321.07°	66.51°	0.72		LYASARALWLLVALAAL	116.59°	118.45°	**1.86°**	2.57
	LVRLLRFLRILLIISRGSKFLSAIA	233.80°	303.87°	70.07°	0.62	**2Z73:A**	SLGIFIGICGIIGCGGNGIVIY	276.78°	317.73°	40.94°	3.01
**2B6O:A**	RAIFAEFFATLFYVFFGLGAS	308.42°	335.32°	26.90°	1.44	(35.30)	FIINLAFSDFTFSLVNGFPLMTI	206.68°	252.82°	46.14°	1.12
(33.01)	LQVALAFGLALATLVQAVGHIS	59.48°	65.56°	**6.07°**	1.17		VYGFIGGIFGFMSIMTMAMISI	303.09°	339.23°	36.14°	0.80
	LRAICYVVAQLLGAVAGAAVLYSV	354.70°	13.71°	19.01°	2.35		FIMIIFVWLWSVLWAIGPIF	99.36°	72.24°	27.11°	2.67
	GQATIVEIFLTLQFVLCIFATY	61.06°	71.11°	10.05°	1.58		NILCMFILGFFGPILIIFFCYF	270.54°	293.89°	23.35°	2.49
	GSVALAVGFSLTLGHLFGM	342.15°	109.29°	127.14°	1.21		SIVIVSQFLLSWSPYAVVAL	127.57°	154.75°	27.18°	2.12
	WVYWVGPVIGAGLGSLLYDFLL	49.77°	58.65°	**8.88°**	1.58		QLPVMFAKASAIHNPMIYSV	60.63°	106.85°	46.22°	2.01
**2BL2:A**	VLAMATATIFSGIGSAKGVG	45.15°	105.31°	60.16°	0.99	**3B9W:A**	YSINILAMLLVGFGFLMV	232.00°	229.10°	**2.90°**	0.63
(41.83)	LPGTQGLYGFVIAFLIFI	285.84°	259.80°	26.04°	1.70	(33.25)	ATTGTYLVVATGLPLYILL	193.50°	227.23°	33.73°	0.87
	LGASLPIAFTGLFSGIAQ	82.57°	87.25°	**4.68°**	1.39		IYAEFAVATGLIAMGAVL	221.13°	199.82°	21.31°	0.05
	MVETYAILGFVISFLLVL	7.10°	290.64°	76.46°	1.13		FQYALLALFIVPVYLLNE	11.39°	35.81°	24.42°	1.15
**2BS2:C**	WQSATGLFLGLFMIGHMFFVST	285.17°	308.25°	23.08°	1.79		GSIAIHAFGAYFGLGVSIA	208.92°	309.21°	100.29°	0.73
(51.33)	IVVSFLAAFVFAVFIAHAFLAMR	55.33°	17.57°	37.76°	2.89		FSMLGSMVLWLFWPSFA	284.14°	287.41°	**3.27°**	1.16
	LWWIQAMTGFAMFFLGSVHLYIMMTQP	188.56°	222.27°	33.71°	1.48		VNTLLALCGATLATYFLSAL	36.54°	3.47°	33.07°	1.57
	WMWPLYLVLLFAVELHGSVGLYRLAV	192.12°	322.45°	130.33°	1.38		VDMANAALAGGVAIGSVC	138.00°	55.95°	82.05°	0.24
	RANLKKLKTLMSAFLIVLGLLTFGAYV	185.58°	153.82°	31.76°	3.30		VGAFVIGLLGGAISVVGF	11.05°	21.65°	10.60°	1.90
**2H88:C**	HRGTGVALSLGVSLFSLAALLLP	123.28°	203.88°	80.60°	1.69		TCGVHNLHGLPGLLGGFSAIL	112.57°	156.18°	43.61°	0.92
(45.70)	LIYSAKFALVFPLSYHTWNGIR	307.08°	253.07°	54.01°	0.39		LTGIGITLALALIGGVIAGALIKLT	103.20°	92.65°	10.55°	2.58
	VVVLILTLLSSAAIASE	74.04°	71.55°	**2.50°**	2.05						

The columns are protein chain, sequences of transmembrane helices, observed rotational angles from structures, predicted rotational angles by TMexpo, angular errors, and predicted rASA moment lengths ($\left|\hat{M}\right|$).

On the other hand, since rotational angle prediction is strongly correlated with the periodicity of a helix, predicted rotational angles may not work well for packing of helices that deviate from regular periodicities of rASA, such as those severely kinked, disrupted, highly tilted, or associated with a reentrant loop. Six out of all 73 TMHs were poorly predicted with angular errors over 100.29˚. We observed these six TMHs being classified as kinked or containing partial non-helical structure in the TM domain, and therefore the moment-based prediction performed poorly.

### Rotational angles prediction based on both predicted topology and predicted relative accessible surface area

To illustrate the capability of the proposed method for rotational angle prediction based on predicted topology information, we submitted sequences of independent test set to three web servers including SVMSignal [43], TOPCONS [44] and MemBrain [45]. As a pre-processing step, we removed predicted N-terminal signal peptide sequences by SVMSignal. For all predicted TMHs, a correctly predicted TMH is defined as a one-to-one overlap with the observed TMH of PDBTM, and the minimum residue number of overlaps between predicted TMHs and observed TMHs of PDBTM is eight. Within 188 TMHs of independent test set, the recall and precision of TOPCONS is 93.09% (175/188) and 100% (188/188), respectively. The recall and precision of MemBrain is 97.87% (184/188) and 95.34% (184/193), respectively. For fair comparison in rotational angles, we only discuss 175 TMHs that have been predicted well by both of the two topology predictors. Prediction results from SVMSignal, TOPCONS and MemBrain are available in the Additional file 3: Dataset S2.

To obtain observed rotational angles corresponding to each predicted TMH, we removed atoms which were predicted outside the membrane by the topology prediction for each protein chain, and then we followed the definition in this work to calculate the rotational angles. Specifically, we did not directly assign rotational angles calculated by TMH of PDBTM to predicted TMHs, but we recalculated rotational angles based on atoms of protein structure selected by predicted topology within the TM region. There are two reasons to do that. First, since the sequence of a predicted TMH is not identical to that defined by PDBTM and the definition of rotational angle depends on the helical principal axis and the C_α_ vector of the first residue to its lipid-facing direction vector, we cannot simply assume their structural property is similar. Second, for a predicted 3D protein structure, the TM region information comes from the topology predictor, and the rotational angle of each TMH is established on the atoms within predicted TM region, not from the PDBTM. Therefore, we have to recalculate the rotational angles of predicted TMH for comparison. Finally, we ignored any predicted TMH which includes residues that do not have structural data within PDB entity, and 155 TMHs were left for comparison.

The Additional file 4: Table S2 and the Additional file 5: Table S3 provide sequences of predicted TMHs corresponding to TMHs annotated in PDBTM, observed angle defined by residues of predicted topology, predicted angle, and moment lengths. For all 155 TMHs, the MAAE of TOPCONS is 43.04˚ and MAAE of MemBrain is 56.59˚. These two tables demonstrate the ability of TMexpo to predict rotational angles based on predicted topology. Interestingly, the MAAE of 155 TMHs based on topology predicted by TOPCONS has better results than topology annotated in PDBTM. There are two possible explanations for this observation. First, TMHs predicted by TOPCONS is longer than annotated in PDBTM, and this may help calculating the helical principle axis. Second, we excluded TMHs that have partially incomplete structural data, and the performance of 155 TMHs would differ from the dataset of 188TMHs. We conclude that rotational angles calculated by both predicted TMHs and predicted rASA are still consistent with the observed rotational angles defined by the predicted TMH. Therefore, while TMH boundary is not perfectly predicted, the predicted rotational angle can still provide useful information to the interior side of a TM protein and constrain decoys of predicted 3D structure.

### An application to an amino acid antiporter, AdiC

We selected from our independent test set an amino acid antiporter, called AdiC, of E. coli strain O157:H7 to demonstrate how predicted rASA and rotational angle in the helical wheel presentation of TMHs facilitate the analysis of TM proteins. The E. coli strain O157:H7 is a pathogen and causes hemorrhagic diarrhea, and AdiC is a multi-spanning TM protein that enables E. coli to resist the acidic environment via exchanging extracellular arginine and intracellular agmatine [46,47]. An arginine-bounded structure of AdiC was solved and is coded as 3L1L [46] in PDB.

**Figure 1:**
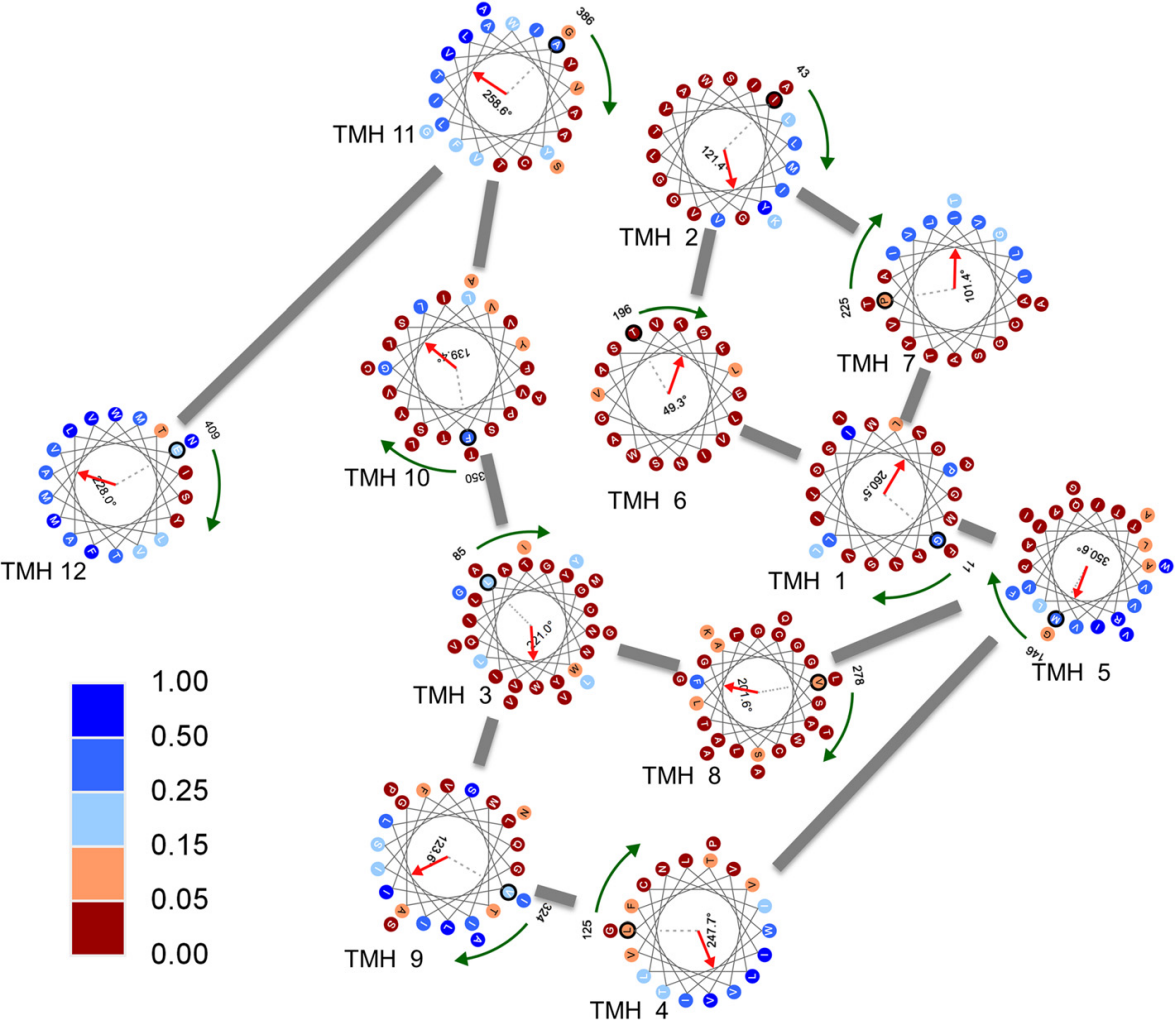
**Top view of the structure 3L1L shown by helical wheels, interhelical interactions and observed rASA of the TM residues.** Each TM residue is shown by a color-coded gradient scale of its observed rASA, with dark red and dark blue representing rASA value 1.0 for extremely buried and 0 for extremely exposed, respectively. The red arrow inside each helical wheel indicates the lipid-facing direction, and the green clock-wise arrow starts at the first amino acid.

The protein AdiC has 445 amino acids and contains 12 TMHs, including an inner layer containing 5 TMHs, i.e., transmembrane segments 1, 3, 6, 8 and 10 from the N-terminus, and an outer layer containing 7 TMHs, i.e., transmembrane segments 2, 4, 5, 7, 9, 11 and 12. Figure 1 illustrates the top view of 3L1L, in which each residue in a TMH is shown by a color-coded gradient scale of its observed rASA, with dark red and dark blue representing extremely buried and extremely exposed, respectively. The red arrow inside each helical wheel indicates the lipid-facing direction, and the green clock-wise arrow starts at the first amino acid. A gray line connects two TMHs if they have interhelical interactions with more than five VDWs as annotated in TMPad. It can be observed that most of residues in the five TMHs of the inner layer are colored as dark red, and the TMHs of the outer layer have residues in both exposed and buried sides colored by blue and red, respectively.

**Figure 2:**
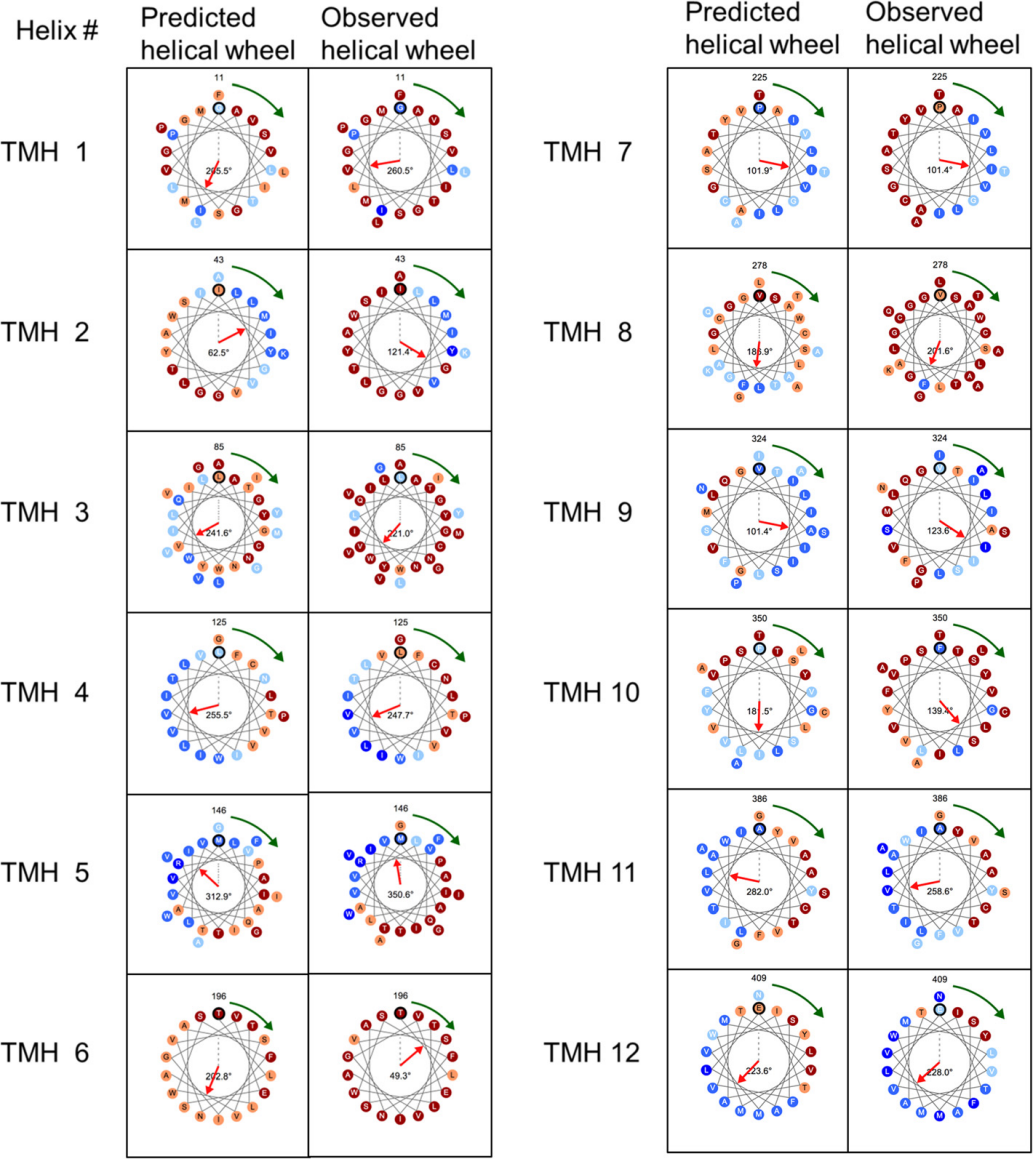
**Observed and predicted rASA and lipid-facing directions of the 12 TMHs in 3L1L represented by helical wheels.** The angular error of 12 TMHs ranges from 0.49˚ to 153.48˚; the MAAE of 12 TMHs is 36.72˚.

Interestingly, several binding sites and functional mutagenesis were discovered in TMHs of the inner layer. According to annotations in the UniProt/Swiss-Prot, the amino acids 22, 26, 93, 208 and 365 are substrate binding sites located in the TMHs 1, 3, 6 and 10 of the inner layer. Furthermore, four reported mutations, Y87A, Y93A, Y93K and Y365A, related to transporter activity also occur in TMHs 3 and 10. Therefore, TMHs of the inner layer are important to transporter function study. All of the above six residues are classified as buried in the crystallized structure 3L1L, and their observed rASA range from 0% to 2.13%. TMexpo predicted five of the above six residues as buried. Only one residue 87Y was predicted as marginally exposed and had 9.03% rASA. Figure 2 shows that the observed and predicted lipid-facing directions of the 12 TMHs are quite close. The MAAE of 12 TMHs is 36.72˚, and 7 out of 12 TMHs were accurately predicted with angular errors <30˚. The best angular error is 0.49˚ obtained by the linear TMH 7, and the worst angular error is 153.48˚ obtained by the kinked TMH 6. The PCC between the observed and predicted rASA of the entire 266 TM residues is 0.75; the MCC of the TMexpo’s classification of all the TM residues is 0.60. Our analysis shows that the predicted rASA of each residue could facilitate the design of site-directed mutagenesis experiments. Therefore, sequence-based prediction of both rotational angle and rASA can provide useful information in transporter function study, and thus contribute to experimental design to elucidate TM protein functions when high-resolution structure is unavailable.

### Interhelical contacts play an important role in relative accessible surface area prediction

To understand how intrachain interhelical interactions affect the burial status of residues is important for helical packing and stability of TM proteins [48-51]. At first we observed residues carrying more intrachain interhelical contacts with other TMHs tend to be buried. We investigated on the TMHs of the development set of 89 protein chains whether buried residues are hard to predict their rASA values. Using the annotations in TMPad, we separated residues into two sets according to different thresholds *c* of VDW contact numbers; one is called contact-enriched set of residues with at least *c* contacts, and the other set is called reference set consisting of the remaining residues. For each set, we calculated the PCC between the observed rASA and the predicted rASA obtained from LOOCV. The results are shown in Table 6, where different thresholds *c* = 1, *c* = 2, and *c* = 3 are considered. By comparing correlations of predicted rASA against observed rASA for different thresholds in LOOCV, we found that the set containing contact-enriched residues has lower correlation than the reference set by 40.66% when *c* = 3 and by 9.53% even when *c* = 1. This observation implies rASA of residues that have a large number of interhelical contacts may be more difficult to be predicted, and then the information of interhelical contacts should be integrated for improving rASA prediction.

**Table 6:** **Comparing Pearson correlation coefficients between contact-enriched set and reference set defined by different thresholds ( *c *)**

**Thresholds for contact-enriched set**	**PCC (number of residues) on contact-enriched set**	**PCC (number of residues) on reference set**
*c* ≥ 1	0.50 (4,447)	0.59 (4,078)
*c* ≥ 2	0.33 (1,981)	0.64 (6,544)
*c* ≥ 3	0.25 (671)	0.65 (7,854)

The first column stands for different thresholds, and the last two columns stand for Pearson correlation coefficients of transmembrane residues from the two different sets.

## Conclusions

Sequence-based prediction of both rotational angle and rASA can provide indispensable information for structure prediction when high-resolution structures are unavailable and contribute to experimental design to elucidate TM protein functions. In this paper, we present a novel concept of using lipid exposure to infer rotational angles and have developed a machine learning approach to predict rotational angles of TMHs. Significantly, using predicted rASA from our sequence-based model achieved an MAAE of 48.31˚ on the independent test set, which is better than that obtained by the best of the compared knowledge-based propensities (67.51˚). Furthermore, we demonstrate an application for structural analysis via an amino acid antiporter. We believe improving prediction of rotational angle can benefit the structure prediction because free modeling of TM protein structures is a tough task and reducing the number of packing arrangements is necessary.

## Methods

### Evaluation measures

The metric used for evaluating rotational angle prediction in this work is mean absolute angular error (MAAE). To evaluate the classification model, i.e., classifying burial and exposed status, we used the following performance measures, including Matthew’s correlation coefficient (MCC), accuracy, sensitivity, specificity, and precision. With respect to the regression model, i.e., rASA prediction, we used mean absolute error (MAE), root mean squared error (RMSE), and Pearson correlation coefficient (PCC).

We evaluate the MAAE, which is the average angular error for all TMHs. Specifically, we defined observed rotational angle as *x* and predicted one as *y* for each helix, and both of them range from 0˚ to 360˚. The absolute angular error *θ*_error_, ranging from 0˚ to 180˚, is defined as the difference between two rotational angles of TMHs as Equation 1. The extreme error between observed and predicted angles occurs when they indicate opposite direction i.e. 180˚. The MAAE is defined as the average of all absolute angular error *θ*_error_ within *n* helices as Equation 2.

(1) $$\theta_{\mathrm{error}}\left(x,y\right)=\left\{\begin{array}{cc} \left|x-y\right| & ,\left|x-y\right|\leq 180\\ 360-\left|x-y\right| & ,\left|x-y\right|>180 \end{array}\right)$$

(2) $$\mathrm{ΜΑΑΕ}=\frac{1}{n}\sum_{i=1}^{n}\theta_{\mathrm{error}}\left(x_{i},y_{i}\right)$$

Let *predicted*_*i*_ and *observed*_*i*_ denote the predicted and observed rASA, respectively, of the *i*_*th*_ TMH in the sample dataset. MAE and RMSE are defined as Equations 3 and 4.

(3) $$\mathrm{MAE}=\frac{1}{n}\sum_{i=1}^{n}\left|\mathrm{predicte}d_{i}-\mathrm{observe}d_{i}\right|$$

(4) $$\mathrm{RMSE}=\sqrt{\frac{1}{n}\sum_{i=1}^{n}{\left(\mathrm{predicte}d_{i}-\mathrm{observe}d_{i}\right)}^{2}}$$

We defined exposed residues as positive data and buried residues as negative data, and consequently MCC are defined as Equation 5.

(5) $$\mathrm{MCC}=\left(\mathrm{TP}*\mathrm{TN}-\mathrm{FP}*\mathrm{FN}\right)/\sqrt{\left(\mathrm{TP}+\mathrm{FP}\right)\left(\mathrm{TP}+\mathrm{FN}\right)\left(\mathrm{TN}+\mathrm{FN}\right)\left(\mathrm{TN}+\mathrm{FP}\right)}$$

See the Additional file 6: Table S4 for definitions of Pearson correlation coefficient, accuracy, sensitivity, specificity and precision.

### Data preparation

We retrieved the α-helical TM proteins from the PDBTM database [52,53], a collection of automatically identified TM proteins from the Protein Data Bank (PDB), and obtained 4,202 chains from 1,174 PDB entities. Among them, we only kept multi-spanning α-helical TM protein structures solved by X-rays with a resolution less than 4 Å, and then 2,305 protein chains remained. Next, the 2,305 chains were reduced at mutual sequence identity of less than 30% using CD-HIT [54]. Finally, the representative 110 multi-spanning protein chains listed in Table 7 were divided into two datasets for model development and independent test. We took 89 (554 TMHs) from 110 TM protein chains solved before October 20, 2008 as the development set and optimized the parameters in the models by leave-one-out cross-validation (LOOCV). The other 21 (188 TM helices) protein chains solved after October 20, 2008 were used for independent test. The two datasets are provided on the TMexpo server website (http://bio-cluster.iis.sinica.edu.tw/TMexpo) and also in the Additional file 7: Dataset S3.

**Table 7:** The list of all protein chains (PDB:Chain) included in the development set and the independent test set

**Development set**								**Independent test set**	
1E7P:C	1LNQ:A	1YCE:A	2 F93:A	2PNO:A	2WIT:A	3B8C:A	3EH4:A	2XQ2:A	3MP7:A
1EYS:L	1ORQ:C	1YEW:C	2GFP:A	2Q67:A	2WSW:A	3CHX:A	3EHB:B	2XUT:A	3NYM:A
1EYS:M	1P7B:A	1YQ3:C	2GIF:A	2Q7R:A	2YVX:A	3CHX:B	3G5U:A	3KBC:A	3O0R:B
1FFT:A	1PV6:A	1YQ3:D	2IUB:A	2QJY:A	2Z73:A	3CIR:C	3GIA:A	3KCU:A	3O7P:A
1FFT:B	1PW4:A	1ZCD:A	2JLO:A	2R6G:F	2ZD9:A	3CIR:D	3H90:A	3KG2:A	3OE6:A
1FFT:C	1QLE:C	2A65:A	2NMR:A	2R6G:G	2ZJS:Y	3CN5:A	3H9V:A	3KJ6:A	3ORG:A
1FX8:A	1S5L:B	2AXT:A	2NQ2:A	2R9R:B	2ZW3:A	3D31:C	3HD6:A	3KP9:A	3P4W:A
1GZM:A	1S5L:C	2AXT:D	2NR9:A	2VL0:A	2ZXE:A	3DDL:A	3IJ4:A	3L1L:A	3P5N:A
1JB0:K	1S5L:Z	2BL2:A	2OAR:A	2VPZ:C	3A7K:A	3DHW:A	3JYC:A	3 M71:A	3PJZ:A
1JV6:A	1XIO:A	2C3E:A	2OAU:A	2WDV:C	3B4R:A	3E9J:C	3 K07:A	3MK7:A	
1KPK:A	1Y4Z:C	2E75:B	2ONJ:A	2WDV:D	3B5D:A	3EFF:K	3K3F:A	3MK7:C	
1KQG:C								3MKT:A	

### Calculation of relative accessible surface area from structures

To calculate lipid exposure or exposed area of a structure, we used NACCESS program [19,29] with the probe radius set to 2.0 Å. The size of probe radius 2.0 Å was selected to mimic the -CH2 of hydrocarbon chains, and it is identical to that used in Yuan et al. [20], Illergård et al. [21] and Lo et al. [37]. The ASA for a residue was the sum of ASA from all atoms belonging to that residue. To extract the helical boundaries from the protein chains, we used the annotations of PDBTM. From the 89 protein chains in the development set, we obtained a total of 10,441 residues in TM domain. For the independent test set of newly solved proteins, we obtained 3,581 residues in TM domain. To annotate missing residues and missing atoms, we used PDB Validation Suite [55]. In order to obtain rASA as a normalized measure for a TM residue, we divided the ASA values by their reference values in a Gly-X-Gly tripeptide in an extended conformation. The reference values were derived from Samantha et al. [56]. To classify burial status of each residue for model training and testing, we followed the rASA threshold defined in Miller et al.’s work [57], i.e., rASA <5% to characterize buried residues and otherwise exposed, though different thresholds have been used in the literature.

### An SVM-based predictor for lipid exposure of TM helices

#### Model development

We proposed residue-wise predictors based on support vector machines (SVMs), i.e., an SVM classifier to predict the burial/exposed status and a support vector regression (SVR) model to predict rASA values of each residue in TM domain. Specifically, C-SVC and epsilon-SVR implemented in LIBSVM [58] were used to develop the models, and both of them used the RBF kernel function. The parameters of the models were optimized by chain-wise LOOCV procedure on the development set. In LOOCV procedure, the best set of parameters to train the burial/exposed status classification model is of cost *c* = 2^1^ and gamma *g* = 2^-4^; and the best set of parameters to train the real-number rASA regression model is of cost *c* = 2^-1^, gamma *g* = 2^-5^, loss function *p* = 10^-3^ and tolerance of termination criterion *e* = 10^-2^. Details of LOOCV performances can be obtained in the Additional file 8: Text S1.

Given a TM domain of a protein chain, each residue to be predicted was located at the center of a sliding window of length 17 and features were generated according to the 17-mer sequence. To train the classification model, exposed residues with label “E” were considered as positive data, and buried residues with label “B” as negative data. To train the regression model, the input was taken from the real-number rASA. We searched parameters by LOOCV procedure for the classification model and the regression model based on optimizing the MCC and the PCC, respectively. We did not directly predict ASA values because they are not normalized in a zero to one interval and this could produce bias in the presence of an outlier.

In training and testing, we excluded residues that participate in interchain contacts and the rationale is as follows: A sequence-based rASA predictor, which accepts the sequence of a structural subunit as input, can only describe structural properties of one subunit, not of the complete structure. Thus predicted rASA of those residues may be drastically different depending on their locations in the interacting interfaces. In the case of residues residing on the interchain surface, we observed rASA of these residues in a single chain may be significantly different from those seen in the complete structure with multiple subunits. Out of 110 representative protein chains used in our work, 86 protein chains are multimeric. Among all of their 9,800 TM residues without any missing atom, 2,167 (22.11%) residues have two different rASA values calculated from the single subunit and the complete structure, respectively; and the former-derived rASA is always larger than the latter-derived rASA. Notably, the maximum and average differences of the two rASA values of these 2,167 residues are 82.28% and 23.43%, respectively. Furthermore, 831 out of the 2,167 residues would be assigned inconsistent burial/exposed status according to their two different rASA values. In other words, 8.48% of the overall 9,800 transmembrane residues were considered as exposed from the perspective of a single subunit but turned out to be buried in their complete structures. For example, in 2OAR:A, 41 residues of 52 TM residues have different rASA values, and 13 residues are calculated as being exposed in the single chain but as being buried in the complete structure. It is noteworthy that the 60S (i.e., 37S by PDB indexing) and the 45 V (i.e., 22 V by PDB indexing) have drastic differences in their rASA values, i.e., 60.77% vs. 2.42% and 60.02% vs. 1.68%, as calculated by single chain and by complete structure, respectively. Since we did not know the native state of amino acids lying on the interchain surface, we excluded these residues from our training and testing data. For each protein chain, we calculated rASA for both single subunit structure and complete structure. Later, we excluded residues which were not identical in rASA by comparing the above two calculations, and also excluded residues that were missing partial or entire atoms.

In the testing stage, we performed a simple post-processing by rounding off their upper and lower bound to 1 and 0 because rASA values are contained in this interval. To derive the ASA values for each residue, we multiplied the predicted rASA values by the reference values [56].

#### Input features for predictors

In the design of TMexpo, we did not use a specific feature selection technique, and all the features used in TMexpo belong to one of the three feature groups. The first group is about interhelical contacts, specifically volume, polarity, charge and residue interhelical contact propensity. Since we have observed buried residues tend to have more interhelical contacts, and therefore we examined features related to interhelical contacts. For example, the well-known GxxxG motif can be regarded as small-xxx-small motifs [39], and we use volume profiles to incorporate such feature in the machine learning model. The polarity and charge can also be seen as features related to hydrogen bonding [50] and cation-pi interaction [59], respectively. To encode features into TMexpo, the volume [60] of each residue was divided by their maximum value 237.2 of tyrosine. The polarity was also encoded by the sigmoidal functions 1–1/(1 + *e*^*-po*^), where *po* denotes the mean residue polarity calculated by Radzicka and Wolfenden’s method [61]. We defined positively charged residues as 1, neutral residues as 0.5, and negatively charged residues as 0 based on the index used by Klein et al. [62]. The residue interhelical contact propensity were developed by Lo et al. [38], and we used in TMexpo the normalized propensity by division of the maximum value 1.43 of cysteine.

The second group provides evolutionary information as position-specific scoring matrix (PSSM) profiles and conservation score to machine learning model. Evolutionary information is an important feature and has been incorporated in interhelical interaction predictors [38,63]. To encode PSSM as features, the matrix was generated by performing PSI-BLAST against NCBI’s non-redundant database. This feature of a 17-mer peptide was encoded by a vector of size 17 × 20, where each entry was normalized by 1- 1/(1 + *e*^*-PSSM*^). The conservation score was calculated by an algorithm developed by Capra and Singh [64] on the multiple sequence alignment generated by MAFFT [65,66] based on the 17-mer peptide. We used the raw scores without using the local Z-score transformation described in their method.

The third group includes the TMH insertion energy, amphiphilicity of residues and turn propensities, which relate to structural information. The first two features can reveal residue position toward hydrophobic membrane or water interface, which is akin to Zpred features used in MPRAP that directly predict relative position from the center of membrane for each residue. The position-specific free energy of TMH insertion, termed as “free energy” to describe the hydrophobic core, was encoded by a sigmoidal function as 1-1/(1 + *e*^*-energy*^), where *energy* denotes the free energy of TMH insertion estimated by Hessa et al.’s method [67]. The amphiphilicity was encoded by the sigmoidal functions 1–1/(1 + *e*^*am*^), where *am* denotes the amphiphilicity derived by Mitaku et al.’s method [68]. We also considered the helix turn propensities in order to capture sequence information related to tight turns in naturally occurring TM helices from Monné et al. [69]. This feature was normalized propensities to [0, 1] by dividing the maximum value 2.7 of proline.

All of the above features were normalized to a closed [0, 1] interval. A feature value close to 1 means the corresponding residue is more hydrophobic, more amphiphilic, higher polarity, positively charged, larger volume, more conserved, tends to have turns and interhelical contacts. We filled 0.5 as features for nonexistent residues in windows, except charge, interhelical contacts, and volume, we filled zeroes.

### Predicting rotational angle based on relative accessible surface area

#### Determination of rotational angle of a transmembrane helix

The rotational angle of a TMH was calculated as follows: First, we removed atoms which were annotated outside the membrane. Second, we computed the helical principal axis of the TMH of interest and aligned it with the *z*-axis with the N-terminal facing the screen, creating a top-view of the protein with respect to the target helix. Third, we identified the geometric centers of the molecule and each of the individual helices in the two-dimensional plane from the average *x* and *y* coordinates of C_α_ in the constituent TMH residues. We defined the lipid-facing direction of a TMH as in the opposite direction circumscribed by the geometric center of the target TMH connects to the molecular geometric center of the protein chain unit. The rotational angle of the target helix was measured as the angle rotated from the C_α_ vector of the first residue to its lipid-facing direction vector by clockwise motion viewed from the helix N-terminal to C-terminal. The angle ranges from 0˚ to 360˚.

#### Calculation of relative accessible surface area moment direction

The moment *M* was computed for several propensity scales with the moment length |*M*| and the moment direction *θ* relative to the angular direction of the C_α_ vector of the first residue of the TMH [9]. In this work, we used the rASA moment direction to describe lipid-facing direction and used the definition and post-processing techniques for both outliers and ramps described in Donnelly et al. [70] to resolve the moment direction *θ*. They were defined as follows:

(6) $$\begin{array}{l} x=\sum_{i=1}^{n}\left(\mathrm{rAS}A_{i}\cos\theta_{i}\right),\;\\ y=\sum_{i=1}^{n}\left(\mathrm{rAS}A_{i}\sin\theta_{i}\right),\;\theta_{i}=\left(i-1\right)*100 \end{array}$$

(7) $$\left|M\right|=\sqrt{x^{2}+y^{2}}$$

(8) $$\gamma =\arccos\left(\frac{x}{\left|M\right|}\right)$$

(9) $$\theta =\left\{\begin{array}{cc} \gamma & ,y\geq 0\\ 360-\gamma & ,y<0 \end{array}\right)$$

For each residue, the rASA values can be seen as degree of directional lipid-facing. Therefore, for one TMH, the summation of all TMH residues’ lipid-facing tendency can characterize its rotational angle. In Equation 6, the *x* and the *y* terms are the vector summation of over *n* residues in a TMH. The moment length |*M*| is defined as Equation 7. The angle *γ* was solved first by inverse cosine function as Equation 8, and we determined moment direction *θ* by taking (360 - *γ*) as a result as Equation 9 if the sign of *y* term is negative.

#### Predicting rotational angles of transmembrane helices

**Figure 3:**
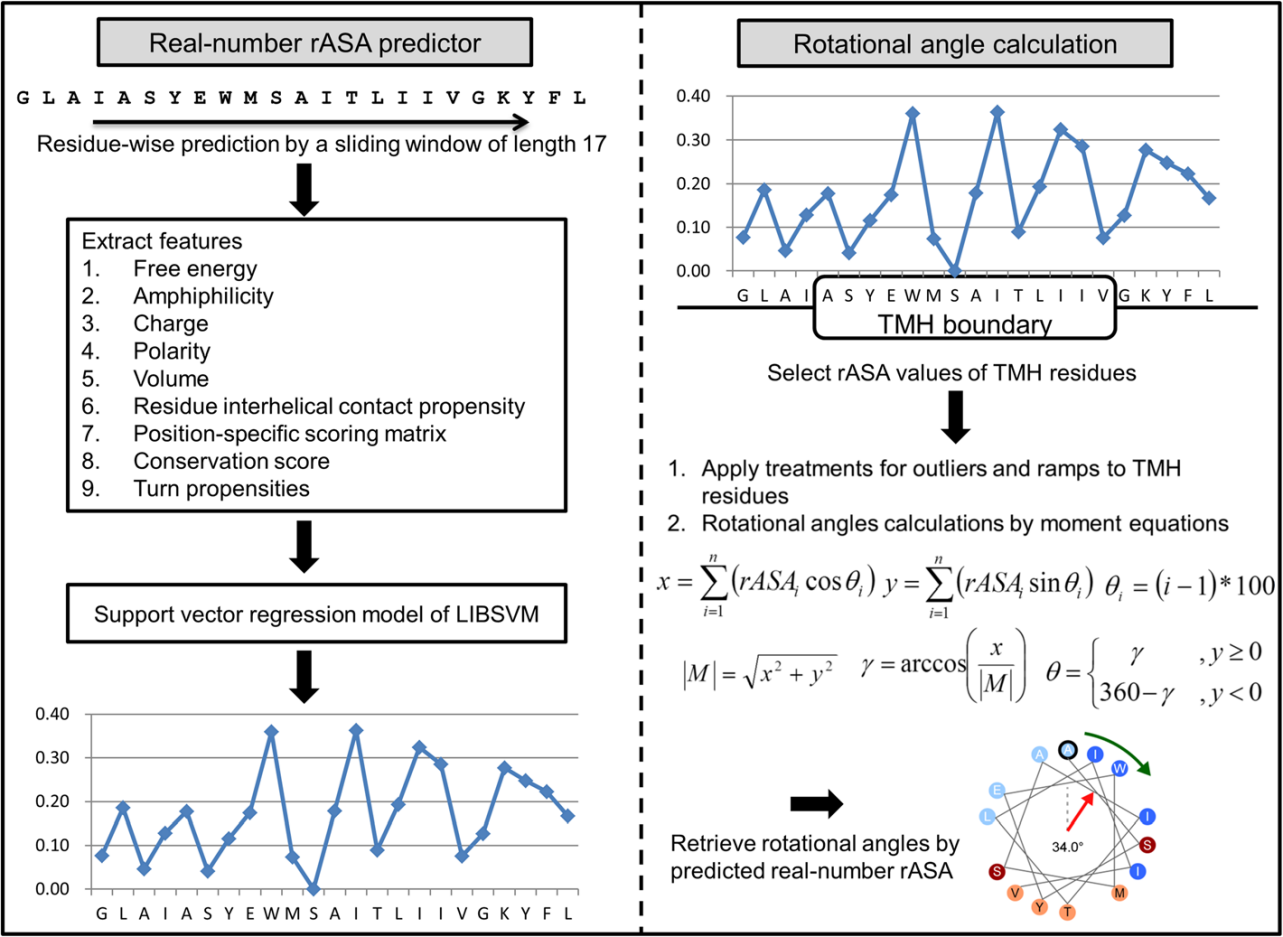
The workflow of TMexpo for predicting rotational angles from sequences.

In a previous study, propensity values, such as hydrophobicity or lipid-facing propensities, were used to describe the moment direction [9,11,12,14]. The procedure of calculating rotational angles of TMHs from predicted rASA values is described as follows: First, we predict rASA values from sequence by TMexpo. Second, we select each TMH according to TM domain annotated by PDBTM. To predict rotational angle, we further assign predicted rASA values of each TMH using Equation 6 and Equation 7 to obtain predicted rASA moment length ($\left|\hat{M}\right|$). Finally, we calculate the angles of moments for each TMH by Equation 8 and Equation 9, then take these angles as predicted rotational angles as lipid-facing directions of TMHs. Figure 3 illustrates the workflow of TMexpo for predicting rotational angles from sequences. We followed the above procedure for several propensity scales or rASA to obtain and compare the predicted rotational angles.

## Abbreviations

ASA: Accessible surface area; LOOCV: Leave-one-out cross validation; MCC: Matthew’s correlation coefficient; MAAE: Mean absolute angular error; MAE: Mean absolute error; PCC: Pearson correlation coefficient; PDB: Protein data bank; rASA: Relative accessible surface area; RMSE: Root mean squared error; SVM: Support vector machine; SVR: Support vector regression; TM: Transmembrane; TMH: Transmembrane helix; VDW: Van der Waal’s.

## Competing interests

The authors declare that they have no competing interests.

## Authors’ contributions

JSL, CCW, AL and TYS conceived and designed the experiments. JSL and CCW performed the experiments. JSL analyzed the data. JSL and CCW developed analysis tools and web services. JSL, CCW, AL, TYS and WLH wrote the manuscript. All authors have read and approved the manuscript.

## Supplementary Material

Additional file 1: Dataset S1 The rASA prediction results of the development set and the independent test set. Click here for file

Additional file 2: Table S1 Sequence, observed angle, predicted angle, moment lengths and MAAE of the 188 TMHs in the independent test set of 21 proteins. Click here for file

Additional file 3: Dataset S2 Signal peptide and topology prediction results of the independent test set from SVMSignal, TOPCONS and MemBrain. Click here for file

Additional file 4: Table S2 Sequences, observed angles defined by residues of predicted topology (by TOPCONS), predicted angles, moment lengths and MAAE on the 155 TMHs of the independent test set. Click here for file

Additional file 5: Table S3 Sequences, observed angles defined by residues of predicted topology (by MemBrain), predicted angles, moment lengths and MAAE on the 155 TMHs of the independent test set. Click here for file

Additional file 6: Table S4 Evaluation measures used in this work. Click here for file

Additional file 7: Dataset S3 The representative 110 multi-spanning protein chains used in this work. Click here for file

Additional file 8: Text S1 Details of LOOCV performances during model development. Click here for file
