# Supplementary material for: Lipid exposure prediction enhances the inference of rotational angles of transmembrane helices
Source: BMC Bioinformatics. 2013 Oct 11;14:304. doi: 10.1186/1471-2105-14-304 (PMC3854514; doi:10.1186/1471-2105-14-304)
Supplement: Additional file 2: Table S1 — Sequence, observed angle, predicted angle, moment lengths and MAAE of the 188 TMHs in the independent test set of 21 proteins. [file 1471-2105-14-304-S2.docx]

Table S1. Sequence, observed angle, predicted angle, moment lengths and MAAE of the 188 TMHs on the independent test set of 21 proteins.

| PDB:Chain | TM helix sequence | observed angle | predicted angle | angular error |  | PDB:Chain | TM helix sequence | observed angle | predicted angle | angular error |  |
| --- | --- | --- | --- | --- | --- | --- | --- | --- | --- | --- | --- |
| (MAAE) |  |  |  |  |  | (MAAE) |  |  |  |  |  |
| **2XQ2:A** | AIYVAIIIGVGLWV | 62.77 | 67.05 | 4.28 | 2.04 | **3KBC:A** | QKILIGLILGAIVGLI | 73.46 | 33.53 | 39.93 | 1.17 |
| (45.33) | AVGASLIAANISAEQF | 318.77 | 226.68 | 92.09 | 0.33 | (45.38) | VKPFGDLFVRLLCMLVMPIVFASL | 217.20 | 227.72 | 10.52 | 0.87 |
|  | ASYEWMSAITLIIV | 50.83 | 34.02 | 16.81 | 0.88 |  | AFAVTLGIIMARLFNPG | 106.30 | 97.86 | 8.43 | 0.86 |
|  | LAVFWISLYIFVNLTSVLYLG | 198.94 | 196.57 | 2.36 | 0.34 |  | VHILLDIVP | 35.08 | 352.99 | 42.09 | 0.43 |
|  | GLALFALVYSIYGG | 345.17 | 320.92 | 24.25 | 1.01 |  | QVLPTIFFAIIL | 273.16 | 221.73 | 51.44 | 0.38 |
|  | VWTDVIQVFFLVLGG | 116.95 | 88.35 | 28.60 | 0.96 |  | VMQYAPIGVFALIAYVM | 231.75 | 293.20 | 61.45 | 0.61 |
|  | AVLIGGLWVANLYYWGFNQ | 234.97 | 6.61 | 131.63 | 0.35 |  | VGELAKVTAAVYVGLTLQ | 75.91 | 65.22 | 10.69 | 1.40 |
|  | VFAAFLALIVPFLV | 34.73 | 47.32 | 12.59 | 1.32 |  | GTALYQGVATFFIA | 267.85 | 339.32 | 71.47 | 0.13 |
|  | ALAAAIVSSLASMLNSTA | 243.85 | 145.68 | 98.17 | 0.82 |  | TVGQQLTIVLTAVLASIGT | 194.19 | 128.07 | 66.12 | 0.56 |
|  | RTAAVVALIIAALIA | 90.02 | 115.31 | 25.29 | 1.90 |  | GAGAIMLCMVLHSVGLPLT | 94.44 | 3.52 | 90.92 | 0.39 |
|  | QYIQEYTGLVSPGILA | 99.61 | 145.15 | 45.55 | 0.25 |  | AAAYAMILGIDAILDMGR | 144.91 | 191.00 | 46.09 | 0.14 |
|  | VVASIPFALFLKFM | 194.71 | 156.50 | 38.21 | 1.02 | **3KCU:A** | YLAITAGVFISIAFVFYITATTGT | 49.84 | 331.07 | 78.77 | 0.76 |
|  | FMDQMLYTLLFTMVV | 242.47 | 168.12 | 74.34 | 1.02 | (81.20) | PFGMAKLVGGICFSLGLILCV | 270.63 | 296.59 | 25.96 | 0.78 |
|  | AAYGIMIVLAVLYT | 238.76 | 250.02 | 11.26 | 0.84 |  | LNVYFGNLVGALLFVLLMWLSGEYMTA | 124.59 | 60.77 | 63.82 | 2.04 |
|  | GVMAGVIGTILLISYGIK | 237.67 | 312.17 | 74.50 | 0.37 |  | GLNVLQTADHKVHHTFIEAVCLGILANLMVCLAV | 201.92 | 221.76 | 19.84 | 1.20 |
| **2XUT:A** | ASEACERFSFYGMRNILTPFL | 42.13 | 324.24 | 77.89 | 0.73 |  | FIMVLPVAMFVA | 157.93 | 334.34 | 176.41 | 0.57 |
| (39.50) | VAKDVFHSFVIGVYFFPLLG | 20.31 | 293.48 | 86.83 | 0.88 |  | SIANMFMIPMGIVIRDF | 88.53 | 118.36 | 29.83 | 0.40 |
|  | TILWLSLIYCVGHAFLAIFE | 239.21 | 257.48 | 18.27 | 1.76 |  | LTVMNFITDNLIPVTIGNIIGGGLL | 211.40 | 37.62 | 173.78 | 0.94 |
|  | GFYTGLFLIALGSGGIKP | 328.39 | 272.54 | 55.84 | 0.60 | **3KG2:A** | AYEIWMCIVFAYIGVSVVLF | 239.01 | 143.26 | 95.76 | 1.55 |
|  | YFTINFGSFFASLSMPLLL | 166.20 | 164.93 | 1.27 | 1.31 | (47.98) | LWFSLGAFMQ | 77.10 | 15.33 | 61.76 | 0.57 |
|  | VAFGIPGVLMFVATVFFWL | 9.27 | 338.61 | 30.66 | 1.37 |  | SGRIVGGVWWFFTLIIISSYT | 274.85 | 245.04 | 29.81 | 1.21 |
|  | IGGVSAAYALVNIPTL | 270.12 | 309.33 | 39.21 | 0.18 |  | VAGVFYILVGGLGLAMLVAL | 117.93 | 122.54 | 4.61 | 1.38 |
|  | IVAGLCCAMVLVMGFVG | 342.93 | 69.91 | 86.98 | 0.53 | **3KJ6:A** | VMSLIVLAIVFGNVLVITAIA | 287.39 | 294.98 | 7.59 | 1.31 |
|  | ALVTPFWSLFDQKASTWILQA | 122.86 | 115.17 | 7.69 | 0.19 | (37.44) | YFITSLACADLVMGLAVVP | 318.88 | 348.66 | 29.78 | 0.68 |
|  | FEPAMMQALNPLLVMLLI | 5.73 | 49.27 | 43.54 | 0.58 |  | IDVLCVTASIETLCVIAVD | 265.93 | 321.95 | 56.02 | 0.87 |
|  | GAGIAITGLSWIVVGTIQ | 33.27 | 66.73 | 33.47 | 1.71 |  | ARVIILMVWIVSGLT | 205.30 | 184.91 | 20.39 | 1.32 |
|  | SIFWQILPYALLTFGEVLVS | 200.32 | 193.72 | 6.60 | 0.66 |  | SIVSFYVPLVIMVFVYSR | 100.84 | 188.15 | 87.30 | 1.36 |
|  | FWTLSVTVGNLWVLLANVSV | 26.16 | 335.26 | 50.90 | 1.20 |  | LGIIMGTFTLCWLPFFI | 157.87 | 135.17 | 22.70 | 0.66 |
|  | FQMFFFAGFAILAAIVF | 317.72 | 331.55 | 13.83 | 0.91 |  | LNWIGYVNSGFNPLIYC | 286.28 | 247.97 | 38.32 | 0.95 |
|  |  |  |  |  |  | **3KP9:A** | ILAILAGLGSLLTAYLTYT | 254.55 | 292.34 | 37.79 | 2.02 |
|  |  |  |  |  |  | (46.26) | AEFLGIPTAAVGLLGFLGVLALAVL | 141.65 | 207.78 | 66.12 | 1.07 |
|  |  |  |  |  |  |  | LFGLVSAMTAFEMYMLYLMVA | 137.87 | 191.01 | 53.13 | 1.83 |
|  |  |  |  |  |  |  | CMYCTTAIILVAGLGLVTV | 78.48 | 115.19 | 36.70 | 1.45 |
|  |  |  |  |  |  |  | FSYILVAFLTLVTTIGVYANQ | 35.75 | 358.20 | 37.54 | 0.83 |
|  |  |  |  |  |  |  |  |  |  |  |  |
|  |  |  |  |  |  |  |  |  |  |  |  |
|  |  |  |  |  |  |  |  |  |  |  |  |

| PDB:Chain | TM helix sequence | observed angle | predicted angle | angular error |  | PDB:Chain | TM helix sequence | observed angle | predicted angle | angular error |  |
| --- | --- | --- | --- | --- | --- | --- | --- | --- | --- | --- | --- |
| (MAAE) |  |  |  |  |  | (MAAE) |  |  |  |  |  |
| **3L1L:A** | GLIPVTLMVSGAIMGSGVFLLP | 260.47 | 205.47 | 55.01 | 0.46 | **3MK7:C** | LTLGTIVALFWLIFA | 306.72 | 254.45 | 52.27 | 1.70 |
| (36.72) | IYGWLVTIIGALGLSMVYAK | 121.40 | 62.50 | 58.90 | 1.65 | (58.33) | RWWFLLFIGTLVFGI | 80.20 | 15.81 | 64.38 | 1.60 |
|  | LGYQTNVLYWLACWIGNIAMVVIGVGYL | 221.01 | 241.58 | 20.57 | 0.89 | **3MKT:A** | ATPVLIASVAQTGMGFVD | 144.31 | 105.62 | 38.69 | 1.29 |
|  | LTITCVVVLWIFVLLNIVGP | 247.74 | 255.50 | 7.76 | 1.61 | (60.34) | WLPSILFGVGLLMALVPV | 173.50 | 285.81 | 112.32 | 0.32 |
|  | MITRVQAVATVLALIPIVGIAVFGW | 350.60 | 312.92 | 37.68 | 2.63 |  | QGLILALLVSVPIIAVLF | 282.09 | 327.43 | 45.34 | 1.74 |
|  | TLNVTLWSFIGVESASVA | 49.33 | 202.81 | 153.48 | 0.43 |  | MHAVIFAVPAYLLFQALRSF | 274.84 | 95.07 | 179.78 | 0.72 |
|  | PIATIGGVLIAAVCYVLSTTA | 101.39 | 101.88 | 0.49 | 1.62 |  | KPAMVIGFIGLLLNIPL | 33.93 | 43.41 | 9.48 | 1.62 |
|  | VSFCAAAGCLGSLGGWTLLAGQTAK | 201.60 | 186.94 | 14.66 | 0.82 |  | ATAIVYWIMLLLLLFYIV | 242.67 | 282.04 | 39.37 | 1.55 |
|  | VAGLIIVGILMTIFQLSSISPNA | 123.65 | 101.42 | 22.23 | 1.48 |  | IRLFRLGFPVAAALFFEVTLFAVVA | 48.13 | 9.33 | 38.80 | 1.69 |
|  | FGLVSSVSVIFTLVPYLYTCAAL | 139.42 | 181.54 | 42.12 | 1.25 |  | ALNFSSLVFMFPMSIGAA | 277.85 | 313.69 | 35.84 | 0.48 |
|  | AYLAVTTIAFLYCIWAVVGSGA | 258.57 | 281.98 | 23.42 | 1.91 |  | GLATACITALLTVLFREQIA | 189.66 | 197.22 | 7.56 | 1.23 |
|  | EVMWSFVTLMVITAMYALN | 227.95 | 223.57 | 4.38 | 1.60 |  | QLLLFAAIYQCMDAVQVVAAGS | 274.85 | 319.69 | 44.84 | 0.88 |
| **3M71:A** | GYFGIPLGLAALSLAW | 181.38 | 167.78 | 13.61 | 0.21 |  | AIFHRTFISYWVLGLPTGYILGMT | 134.55 | 20.58 | 113.98 | 1.08 |
| (39.19) | SDVLGIVASAVWILFILMYAY | 159.91 | 183.80 | 23.89 | 2.22 |  | GFWLGFIIGLSAAALML | 28.07 | 329.96 | 58.10 | 0.63 |
|  | FIALIPITTMLVGDIL | 159.05 | 99.68 | 59.37 | 0.32 | **3MP7:A** | GVALILYYVLAEIPV | 87.05 | 46.08 | 40.97 | 1.27 |
|  | AEVLIWIGTIGQLLFSTLRVS | 198.45 | 213.60 | 15.15 | 1.64 | (48.75) | ILTLGIGPIVTAGIIL | 338.58 | 181.94 | 156.64 | 0.18 |
|  | SFYLPAVAANFTSA | 167.43 | 280.27 | 112.84 | 0.22 |  | VFSVFMCFFEAAVWI | 337.81 | 24.07 | 46.26 | 1.20 |
|  | YLFFGAGMIAWIIFEPVLL | 73.17 | 77.79 | 4.62 | 1.32 |  | LMILQLAMGGIVLIILDE | 317.35 | 308.66 | 8.69 | 1.47 |
|  | MGIVLAPAFVCVSAY | 290.77 | 16.64 | 85.87 | 0.81 |  | ISLFIAAGVSQTILTRSLNP | 228.20 | 70.12 | 158.07 | 0.18 |
|  | AKILWGYGFLQLFFLLRLF | 149.15 | 137.00 | 12.15 | 1.01 |  | DMLSVVATIVVFFIVVYF | 161.70 | 155.07 | 6.62 | 1.95 |
|  | GLWAFSFGLASMANSA | 232.75 | 177.98 | 54.77 | 0.09 |  | NIPIILTFALYANIQLWA | 180.18 | 190.26 | 10.09 | 0.63 |
|  | SIFAFVFSNVMIGLLVLMTIYKLT | 165.75 | 175.39 | 9.64 | 2.65 |  | VRAIVYLILTVIFSLLFG | 346.39 | 359.96 | 13.57 | 1.73 |
| **3MK7:A** | QFAIMTVVWGIVGMGLGV | 291.62 | 330.03 | 38.41 | 1.29 |  | GSLTVALIAVLADFL | 229.85 | 235.27 | 5.42 | 1.39 |
| (49.09) | HTNAVIFAFGGCALFATS | 285.17 | 219.33 | 65.84 | 0.40 |  | ALGTGTGILLTVGIL | 333.85 | 292.64 | 41.21 | 0.19 |
|  | LAAFTFWGWQLVILLAA | 289.75 | 273.94 | 15.80 | 1.34 | **3NYM:A** | NEFKEDLKNFLDYMEVCQLAL | 337.90 | 24.10 | 46.20 | 0.38 |
|  | IDILITIVWVAYAVVFF | 234.56 | 256.68 | 22.12 | 0.94 | (110.08) | IASNSLFMAMIYAGNLSLIFD | 210.14 | 36.17 | 173.97 | 0.46 |
|  | WFFGAFILTVAILHV | 305.15 | 248.13 | 57.02 | 0.21 |  |  |  |  |  |  |
|  | HNAVGFFLTAGFLGIM | 45.93 | 243.75 | 162.18 | 0.27 |  |  |  |  |  |  |
|  | IVHFWALITVYIWA | 68.08 | 72.94 | 4.86 | 1.02 |  |  |  |  |  |  |
|  | MSLILLAPSWGGMI | 241.27 | 211.81 | 29.46 | 1.14 |  |  |  |  |  |  |
|  | FLVVSLAFYGMSTFEGPM | 223.28 | 226.78 | 3.50 | 0.65 |  |  |  |  |  |  |
|  | GHVHAGALGWVAMVSI | 12.28 | 222.72 | 149.56 | 0.79 |  |  |  |  |  |  |
|  | THFWLATIGTVLYIA | 318.12 | 323.84 | 5.73 | 1.61 |  |  |  |  |  |  |
|  | RMIGGAIFFAGMLVMAY | 187.40 | 152.76 | 34.64 | 2.05 |  |  |  |  |  |  |
|  |  |  |  |  |  |  |  |  |  |  |  |
|  |  |  |  |  |  |  |  |  |  |  |  |
|  |  |  |  |  |  |  |  |  |  |  |  |

| PDB:Chain | TM helix sequence | observed angle | predicted angle | angular error |  | PDB:Chain | TM helix sequence | observed angle | predicted angle | angular error |  |
| --- | --- | --- | --- | --- | --- | --- | --- | --- | --- | --- | --- |
| (MAAE) |  |  |  |  |  | (MAAE) |  |  |  |  |  |
| **3O0R:B** | YFVFALILFVGQILFGLIMGL | 222.92 | 237.47 | 14.55 | 2.08 | **3ORG:A** | LRLVCFLTLLGVTAALFIFAVDLAVHGLE | 32.68 | 66.64 | 33.97 | 1.21 |
| (55.12) | ARMVHTNLLIVWLLFGFMGAAY | 299.87 | 181.59 | 118.28 | 0.40 | (51.15) | VSGVALCLLSTFWCAVLST | 351.66 | 40.08 | 48.42 | 1.56 |
|  | LAWILFWVFAAAGVLTILGYLL | 259.19 | 282.65 | 23.46 | 1.65 |  | RVLFAKALGLICAIGG | 338.42 | 305.73 | 32.69 | 0.73 |
|  | FLEQPTISKAGIVIVALGFLFNVGM | 166.88 | 229.03 | 62.15 | 0.68 |  | LPVGWEGPNVHIACIIA | 13.46 | 58.19 | 44.73 | 0.45 |
|  | VLMTGLIGLALLFLFSFY | 257.40 | 232.60 | 24.80 | 0.81 |  | LAAACAVGLASSFGA | 205.97 | 44.00 | 161.97 | 0.18 |
|  | WWWVVHLWVEGVWELIMGAIL | 252.82 | 14.29 | 121.47 | 1.09 |  | LGGVLYSIETIA | 284.24 | 1.49 | 77.25 | 0.27 |
|  | YVIIAMALISGIIGTGHH | 95.87 | 68.74 | 27.13 | 0.62 |  | QAFWKGVLSALSGAIVYEL | 334.61 | 330.83 | 3.78 | 1.04 |
|  | LGSVFSALEPLPFFAMVLF | 261.20 | 267.28 | 6.08 | 1.23 |  | LLYAILGALMGVLGALFIRCVRSIYELR | 64.34 | 47.57 | 16.76 | 2.68 |
|  | AMGTTVMAFLGAGVWGFMHTL | 137.25 | 202.32 | 65.07 | 0.97 |  | NRYFLVGVVALFASAL | 279.85 | 301.15 | 21.30 | 0.68 |
|  | LTAAHGHMAFYGAYAMIVMTII | 327.98 | 183.37 | 144.61 | 0.19 |  | IIKFILVALSIGLP | 82.67 | 47.68 | 34.98 | 0.32 |
|  | WGFWLMTVAMVFITLFLSAA | 356.32 | 331.22 | 25.10 | 2.49 |  | PAGVFVPSFLIGAGFG | 32.30 | 140.15 | 107.85 | 0.20 |
|  | FYWLREGAGVVFLIGLVAYLL | 230.31 | 201.59 | 28.72 | 2.05 |  | LVPVLISVLLAVIVGNAFN | 124.93 | 94.86 | 30.07 | 0.63 |
| **3O7P:A** | LLCSLFFLWAVANNLNDILL | 329.62 | 332.29 | 2.68 | 0.81 | **3P4W:A** | PNIILPMLFILFISWTAFWS | 32.18 | 34.29 | 2.11 | 1.48 |
| (48.95) | QSAFYFGYFIIPIPAGILM | 341.78 | 180.69 | 161.09 | 1.34 | (33.15) | EANVTLVVSTLIAHIAFNI | 21.58 | 276.40 | 105.18 | 0.23 |
|  | KAGIITGLFLYALGAALFW | 86.35 | 72.25 | 14.10 | 1.72 |  | GAIIFMIYLFYFVAVIEVTV | 74.72 | 80.52 | 5.79 | 1.15 |
|  | FLVGLFIIAAGLGCLETAANPFV | 236.05 | 206.76 | 29.29 | 0.81 |  | SRIAFPVVFLLANIILAFL | 241.72 | 261.24 | 19.52 | 1.18 |
|  | NLAQTFASFGAIIAVVFGQ | 105.72 | 96.04 | 9.68 | 1.12 | **3P5N:A** | ISMLSAIAFVLTFIKF | 164.41 | 155.27 | 9.14 | 0.64 |
|  | TPYMIIVAIVLLVALLIML | 342.72 | 2.22 | 19.50 | 1.66 | (32.39) | TLDFSDVPSLLATF | 204.22 | 260.83 | 56.61 | 0.66 |
|  | RWAVLAQFCYVGAQTACWSYL | 89.93 | 52.61 | 37.32 | 0.59 |  | VAGIIVALVKNLLNYLFSM | 35.45 | 6.67 | 28.77 | 1.59 |
|  | LTGTMVCFFIGRFTGTWLI | 233.31 | 162.89 | 70.42 | 1.82 |  | VGPFANFLAGASFLLTAYAIY | 350.62 | 311.02 | 39.59 | 0.90 |
|  | KVLAAYALIAMALCLISAFA | 27.54 | 12.88 | 14.66 | 2.39 |  | SLITGLIIATIVMTIVLSILN | 293.57 | 333.56 | 39.99 | 1.59 |
|  | GLIALTLCSAFMSIQYPTIFSLG | 291.01 | 198.53 | 92.48 | 0.33 |  | PFNIIKGIVISIVFILLYRR | 351.02 | 11.28 | 20.25 | 1.75 |
|  | YGSSFIVMTIIGGGIVTPVM | 30.73 | 327.81 | 62.92 | 0.86 | **3PJZ:A** | IRIVGLLLALFSVTMLAPALVALLY | 88.28 | 130.23 | 41.96 | 2.25 |
|  | TAELIPALCFAVIFIFARF | 30.97 | 317.74 | 73.23 | 1.80 | (49.93) | VPFVTTFFVLLFCGAMCWFPNR | 348.12 | 4.68 | 16.56 | 2.36 |
| **3OE6:A** | KIFLPTIYSIIFLTGIVGNGLVIL | 104.67 | 160.56 | 55.90 | 1.55 |  | FLIVVLFWTVLGSAGSLPFLIA | 105.82 | 170.62 | 64.80 | 1.08 |
| (48.31) | RLHLSVADLLFVITLPFWAVDA | 107.98 | 121.73 | 13.75 | 0.37 |  | AILFYRQFLQWFGGMGIIVLAVAI | 2.56 | 295.47 | 67.09 | 0.64 |
|  | GNFLCKAVHVIYTVNLYSSVWILAF | 213.02 | 200.40 | 12.62 | 0.96 |  | KALWYIYLSLTIACAVAFWLAGMT | 74.58 | 54.16 | 20.43 | 2.37 |
|  | YVGVWIPALLLTIPDFIFAN | 73.68 | 139.96 | 66.28 | 1.10 |  | YAINLITVVFLLISACNFTLHF | 53.21 | 68.88 | 15.66 | 1.61 |
|  | WVVVFQFQHIMVGLILPGIVILS | 277.20 | 326.77 | 49.57 | 1.48 |  | EFRAFIFIQVLLFLVCFLLLLK | 222.64 | 266.06 | 43.42 | 1.52 |
|  | ILILAFFACWLPYYIGISIDSFILL | 19.48 | 301.42 | 78.06 | 1.16 |  | YDAFDQALFQTVSISTT | 270.54 | 330.39 | 59.85 | 0.86 |
|  | HKWISITEALAFFHCCLNPILYA | 210.18 | 148.20 | 61.98 | 1.13 |  | LFLPVLLLFSSFIGGCAG | 264.03 | 62.14 | 158.11 | 0.51 |
|  |  |  |  |  |  |  | TGGGMKVIRILLLTL | 189.76 | 241.13 | 51.38 | 0.51 |
|  |  |  |  |  |  |  | WGFFSAYALVFVVCMLGLIATGMD | 63.32 | 69.40 | 6.08 | 1.03 |
|  |  |  |  |  |  |  | KAKWVLIVSMLFGRLEIFTLL | 14.96 | 321.12 | 53.84 | 1.37 |
|  |  |  |  |  |  |  |  |  |  |  |  |
|  |  |  |  |  |  |  |  |  |  |  |  |
|  |  |  |  |  |  |  |  |  |  |  |  |
