# Supplementary figures and images for: Lipid exposure prediction enhances the inference of rotational angles of transmembrane helices
Source: BMC Bioinformatics. 2013 Oct 11;14:304. doi: 10.1186/1471-2105-14-304 (PMC3854514; doi:10.1186/1471-2105-14-304)

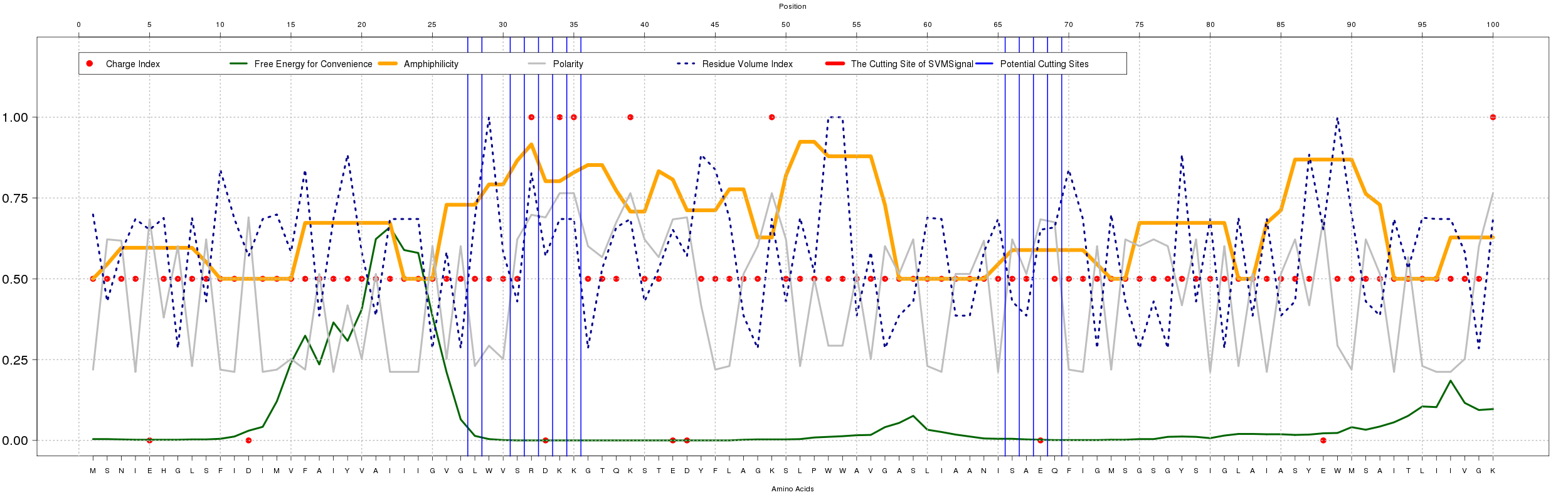

Supplement: Additional file 3: Dataset S2 — Signal peptide and topology prediction results of the independent test set from SVMSignal, TOPCONS and MemBrain. [file 1471-2105-14-304-S3.zip › S_Dataset_2_web_servers_prediction/SVMSignal/images/1.png]

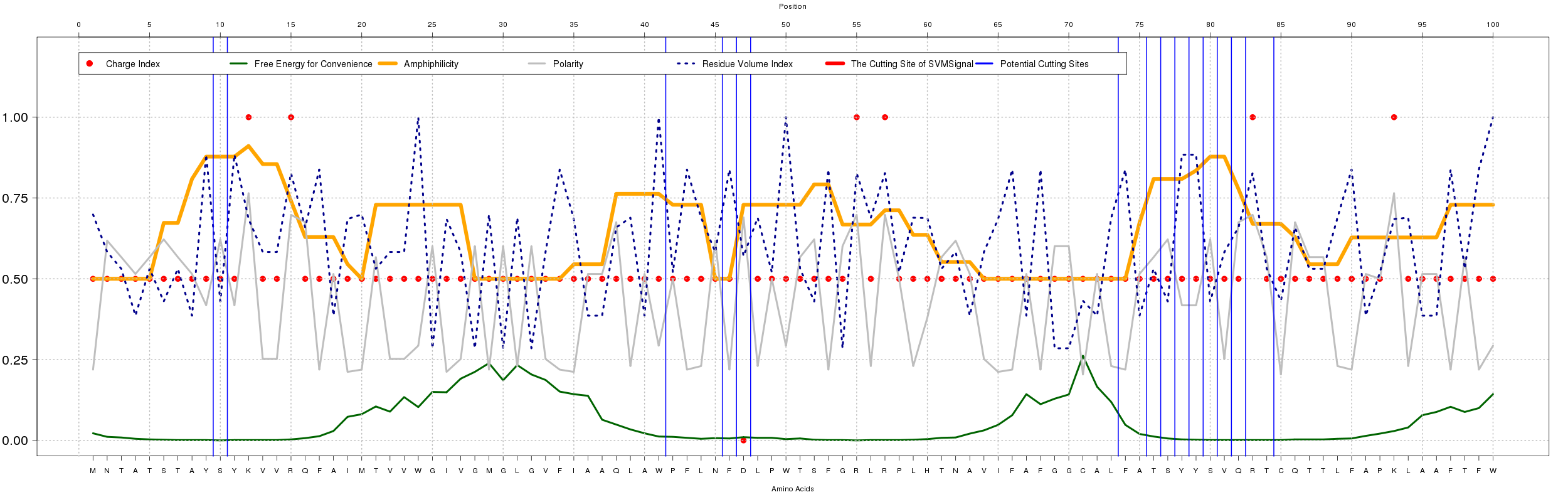

Supplement: Additional file 3: Dataset S2 — Signal peptide and topology prediction results of the independent test set from SVMSignal, TOPCONS and MemBrain. [file 1471-2105-14-304-S3.zip › S_Dataset_2_web_servers_prediction/SVMSignal/images/10.png]

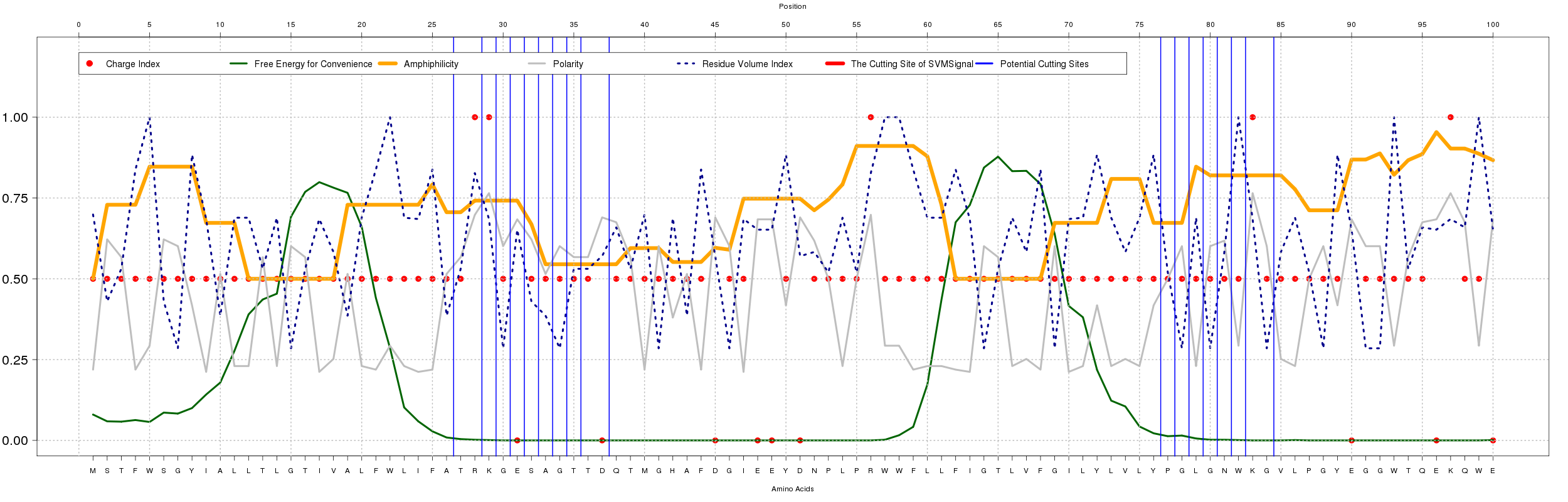

Supplement: Additional file 3: Dataset S2 — Signal peptide and topology prediction results of the independent test set from SVMSignal, TOPCONS and MemBrain. [file 1471-2105-14-304-S3.zip › S_Dataset_2_web_servers_prediction/SVMSignal/images/11.png]

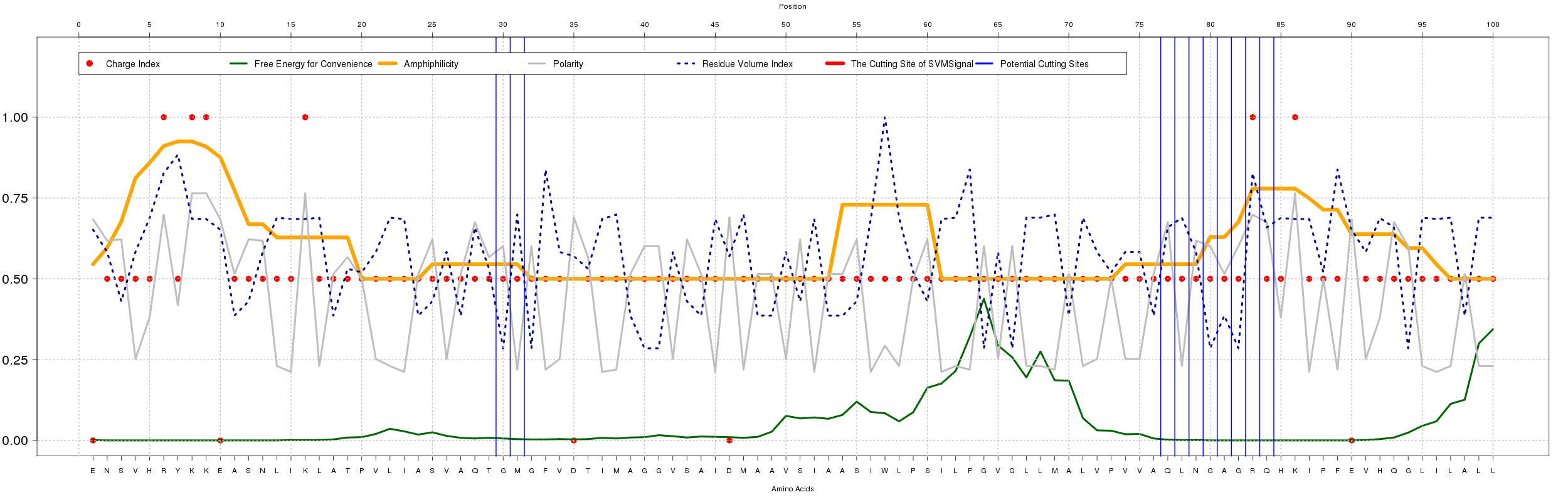

Supplement: Additional file 3: Dataset S2 — Signal peptide and topology prediction results of the independent test set from SVMSignal, TOPCONS and MemBrain. [file 1471-2105-14-304-S3.zip › S_Dataset_2_web_servers_prediction/SVMSignal/images/12.png]

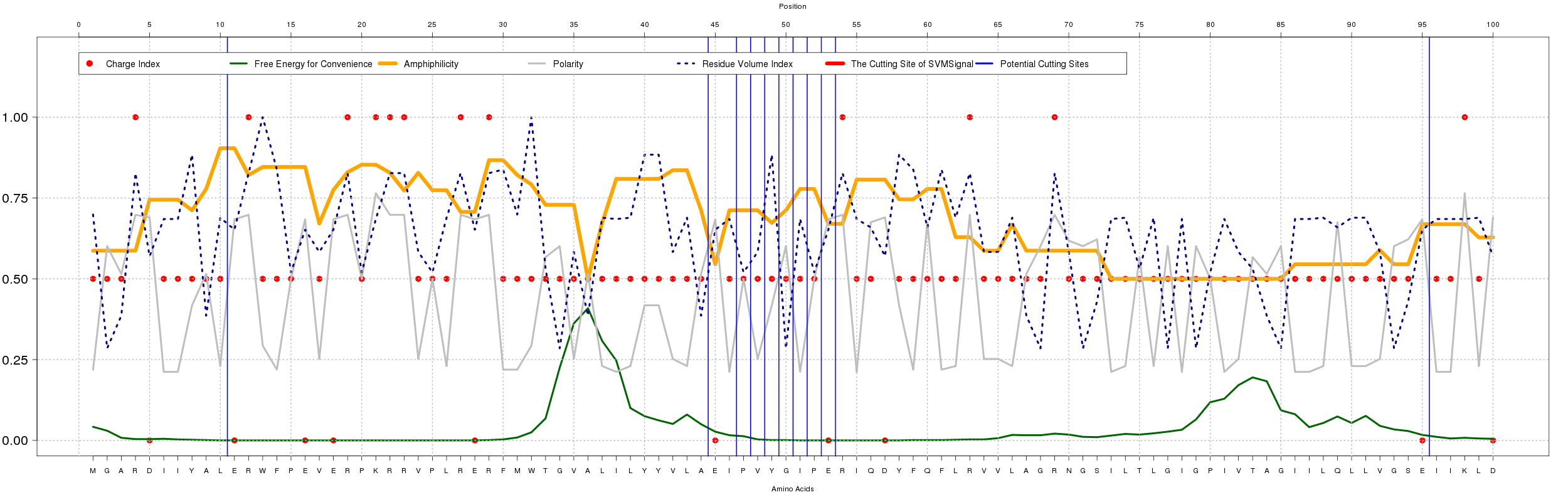

Supplement: Additional file 3: Dataset S2 — Signal peptide and topology prediction results of the independent test set from SVMSignal, TOPCONS and MemBrain. [file 1471-2105-14-304-S3.zip › S_Dataset_2_web_servers_prediction/SVMSignal/images/13.png]

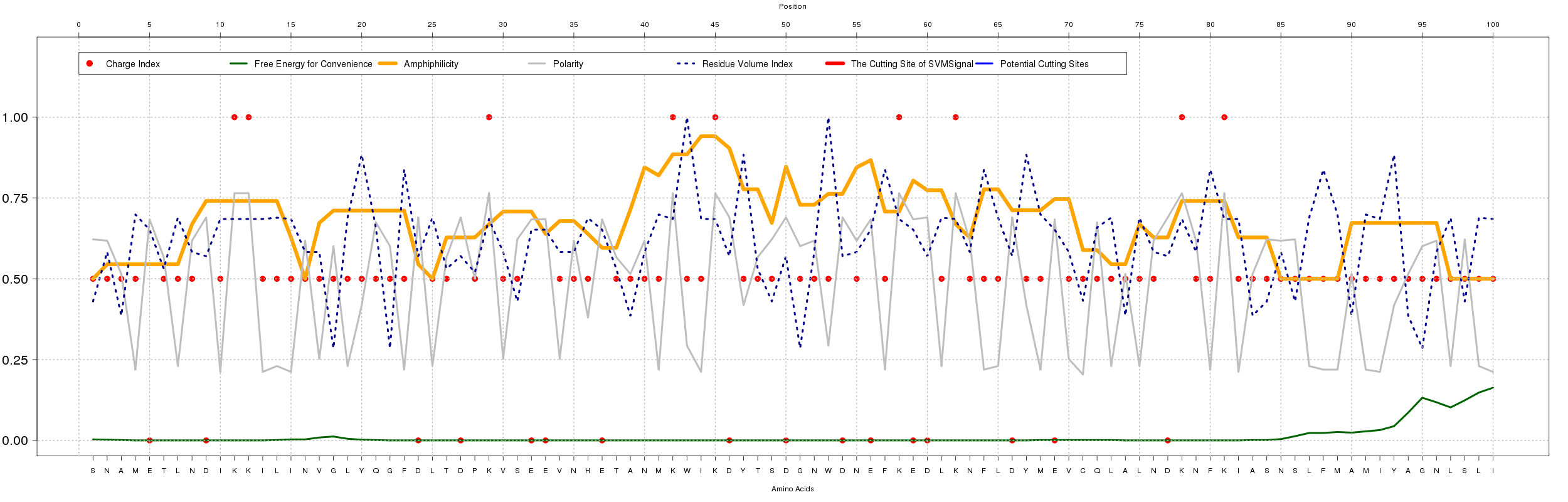

Supplement: Additional file 3: Dataset S2 — Signal peptide and topology prediction results of the independent test set from SVMSignal, TOPCONS and MemBrain. [file 1471-2105-14-304-S3.zip › S_Dataset_2_web_servers_prediction/SVMSignal/images/14.png]

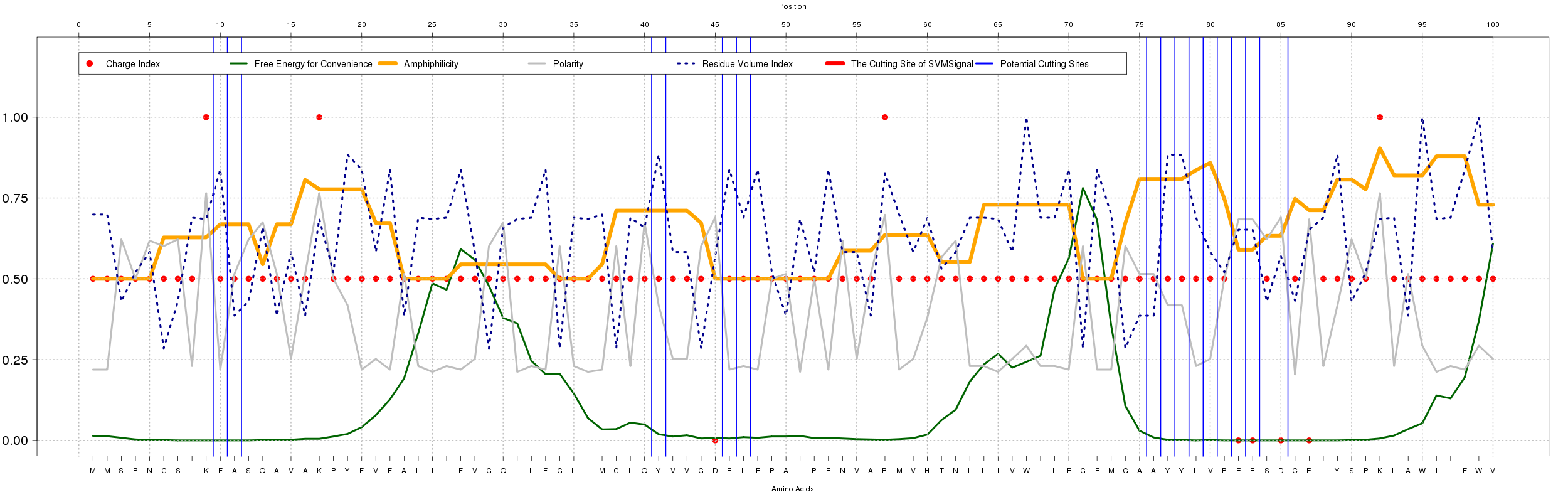

Supplement: Additional file 3: Dataset S2 — Signal peptide and topology prediction results of the independent test set from SVMSignal, TOPCONS and MemBrain. [file 1471-2105-14-304-S3.zip › S_Dataset_2_web_servers_prediction/SVMSignal/images/15.png]

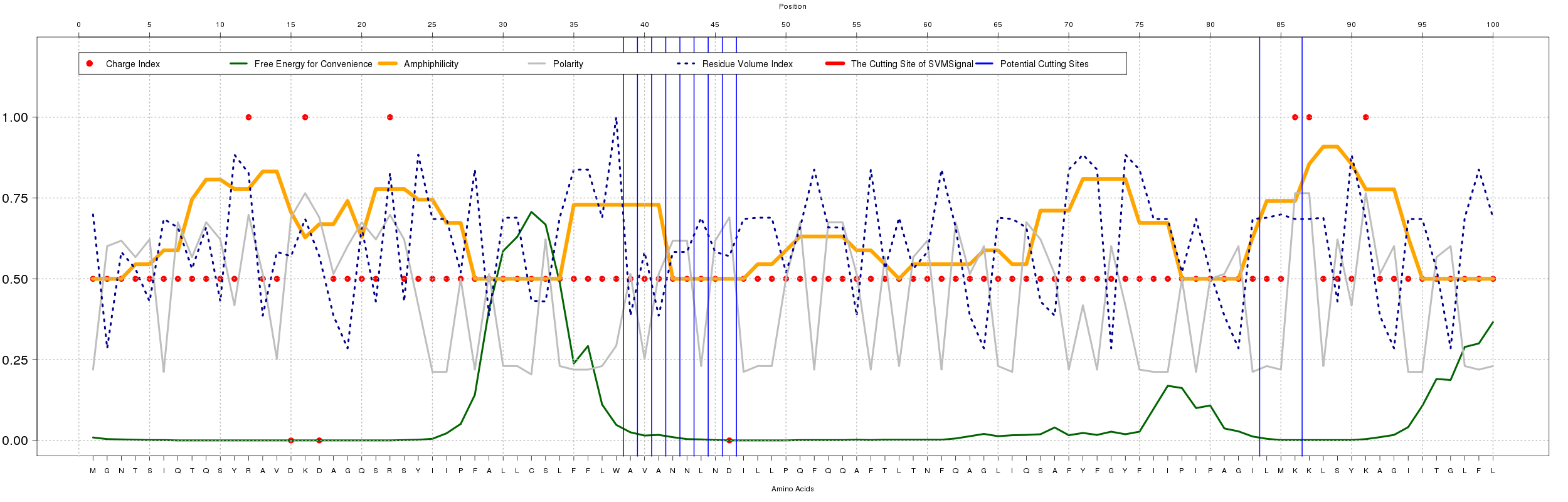

Supplement: Additional file 3: Dataset S2 — Signal peptide and topology prediction results of the independent test set from SVMSignal, TOPCONS and MemBrain. [file 1471-2105-14-304-S3.zip › S_Dataset_2_web_servers_prediction/SVMSignal/images/16.png]

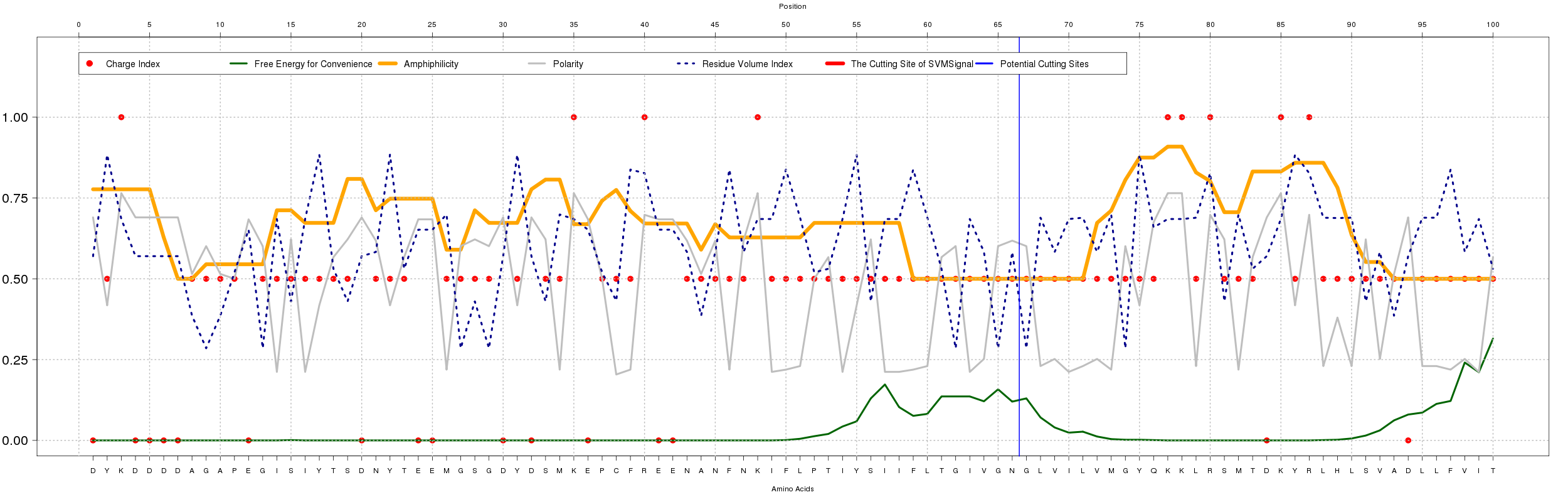

Supplement: Additional file 3: Dataset S2 — Signal peptide and topology prediction results of the independent test set from SVMSignal, TOPCONS and MemBrain. [file 1471-2105-14-304-S3.zip › S_Dataset_2_web_servers_prediction/SVMSignal/images/17.png]

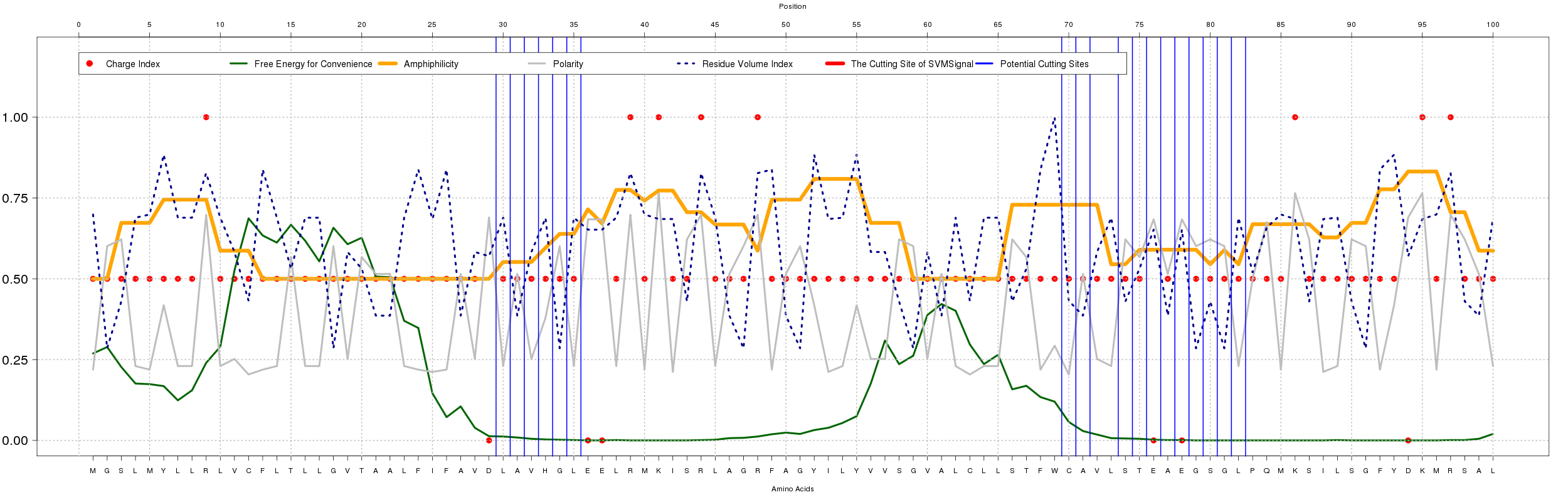

Supplement: Additional file 3: Dataset S2 — Signal peptide and topology prediction results of the independent test set from SVMSignal, TOPCONS and MemBrain. [file 1471-2105-14-304-S3.zip › S_Dataset_2_web_servers_prediction/SVMSignal/images/18.png]

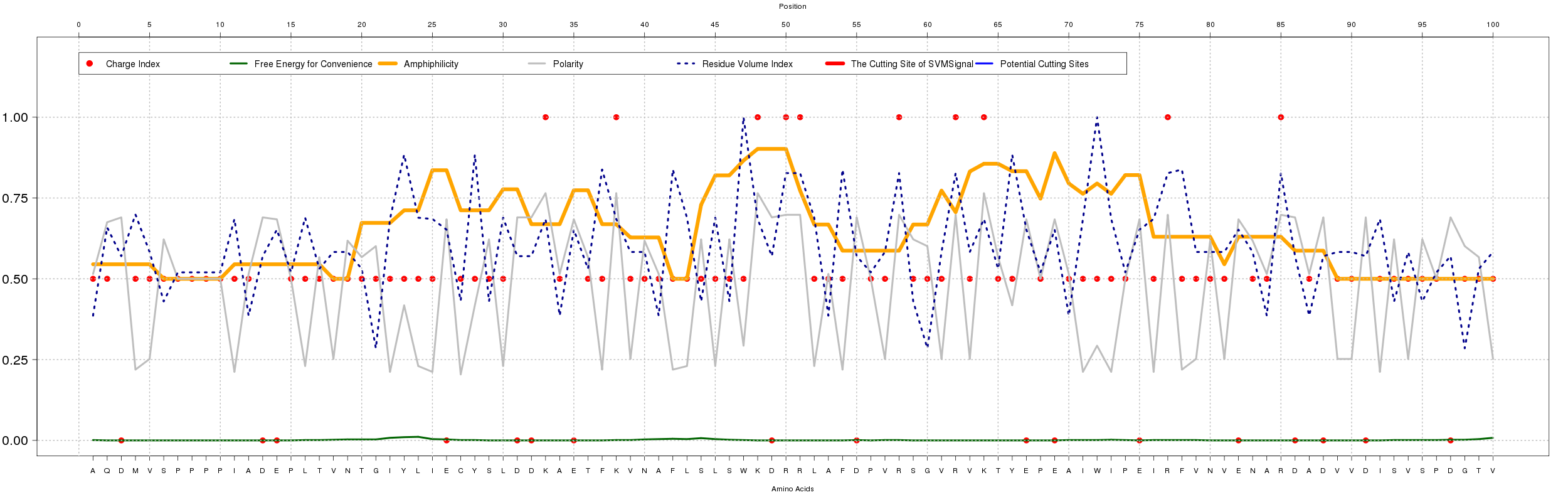

Supplement: Additional file 3: Dataset S2 — Signal peptide and topology prediction results of the independent test set from SVMSignal, TOPCONS and MemBrain. [file 1471-2105-14-304-S3.zip › S_Dataset_2_web_servers_prediction/SVMSignal/images/19.png]

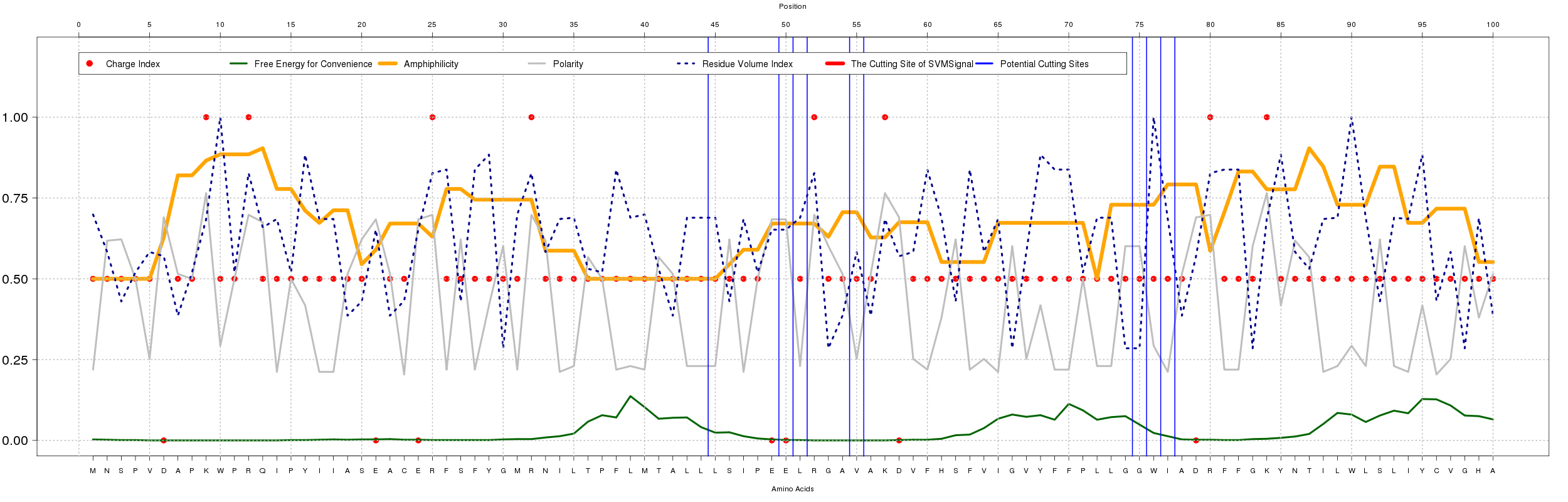

Supplement: Additional file 3: Dataset S2 — Signal peptide and topology prediction results of the independent test set from SVMSignal, TOPCONS and MemBrain. [file 1471-2105-14-304-S3.zip › S_Dataset_2_web_servers_prediction/SVMSignal/images/2.png]

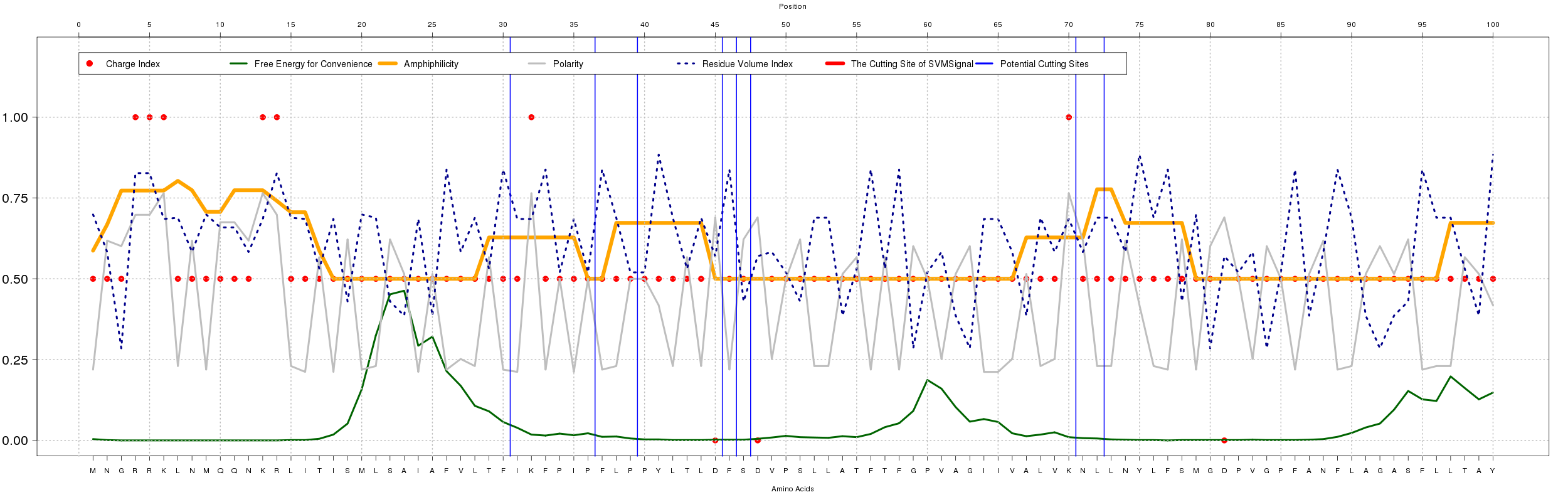

Supplement: Additional file 3: Dataset S2 — Signal peptide and topology prediction results of the independent test set from SVMSignal, TOPCONS and MemBrain. [file 1471-2105-14-304-S3.zip › S_Dataset_2_web_servers_prediction/SVMSignal/images/20.png]

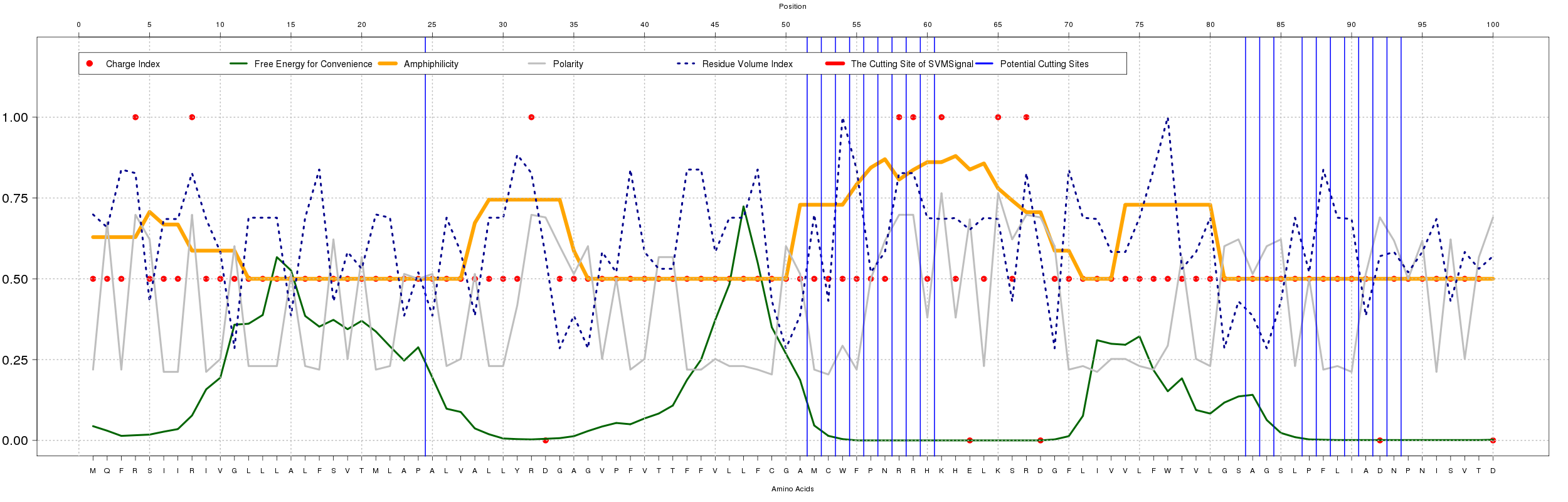

Supplement: Additional file 3: Dataset S2 — Signal peptide and topology prediction results of the independent test set from SVMSignal, TOPCONS and MemBrain. [file 1471-2105-14-304-S3.zip › S_Dataset_2_web_servers_prediction/SVMSignal/images/21.png]

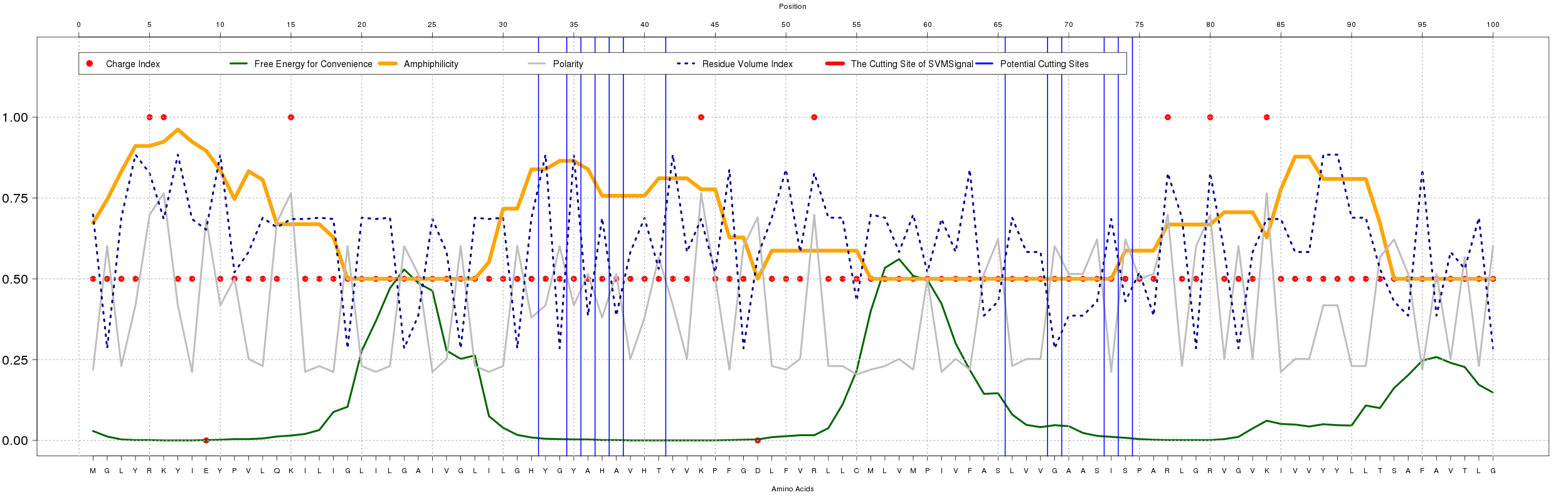

Supplement: Additional file 3: Dataset S2 — Signal peptide and topology prediction results of the independent test set from SVMSignal, TOPCONS and MemBrain. [file 1471-2105-14-304-S3.zip › S_Dataset_2_web_servers_prediction/SVMSignal/images/3.png]

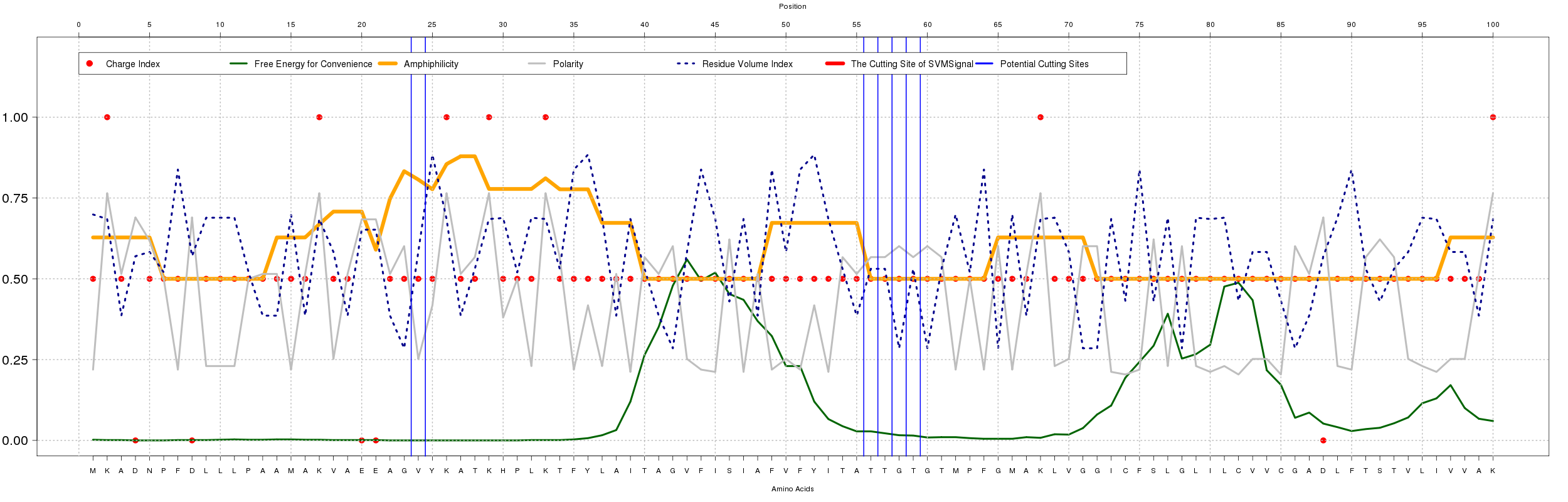

Supplement: Additional file 3: Dataset S2 — Signal peptide and topology prediction results of the independent test set from SVMSignal, TOPCONS and MemBrain. [file 1471-2105-14-304-S3.zip › S_Dataset_2_web_servers_prediction/SVMSignal/images/4.png]

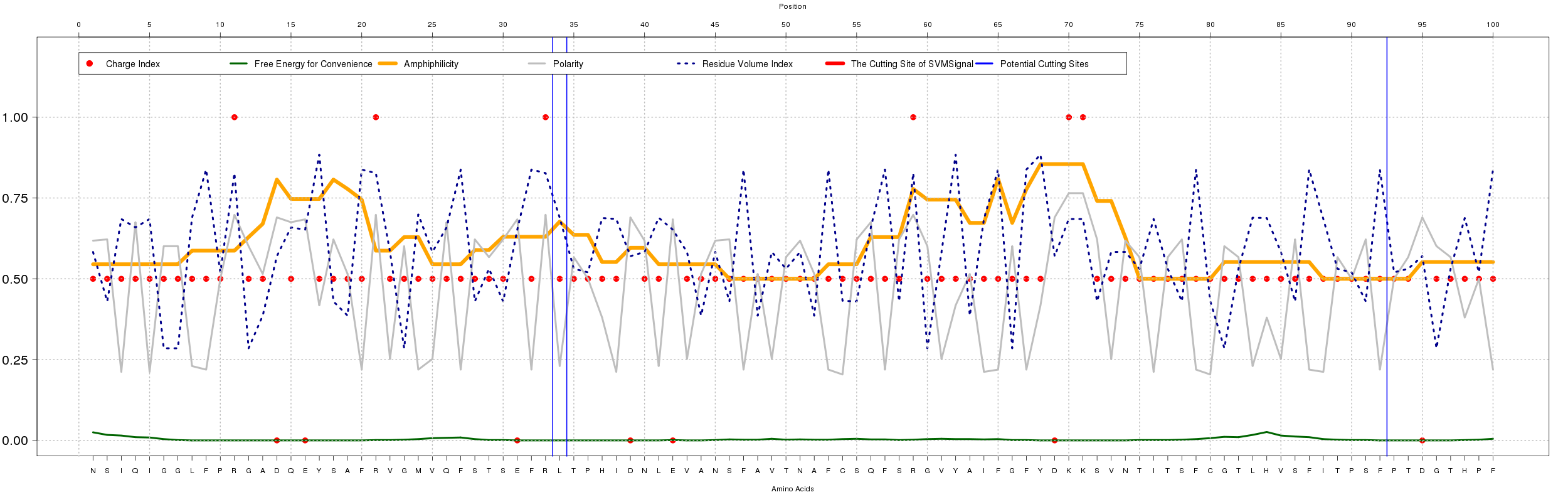

Supplement: Additional file 3: Dataset S2 — Signal peptide and topology prediction results of the independent test set from SVMSignal, TOPCONS and MemBrain. [file 1471-2105-14-304-S3.zip › S_Dataset_2_web_servers_prediction/SVMSignal/images/5.png]

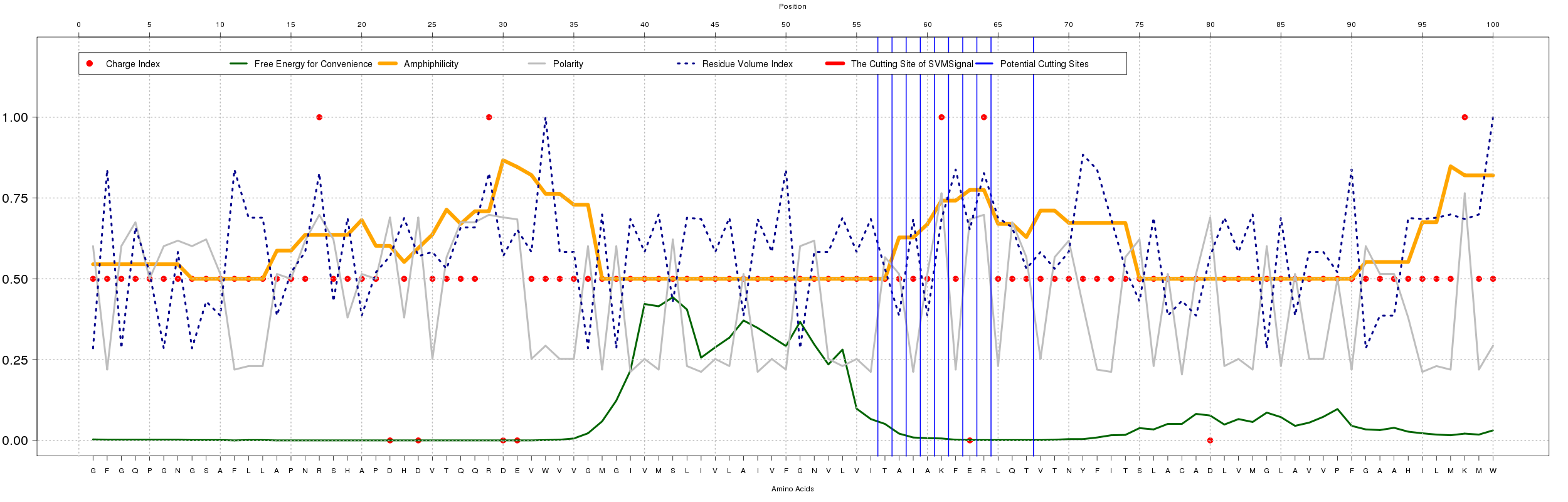

Supplement: Additional file 3: Dataset S2 — Signal peptide and topology prediction results of the independent test set from SVMSignal, TOPCONS and MemBrain. [file 1471-2105-14-304-S3.zip › S_Dataset_2_web_servers_prediction/SVMSignal/images/6.png]

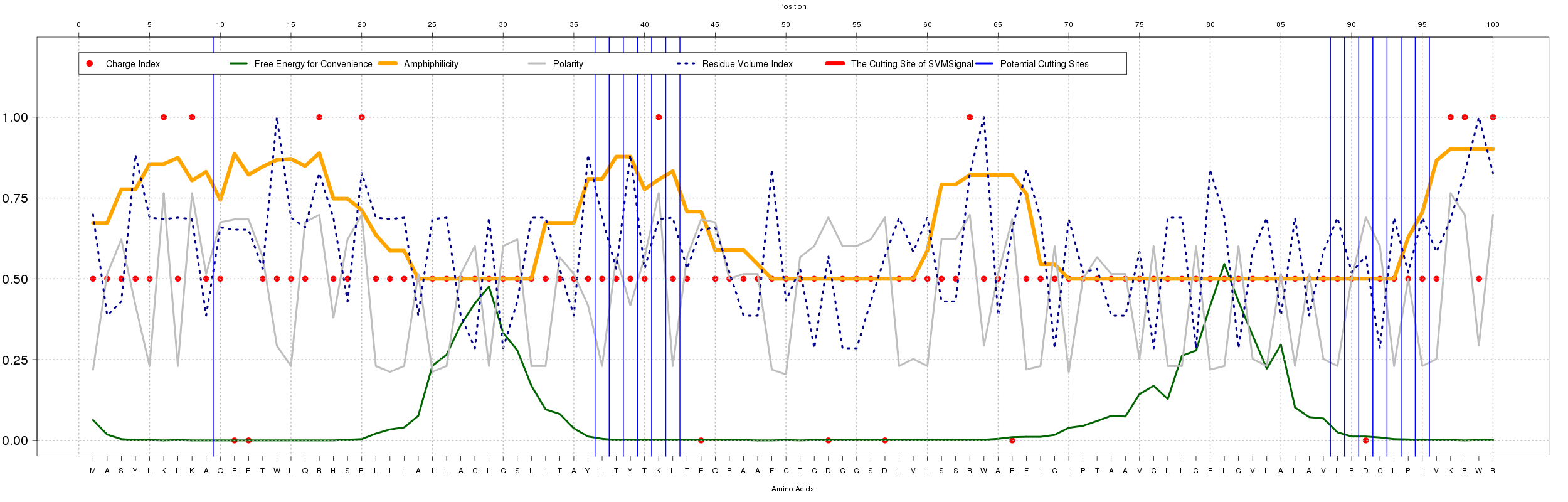

Supplement: Additional file 3: Dataset S2 — Signal peptide and topology prediction results of the independent test set from SVMSignal, TOPCONS and MemBrain. [file 1471-2105-14-304-S3.zip › S_Dataset_2_web_servers_prediction/SVMSignal/images/7.png]

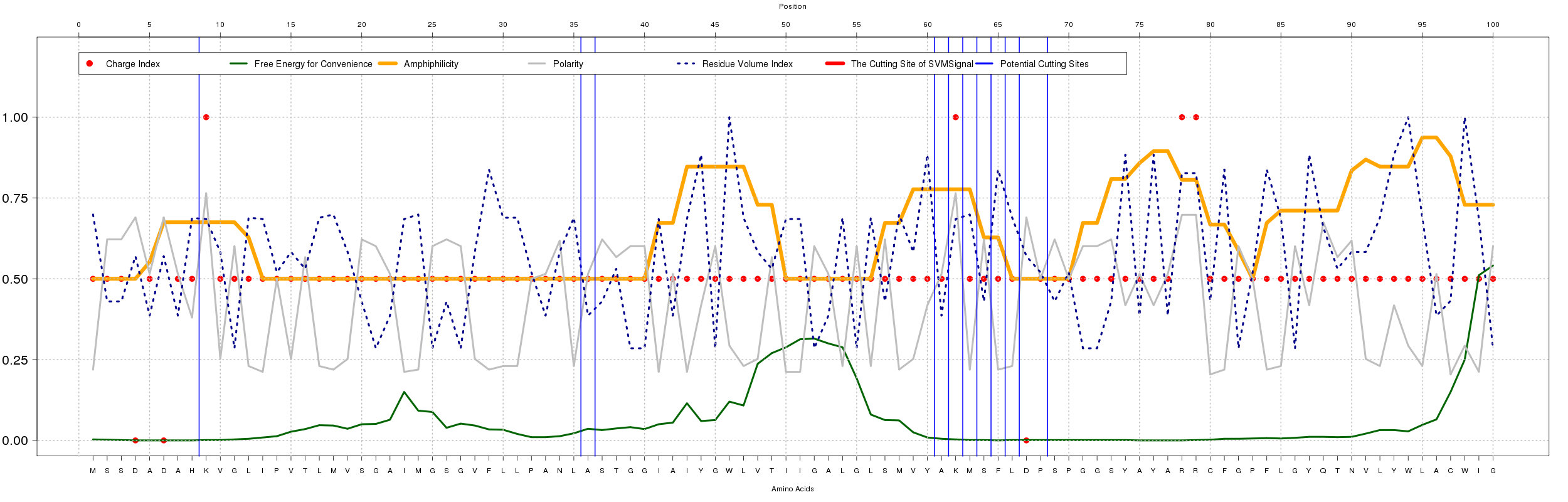

Supplement: Additional file 3: Dataset S2 — Signal peptide and topology prediction results of the independent test set from SVMSignal, TOPCONS and MemBrain. [file 1471-2105-14-304-S3.zip › S_Dataset_2_web_servers_prediction/SVMSignal/images/8.png]

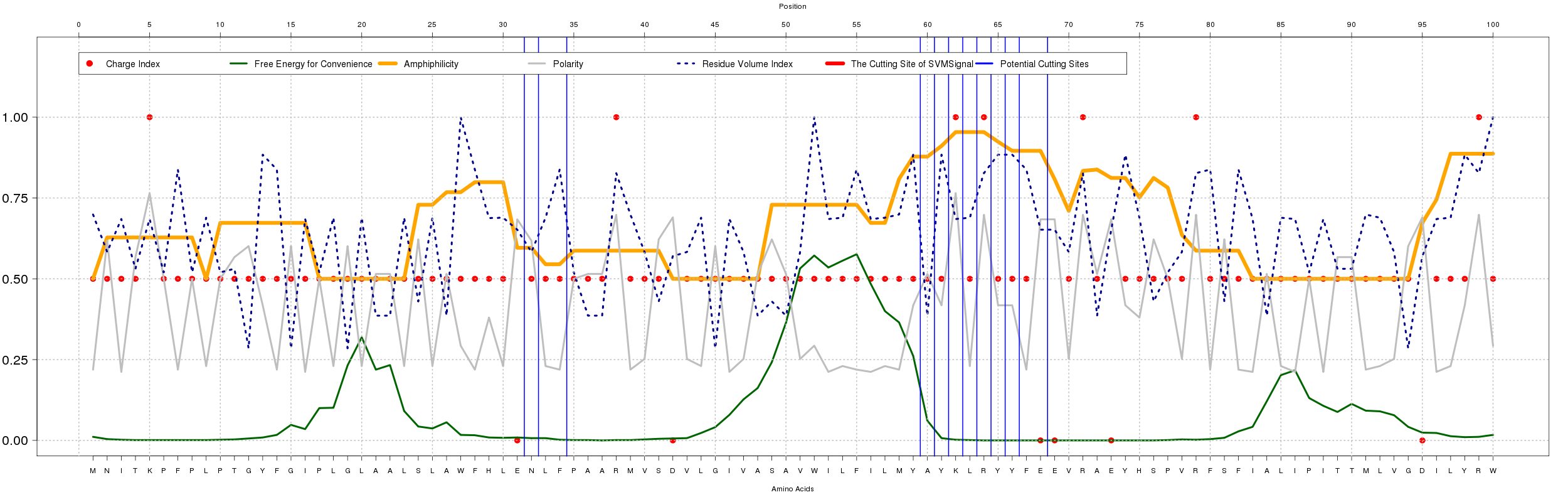

Supplement: Additional file 3: Dataset S2 — Signal peptide and topology prediction results of the independent test set from SVMSignal, TOPCONS and MemBrain. [file 1471-2105-14-304-S3.zip › S_Dataset_2_web_servers_prediction/SVMSignal/images/9.png]
