# Supplementary material for: Lipid exposure prediction enhances the inference of rotational angles of transmembrane helices
Source: BMC Bioinformatics. 2013 Oct 11;14:304. doi: 10.1186/1471-2105-14-304 (PMC3854514; doi:10.1186/1471-2105-14-304)
Supplement: Additional file 4: Table S2 — Sequences, observed angles defined by residues of predicted topology (by TOPCONS), predicted angles, moment lengths and MAAE on the 155 TMHs of the independent test set. [file 1471-2105-14-304-S4.docx]

Table S2. Sequences, observed angles defined by residues of predicted topology (by TOPCONS), predicted angles, moment lengths and MAAE on the 155 TMHs of the independent test set.

| PDB:Chain |  | TOPCONS predicted topology |  |  |  |  |
| --- | --- | --- | --- | --- | --- | --- |
| (MAAE) | PDBTM TM helix sequence | predicted TM helix sequence | observed angle | predicted angle | angular error |  |
| **2XQ2:A** | AVGASLIAANISAEQF | LPWWAVGASLIAANISAEQFI | 275.09 | 271.92 | 3.17 | 0.47 |
| (50.34) | ASYEWMSAITLIIV | YEWMSAITLIIVGKYFLPIFI | 218.22 | 208.78 | 9.44 | 0.69 |
|  | LAVFWISLYIFVNLTSVLYLG | ILAVFWISLYIFVNLTSVLYL | 293.78 | 307.30 | 13.52 | 0.50 |
|  | VWTDVIQVFFLVLGG | VIQVFFLVLGGFMTTYMAVSF | 90.41 | 66.71 | 23.70 | 2.23 |
|  | AVLIGGLWVANLYYWGFNQ | LPGIAVLIGGLWVANLYYWGF | 206.63 | 35.69 | 170.94 | 0.35 |
|  | VFAAFLALIVPFLV | AFLALIVPFLVVLPGIAAYVI | 130.22 | 110.17 | 20.05 | 1.47 |
|  | ALAAAIVSSLASMLNSTA | VKGVVFAALAAAIVSSLASML | 230.19 | 109.97 | 120.22 | 0.98 |
|  | RTAAVVALIIAALIA | VNVGRTAAVVALIIAALIAPM | 158.03 | 151.48 | 6.55 | 2.49 |
|  | QYIQEYTGLVSPGILA | QEYTGLVSPGILAVFLLGLFW | 152.04 | 48.20 | 103.84 | 0.35 |
|  | VVASIPFALFLKFM | GAIIGVVASIPFALFLKFMPL | 299.65 | 283.57 | 16.08 | 1.46 |
|  | FMDQMLYTLLFTMVV | QMLYTLLFTMVVIAFTSLSTS | 303.88 | 258.31 | 45.57 | 1.57 |
|  | AAYGIMIVLAVLYT | IAAYGIMIVLAVLYTLFWVNA | 343.44 | 347.31 | 3.87 | 1.15 |
|  | GVMAGVIGTILLISYGIK | LIIFGVMAGVIGTILLISYGI | 265.54 | 23.05 | 117.51 | 0.16 |
| **2XUT:A** | ASEACERFSFYGMRNILTPFL | FSFYGMRNILTPFLMTALLLS | 332.39 | 356.70 | 24.31 | 0.62 |
| (38.48) | VAKDVFHSFVIGVYFFPLLG | DVFHSFVIGVYFFPLLGGWIA | 46.42 | 9.77 | 36.65 | 1.01 |
|  | TILWLSLIYCVGHAFLAIFE | YNTILWLSLIYCVGHAFLAIF | 85.88 | 93.39 | 7.51 | 1.88 |
|  | GFYTGLFLIALGSGGIKP | HSVQGFYTGLFLIALGSGGIK | 268.54 | 320.42 | 51.88 | 0.88 |
|  | YFTINFGSFFASLSMPLLL | MFYFTINFGSFFASLSMPLLL | 318.76 | 9.64 | 50.88 | 1.34 |
|  | VAFGIPGVLMFVATVFFWL | AVAFGIPGVLMFVATVFFWLG | 67.34 | 74.70 | 7.36 | 1.44 |
|  | IGGVSAAYALVNIPTL | NIGLVLALIGGVSAAYALVNI | 339.38 | 24.93 | 45.55 | 0.44 |
|  | IVAGLCCAMVLVMGFVG | TLGIVAGLCCAMVLVMGFVGA | 34.22 | 20.20 | 14.02 | 0.60 |
|  | ALVTPFWSLFDQKASTWILQA | VLRILVLFALVTPFWSLFDQK | 234.04 | 100.54 | 133.50 | 0.52 |
|  | GAGIAITGLSWIVVGTIQ | MGAGIAITGLSWIVVGTIQLM | 203.95 | 169.40 | 34.55 | 1.95 |
|  | SIFWQILPYALLTFGEVLVS | IFWQILPYALLTFGEVLVSAT | 125.97 | 94.43 | 31.54 | 0.65 |
|  | FWTLSVTVGNLWVLLANVSV | TIMSFWTLSVTVGNLWVLLAN | 35.48 | 11.51 | 23.97 | 0.94 |
| **3KBC:A** | QKILIGLILGAIVGLI | VLQKILIGLILGAIVGLILGH | 258.38 | 231.32 | 27.06 | 1.22 |
| (64.97) | VKPFGDLFVRLLCMLVMPIVFASL | FVRLLCMLVMPIVFASLVVGA | 244.98 | 236.02 | 8.96 | 0.64 |
|  | AFAVTLGIIMARLFNPG | VYYLLTSAFAVTLGIIMARLF | 43.48 | 54.70 | 11.22 | 1.46 |
|  | QVLPTIFFAIIL | QVLPTIFFAIILGIAITYLMN | 284.42 | 230.22 | 54.20 | 0.72 |
|  | VMQYAPIGVFALIAYVM | GVMQYAPIGVFALIAYVMAEQ | 247.58 | 44.30 | 156.72 | 0.75 |
|  | VGELAKVTAAVYVGLTLQ | AVYVGLTLQILLVYFVLLKIY | 282.99 | 265.68 | 17.31 | 1.10 |
|  | GTALYQGVATFFIA | GTALYQGVATFFIANALGSHL | 263.27 | 10.07 | 106.80 | 0.14 |
|  | GAGAIMLCMVLHSVGLPLT | TAGVPGAGAIMLCMVLHSVGL | 282.97 | 145.47 | 137.50 | 0.44 |

| PDB:Chain |  | TOPCONS predicted topology |  |  |  |  |
| --- | --- | --- | --- | --- | --- | --- |
| (MAAE) | PDBTM TM helix sequence | predicted TM helix sequence | observed angle | predicted angle | angular error |  |
| **3KCU:A** | YLAITAGVFISIAFVFYITATTGT | FYLAITAGVFISIAFVFYITA | 104.96 | 61.44 | 43.52 | 0.76 |
| (39.26) | PFGMAKLVGGICFSLGLILCV | LVGGICFSLGLILCVVCGADL | 115.28 | 42.20 | 73.08 | 0.69 |
|  | LNVYFGNLVGALLFVLLMWLSGEYMTA | WLNVYFGNLVGALLFVLLMWL | 172.15 | 155.74 | 16.41 | 2.34 |
|  | GLNVLQTADHKVHHTFIEAVCLGILANLMVCLAV | IEAVCLGILANLMVCLAVWMS | 45.68 | 58.17 | 12.49 | 1.05 |
|  | FIMVLPVAMFVA | AFIMVLPVAMFVASGFEHSIA | 163.45 | 91.65 | 71.80 | 0.68 |
|  | LTVMNFITDNLIPVTIGNIIGGGLL | VTIGNIIGGGLLVGLTYWVIY | 232.52 | 250.79 | 18.27 | 0.66 |
| **3KG2:A** | AYEIWMCIVFAYIGVSVVLF | EIWMCIVFAYIGVSVVLFLVS | 8.96 | 314.45 | 54.51 | 1.54 |
| (35.69) | SGRIVGGVWWFFTLIIISSYT | VGGVWWFFTLIIISSYTANLA | 235.26 | 200.01 | 35.25 | 0.92 |
|  | VAGVFYILVGGLGLAMLVAL | FYILVGGLGLAMLVALIEFCY | 67.34 | 84.66 | 17.32 | 1.56 |
| **3KP9:A** | ILAILAGLGSLLTAYLTYT | HSRLILAILAGLGSLLTAYLT | 240.31 | 328.24 | 87.93 | 2.15 |
| (52.97) | AEFLGIPTAAVGLLGFLGVLALAVL | LGIPTAAVGLLGFLGVLALAV | 266.47 | 270.33 | 3.86 | 0.85 |
|  | LFGLVSAMTAFEMYMLYLMVA | LFGLVSAMTAFEMYMLYLMVA | 155.56 | 191.01 | 35.45 | 1.83 |
|  | CMYCTTAIILVAGLGLVTV | FCMYCTTAIILVAGLGLVTVL | 77.50 | 215.10 | 137.60 | 1.58 |
|  | FSYILVAFLTLVTTIGVYANQ | LAFSYILVAFLTLVTTIGVYA | 198.33 | 198.36 | 0.03 | 0.67 |
| **3L1L:A** | GLIPVTLMVSGAIMGSGVFLLP | VGLIPVTLMVSGAIMGSGVFL | 241.43 | 311.39 | 69.96 | 0.37 |
| (20.75) | IYGWLVTIIGALGLSMVYAK | IAIYGWLVTIIGALGLSMVYA | 289.65 | 261.74 | 27.91 | 1.57 |
|  | LGYQTNVLYWLACWIGNIAMVVIGVGYL | VLYWLACWIGNIAMVVIGVGY | 0.74 | 347.54 | 13.20 | 0.78 |
|  | LTITCVVVLWIFVLLNIVGP | PWVLTITCVVVLWIFVLLNIV | 170.47 | 188.68 | 18.22 | 1.59 |
|  | MITRVQAVATVLALIPIVGIAVFGW | VATVLALIPIVGIAVFGWFWF | 28.16 | 336.63 | 51.53 | 2.11 |
|  | PIATIGGVLIAAVCYVLSTTA | GGVLIAAVCYVLSTTAIMGMI | 347.68 | 349.69 | 2.01 | 1.44 |
|  | VSFCAAAGCLGSLGGWTLLAGQTAK | AIVSFCAAAGCLGSLGGWTLL | 35.60 | 30.83 | 4.77 | 0.89 |
|  | VAGLIIVGILMTIFQLSSISPNA | VAGLIIVGILMTIFQLSSISP | 88.25 | 99.50 | 11.25 | 1.56 |
|  | FGLVSSVSVIFTLVPYLYTCAAL | FGLVSSVSVIFTLVPYLYTCA | 160.84 | 181.12 | 20.28 | 1.19 |
|  | AYLAVTTIAFLYCIWAVVGSGA | AYLAVTTIAFLYCIWAVVGSG | 270.41 | 279.40 | 8.99 | 1.75 |
|  | EVMWSFVTLMVITAMYALN | EVMWSFVTLMVITAMYALNYN | 224.20 | 224.28 | 0.08 | 1.86 |
| **3M71:A** | GYFGIPLGLAALSLAW | FPLPTGYFGIPLGLAALSLAW | 341.77 | 359.56 | 17.79 | 0.32 |
| (22.40) | SDVLGIVASAVWILFILMYAY | SDVLGIVASAVWILFILMYAY | 165.18 | 183.80 | 18.62 | 2.22 |
|  | FIALIPITTMLVGDIL | FSFIALIPITTMLVGDILYRW | 287.37 | 317.27 | 29.90 | 0.34 |
|  | AEVLIWIGTIGQLLFSTLRVS | IAEVLIWIGTIGQLLFSTLRV | 294.71 | 317.19 | 22.48 | 1.72 |
|  | SFYLPAVAANFTSA | FYLPAVAANFTSASSLALLGY | 153.77 | 199.49 | 45.72 | 0.24 |
|  | YLFFGAGMIAWIIFEPVLL | LGYLFFGAGMIAWIIFEPVLL | 282.17 | 283.84 | 1.67 | 1.54 |
|  | MGIVLAPAFVCVSAY | ATMGIVLAPAFVCVSAYLSIN | 203.05 | 222.00 | 18.95 | 1.06 |
|  | AKILWGYGFLQLFFLLRLF | TLAKILWGYGFLQLFFLLRLF | 5.74 | 349.44 | 16.30 | 1.11 |
|  | GLWAFSFGLASMANSA | IGLWAFSFGLASMANSATAFY | 286.90 | 327.80 | 40.90 | 0.17 |
|  | SIFAFVFSNVMIGLLVLMTIYKLT | VSIFAFVFSNVMIGLLVLMTI | 265.02 | 276.64 | 11.62 | 2.41 |

| PDB:Chain |  | TOPCONS predicted topology |  |  |  |  |
| --- | --- | --- | --- | --- | --- | --- |
| (MAAE) | PDBTM TM helix sequence | predicted TM helix sequence | observed angle | predicted angle | angular error |  |
| **3MK7:A** | QFAIMTVVWGIVGMGLGV | FAIMTVVWGIVGMGLGVFIAA | 229.83 | 235.34 | 5.51 | 1.47 |
| (52.44) | HTNAVIFAFGGCALFATS | TNAVIFAFGGCALFATSYYSV | 169.08 | 111.18 | 57.90 | 0.48 |
|  | LAAFTFWGWQLVILLAA | FTFWGWQLVILLAAISLPLGF | 327.49 | 307.19 | 20.30 | 1.52 |
|  | IDILITIVWVAYAVVFF | IDILITIVWVAYAVVFFGTLA | 220.71 | 239.57 | 18.87 | 1.05 |
|  | WFFGAFILTVAILHV | IYVGNWFFGAFILTVAILHVV | 23.28 | 90.81 | 67.53 | 0.12 |
|  | HNAVGFFLTAGFLGIM | WWYGHNAVGFFLTAGFLGIMY | 104.56 | 276.21 | 171.65 | 0.18 |
|  | IVHFWALITVYIWA | VYSYRLSIVHFWALITVYIWA | 169.10 | 60.66 | 108.44 | 1.47 |
|  | MSLILLAPSWGGMI | WAQSLGMVMSLILLAPSWGGM | 303.71 | 311.71 | 8.00 | 1.41 |
|  | FLVVSLAFYGMSTFEGPM | ILRFLVVSLAFYGMSTFEGPM | 190.59 | 164.11 | 26.48 | 0.62 |
|  | GHVHAGALGWVAMVSI | VHAGALGWVAMVSIGALYHLV | 159.36 | 57.49 | 101.87 | 0.60 |
|  | THFWLATIGTVLYIA | THFWLATIGTVLYIASMWVNG | 343.30 | 325.64 | 17.66 | 2.16 |
|  | RMIGGAIFFAGMLVMAY | GFVVRMIGGAIFFAGMLVMAY | 218.32 | 193.22 | 25.10 | 2.40 |
| **3MK7:C** | LTLGTIVALFWLIFA | WSGYIALLTLGTIVALFWLIF | 299.69 | 234.95 | 64.74 | 1.86 |
| (77.01) | RWWFLLFIGTLVFGI | WWFLLFIGTLVFGILYLVLYP | 8.55 | 279.28 | 89.27 | 2.01 |
| **3MKT:A** | ATPVLIASVAQTGMGFVD | LIKLATPVLIASVAQTGMGFV | 161.97 | 149.56 | 12.41 | 1.56 |
| (46.18) | WLPSILFGVGLLMALVPV | IAASIWLPSILFGVGLLMALV | 265.22 | 50.98 | 145.76 | 0.62 |
|  | QGLILALLVSVPIIAVLF | LILALLVSVPIIAVLFQTQFI | 128.03 | 135.53 | 7.51 | 1.92 |
|  | MHAVIFAVPAYLLFQALRSF | TVGYMHAVIFAVPAYLLFQAL | 233.07 | 137.20 | 95.87 | 0.77 |
|  | KPAMVIGFIGLLLNIPL | AMVIGFIGLLLNIPLNWIFVY | 209.06 | 217.87 | 8.81 | 1.89 |
|  | ATAIVYWIMLLLLLFYIV | GVATAIVYWIMLLLLLFYIVT | 135.13 | 120.79 | 14.34 | 1.73 |
|  | IRLFRLGFPVAAALFFEVTLFAVVA | LGFPVAAALFFEVTLFAVVAL | 295.64 | 223.81 | 71.83 | 1.36 |
|  | ALNFSSLVFMFPMSIGAA | VALNFSSLVFMFPMSIGAAVS | 332.75 | 57.21 | 84.46 | 0.52 |
|  | GLATACITALLTVLFREQIA | VGLMTGLATACITALLTVLFR | 300.90 | 324.07 | 23.17 | 1.42 |
|  | QLLLFAAIYQCMDAVQVVAAGS | VVALAMQLLLFAAIYQCMDAV | 204.71 | 199.94 | 4.77 | 0.75 |
|  | AIFHRTFISYWVLGLPTGYILGMT | FISYWVLGLPTGYILGMTNWL | 212.38 | 171.66 | 40.72 | 0.95 |
|  | GFWLGFIIGLSAAALML | AKGFWLGFIIGLSAAALMLGQ | 212.56 | 168.00 | 44.56 | 0.72 |
| **3MP7:A** | GVALILYYVLAEIPV | FMWTGVALILYYVLAEIPVYG | 159.94 | 86.72 | 73.22 | 1.86 |
| (51.17) | VFSVFMCFFEAAVWI | RVFSVFMCFFEAAVWILGGAF | 190.56 | 134.70 | 55.86 | 1.47 |
|  | ISLFIAAGVSQTILTRSLNP | IGSGISLFIAAGVSQTILTRS | 28.63 | 73.01 | 44.38 | 0.10 |
|  | DMLSVVATIVVFFIVVYF | DMLSVVATIVVFFIVVYFESM | 178.49 | 156.63 | 21.86 | 2.10 |
|  | NIPIILTFALYANIQLWA | FLYVSNIPIILTFALYANIQL | 304.58 | 344.16 | 39.58 | 0.78 |
|  | VRAIVYLILTVIFSLLFG | YLILTVIFSLLFGYLWVELTG | 233.97 | 230.00 | 3.97 | 1.70 |
|  | ALGTGTGILLTVGIL | LGALGTGTGILLTVGILYRFY | 315.60 | 74.94 | 119.34 | 0.14 |

| PDB:Chain |  | TOPCONS predicted topology |  |  |  |  |
| --- | --- | --- | --- | --- | --- | --- |
| (MAAE) | PDBTM TM helix sequence | predicted TM helix sequence | observed angle | predicted angle | angular error |  |
| **3NYM:A**(97.58) | IASNSLFMAMIYAGNLSLIFD | IASNSLFMAMIYAGNLSLIFD | 298.60 | 36.17 | 97.58 | 0.46 |
| **3O0R:B** | YFVFALILFVGQILFGLIMGL | YFVFALILFVGQILFGLIMGL | 227.91 | 237.47 | 9.56 | 2.08 |
| (56.21) | ARMVHTNLLIVWLLFGFMGAAY | TNLLIVWLLFGFMGAAYYLVP | 178.22 | 35.77 | 142.45 | 0.24 |
|  | LAWILFWVFAAAGVLTILGYLL | ILFWVFAAAGVLTILGYLLVP | 3.52 | 335.11 | 28.41 | 1.51 |
|  | FLEQPTISKAGIVIVALGFLFNVGM | TISKAGIVIVALGFLFNVGMT | 90.25 | 68.41 | 21.84 | 0.77 |
|  | VLMTGLIGLALLFLFSFY | ISMVLMTGLIGLALLFLFSFY | 187.07 | 174.26 | 12.81 | 0.94 |
|  | WWWVVHLWVEGVWELIMGAIL | WVEGVWELIMGAILAFVLVKI | 231.19 | 45.63 | 174.44 | 0.72 |
|  | YVIIAMALISGIIGTGHH | IEKWLYVIIAMALISGIIGTG | 198.02 | 219.12 | 21.11 | 0.78 |
|  | LGSVFSALEPLPFFAMVLF | YWLWLGSVFSALEPLPFFAMV | 260.00 | 305.21 | 45.21 | 1.05 |
|  | AMGTTVMAFLGAGVWGFMHTL | ALWAMGTTVMAFLGAGVWGFM | 58.30 | 136.16 | 77.86 | 0.98 |
|  | LTAAHGHMAFYGAYAMIVMTII | GHMAFYGAYAMIVMTIISYAM | 155.00 | 63.24 | 91.76 | 0.33 |
|  | WGFWLMTVAMVFITLFLSAA | FWLMTVAMVFITLFLSAAGVL | 159.65 | 135.11 | 24.54 | 2.53 |
|  | FYWLREGAGVVFLIGLVAYLL | FYWLREGAGVVFLIGLVAYLL | 226.19 | 201.59 | 24.60 | 2.05 |
| **3O7P:A** | LLCSLFFLWAVANNLNDILL | YIIPFALLCSLFFLWAVANNL | 222.02 | 194.84 | 27.18 | 1.19 |
| (38.30) | QSAFYFGYFIIPIPAGILM | LIQSAFYFGYFIIPIPAGILM | 345.08 | 17.32 | 32.25 | 1.52 |
|  | KAGIITGLFLYALGAALFW | AGIITGLFLYALGAALFWPAA | 348.20 | 339.45 | 8.75 | 2.04 |
|  | FLVGLFIIAAGLGCLETAANPFV | NYTLFLVGLFIIAAGLGCLET | 262.52 | 227.56 | 34.96 | 0.92 |
|  | NLAQTFASFGAIIAVVFGQ | AQTFASFGAIIAVVFGQSLIL | 291.77 | 261.77 | 30.00 | 1.20 |
|  | TPYMIIVAIVLLVALLIML | VQTPYMIIVAIVLLVALLIML | 154.18 | 200.28 | 46.10 | 1.75 |
|  | RWAVLAQFCYVGAQTACWSYL | WRWAVLAQFCYVGAQTACWSY | 205.66 | 146.72 | 58.94 | 0.64 |
|  | LTGTMVCFFIGRFTGTWLI | ANYLTGTMVCFFIGRFTGTWL | 159.76 | 104.50 | 55.26 | 2.02 |
|  | KVLAAYALIAMALCLISAFA | VLAAYALIAMALCLISAFAGG | 275.55 | 276.81 | 1.26 | 2.16 |
|  | GLIALTLCSAFMSIQYPTIFSLG | VGLIALTLCSAFMSIQYPTIF | 351.22 | 294.42 | 56.80 | 0.40 |
|  | YGSSFIVMTIIGGGIVTPVM | SSFIVMTIIGGGIVTPVMGFV | 232.13 | 132.35 | 99.78 | 1.08 |
|  | TAELIPALCFAVIFIFARF | IPTAELIPALCFAVIFIFARF | 159.54 | 151.25 | 8.29 | 1.74 |
| **3OE6:A** | KIFLPTIYSIIFLTGIVGNGLVIL | PTIYSIIFLTGIVGNGLVILV | 114.14 | 121.19 | 7.05 | 1.42 |
| (25.14) | RLHLSVADLLFVITLPFWAVDA | KYRLHLSVADLLFVITLPFWA | 321.82 | 311.54 | 10.28 | 0.52 |
|  | GNFLCKAVHVIYTVNLYSSVWILAF | AVHVIYTVNLYSSVWILAFIS | 263.54 | 302.09 | 38.55 | 0.66 |
|  | YVGVWIPALLLTIPDFIFAN | KVVYVGVWIPALLLTIPDFIF | 62.62 | 87.20 | 24.59 | 1.23 |
|  | WVVVFQFQHIMVGLILPGIVILS | QHIMVGLILPGIVILSCYCII | 315.73 | 4.76 | 49.03 | 1.56 |
|  | ILILAFFACWLPYYIGISIDSFILL | KTTVILILAFFACWLPYYIGI | 359.76 | 334.89 | 24.87 | 1.12 |
|  | HKWISITEALAFFHCCLNPILYA | SITEALAFFHCCLNPILYAFL | 81.37 | 103.00 | 21.64 | 1.27 |
| **3ORG:A** | LRLVCFLTLLGVTAALFIFAVDLAVHGLE | LLRLVCFLTLLGVTAALFIFA | 143.79 | 202.85 | 59.06 | 0.41 |
| (59.70) | VSGVALCLLSTFWCAVLST | YILYVVSGVALCLLSTFWCAV | 174.47 | 166.49 | 7.97 | 1.81 |
|  | RVLFAKALGLICAIGG | LRVLFAKALGLICAIGGGLPV | 129.23 | 37.86 | 91.37 | 0.99 |
|  | LAAACAVGLASSFGA | LQTLAAACAVGLASSFGAPLG | 120.50 | 12.17 | 108.33 | 0.27 |
|  | LLYAILGALMGVLGALFIRCVRSIYELR | TLLYAILGALMGVLGALFIRC | 148.16 | 153.48 | 5.32 | 2.33 |
|  | PAGVFVPSFLIGAGFG | LPAGVFVPSFLIGAGFGRLYG | 352.74 | 330.28 | 22.46 | 0.19 |
|  | LVPVLISVLLAVIVGNAFN | IRHLVPVLISVLLAVIVGNAF | 158.90 | 35.52 | 123.38 | 0.69 |

| PDB:Chain |  | TOPCONS predicted topology |  |  |  |  |
| --- | --- | --- | --- | --- | --- | --- |
| (MAAE) | PDBTM TM helix sequence | predicted TM helix sequence | observed angle | predicted angle | angular error |  |
| **3P4W:A** | PNIILPMLFILFISWTAFWS | SYIPNIILPMLFILFISWTAF | 21.30 | 333.40 | 47.90 | 1.32 |
| (24.78) | GAIIFMIYLFYFVAVIEVTV | TGAIIFMIYLFYFVAVIEVTV | 178.77 | 180.43 | 1.66 | 1.18 |
| **3P5N:A** | ISMLSAIAFVLTFIKF | LITISMLSAIAFVLTFIKFPI | 128.49 | 96.86 | 31.63 | 0.74 |
| (51.25) | TLDFSDVPSLLATF | VPSLLATFTFGPVAGIIVALV | 202.69 | 94.59 | 108.10 | 1.25 |
|  | PFNIIKGIVISIVFILLYRR | GIIPFNIIKGIVISIVFILLY | 288.85 | 302.88 | 14.03 | 1.57 |
| **3PJZ:A** | IRIVGLLLALFSVTMLAPALVALLY | VGLLLALFSVTMLAPALVALL | 127.04 | 192.87 | 65.83 | 2.17 |
| (24.68) | VPFVTTFFVLLFCGAMCWFPNR | AGVPFVTTFFVLLFCGAMCWF | 210.26 | 201.54 | 8.72 | 2.29 |
|  | FLIVVLFWTVLGSAGSLPFLIA | FLIVVLFWTVLGSAGSLPFLI | 134.35 | 169.69 | 35.34 | 1.10 |
|  | AILFYRQFLQWFGGMGIIVLAVAI | FLQWFGGMGIIVLAVAILPVL | 326.27 | 305.61 | 20.66 | 0.52 |
|  | KALWYIYLSLTIACAVAFWLAGMT | LWYIYLSLTIACAVAFWLAGM | 217.07 | 214.65 | 2.42 | 2.33 |
|  | YAINLITVVFLLISACNFTLHF | AINLITVVFLLISACNFTLHF | 322.69 | 329.39 | 6.70 | 1.60 |
|  | EFRAFIFIQVLLFLVCFLLLLK | AFIFIQVLLFLVCFLLLLKHH | 297.12 | 328.83 | 31.71 | 1.55 |
|  | LFLPVLLLFSSFIGGCAG | FLPVLLLFSSFIGGCAGSTGG | 320.19 | 333.63 | 13.44 | 0.45 |
|  | WGFFSAYALVFVVCMLGLIATGMD | DAVWGFFSAYALVFVVCMLGL | 330.44 | 2.03 | 31.59 | 1.56 |
|  | KAKWVLIVSMLFGRLEIFTLL | WVLIVSMLFGRLEIFTLLILL | 40.12 | 9.71 | 30.41 | 1.02 |
