# Supplementary material for: Lipid exposure prediction enhances the inference of rotational angles of transmembrane helices
Source: BMC Bioinformatics. 2013 Oct 11;14:304. doi: 10.1186/1471-2105-14-304 (PMC3854514; doi:10.1186/1471-2105-14-304)
Supplement: Additional file 5: Table S3 — Sequences, observed angles defined by residues of predicted topology (by MemBrain), predicted angles, moment lengths and MAAE on the 155 TMHs of the independent test set. [file 1471-2105-14-304-S5.docx]

Table S3. Sequences, observed angles defined by residues of predicted topology (by MemBrain), predicted angles, moment lengths and MAAE on the 155 TMHs of the independent test set.

| PDB:Chain |  | MemBrain predicted topology |  |  |  |  |
| --- | --- | --- | --- | --- | --- | --- |
| (MAAE) | PDBTM TM helix sequence | predicted TM helix sequence | observed angle | predicted angle | angular error |  |
| **2XQ2:A** | AVGASLIAANISAEQF | PWWAVGASLIAANISAE | 328.20 | 170.02 | 158.18 | 0.54 |
| (61.85) | ASYEWMSAITLIIV | GMSGSGYSIGLAIASYEWMSAITLIIVGKYFLPI | 173.06 | 262.01 | 88.95 | 0.84 |
|  | LAVFWISLYIFVNLTSVLYLG | KTILAVFWISLYIFVNLTSVLYLGGLALE | 130.53 | 123.97 | 6.56 | 0.70 |
|  | VWTDVIQVFFLVLGG | WTDVIQVFFLVLGGFMTTYMAVSFIGG | 25.35 | 5.48 | 19.87 | 2.44 |
|  | AVLIGGLWVANLYYWGFNQ | NLPGIAVLIGGLWVANLYYWGFNQYII | 222.00 | 140.41 | 81.59 | 0.39 |
|  | VFAAFLALIVPFLV | GIVFAAFLALIVPFLVVLPGIAAYVITS | 254.37 | 245.18 | 9.19 | 1.90 |
|  | ALAAAIVSSLASMLNSTA | VVFAALAAAIVSSLASMLNSTA | 283.53 | 177.40 | 106.13 | 0.98 |
|  | RTAAVVALIIAALIA | NVGRTAAVVALIIAALIAPMLGG | 86.03 | 48.97 | 37.06 | 2.40 |
|  | QYIQEYTGLVSPGILA | GQCFQYIQEYTGLVSPGILAVFLLGLFW | 109.03 | 5.58 | 103.45 | 0.15 |
|  | VVASIPFALFLKFM | AIIGVVASIPFALFLKFMPLS | 234.82 | 186.49 | 48.33 | 1.49 |
|  | FMDQMLYTLLFTMVV | QMLYTLLFTMVVIAFTSLST | 315.61 | 259.54 | 56.07 | 1.54 |
|  | AAYGIMIVLAVLYT | AAYGIMIVLAVLYTLFWV | 249.70 | 241.71 | 7.99 | 0.91 |
|  | GVMAGVIGTILLISYGIK | ADAEITLIIFGVMAGVIGTILLISYGI | 219.20 | 299.84 | 80.64 | 0.13 |
| **2XUT:A** | ASEACERFSFYGMRNILTPFL | YGMRNILTPFLMT | 134.44 | 57.71 | 76.73 | 0.44 |
| (63.47) | VAKDVFHSFVIGVYFFPLLG | DVFHSFVIGVYFFPLLGGWIA | 51.45 | 9.77 | 41.68 | 1.01 |
|  | TILWLSLIYCVGHAFLAIFE | NTILWLSLIYCVGHAFLAIFEHS | 336.15 | 354.67 | 18.52 | 1.99 |
|  | GFYTGLFLIALGSGGIKP | QGFYTGLFLIALGSGGIKPLV | 101.75 | 10.64 | 91.11 | 0.75 |
|  | YFTINFGSFFASLSMPLLL | DMFYFTINFGSFFASLSMPLL | 41.39 | 106.03 | 64.64 | 1.26 |
|  | VAFGIPGVLMFVATVFFWL | AAVAFGIPGVLMFVATVFFWLG | 126.25 | 174.77 | 48.52 | 1.41 |
|  | IGGVSAAYALVNIPTL | EGKGNIGLVLALIGGVSA | 7.94 | 73.08 | 65.14 | 0.22 |
|  | IVAGLCCAMVLVMGFVG | NIPTLGIVAGLCCAMVLVMGFVG | 10.60 | 349.07 | 21.53 | 0.27 |
|  | ALVTPFWSLFDQKASTWILQA | RILVLFALVTPFWSLFDQK | 159.10 | 263.63 | 104.53 | 0.43 |
|  | GAGIAITGLSWIVVGTIQ | KMGAGIAITGLSWIVVGTIQ | 292.03 | 270.12 | 21.91 | 1.75 |
|  | SIFWQILPYALLTFGEVLVS | FWQILPYALLTFGEV | 51.82 | 348.96 | 62.86 | 0.55 |
|  | FWTLSVTVGNLWVLLANVSV | KGTIMSFWTLSVTVGNLWVLLANVSV | 69.16 | 213.62 | 144.46 | 1.07 |
| **3KBC:A** | QKILIGLILGAIVGLI | LQKILIGLILGAIVGLILG | 150.18 | 135.38 | 14.80 | 1.10 |
| (45.50) | VKPFGDLFVRLLCMLVMPIVFASL | VRLLCMLVMPIVFASLVVG | 169.70 | 134.24 | 35.46 | 0.58 |
|  | AFAVTLGIIMARLFNPG | GVKIVVYYLLTSAFAVTLGIIMARLFN | 212.84 | 187.05 | 25.79 | 1.71 |
|  | QVLPTIFFAIIL | QVLPTIFFAIILGIAIT | 293.05 | 219.99 | 73.06 | 0.70 |
|  | VMQYAPIGVFALIAYVM | AMYKIVNGVMQYAPIGVFALIAYVMA | 238.01 | 354.49 | 116.48 | 1.16 |
|  | VGELAKVTAAVYVGLTLQ | VGELAKVTAAVYVGLTLQILLVYFVLLKIYGI | 86.82 | 79.01 | 7.81 | 1.52 |
|  | GTALYQGVATFFIA | TALYQGVATFFIANA | 175.98 | 237.11 | 61.13 | 0.15 |
|  | GAGAIMLCMVLHSVGLPLT | GVPGAGAIMLCMVLHSV | 265.32 | 294.81 | 29.49 | 0.37 |

| PDB:Chain |  | MemBrain predicted topology |  |  |  |  |
| --- | --- | --- | --- | --- | --- | --- |
| (MAAE) | PDBTM TM helix sequence | predicted TM helix sequence | observed angle | predicted angle | angular error |  |
| **3KCU:A** | YLAITAGVFISIAFVFYITATTGT | KTFYLAITAGVFISIAFVFYITATTG | 282.45 | 271.26 | 11.19 | 0.99 |
| (32.88) | PFGMAKLVGGICFSLGLILCV | GMAKLVGGICFSLGLILCVVCGA | 123.98 | 94.41 | 29.57 | 0.95 |
|  | LNVYFGNLVGALLFVLLMWLSGEYMTA | LAKNWLNVYFGNLVGALLFVLLMWLSGEY | 196.76 | 194.07 | 2.69 | 2.58 |
|  | GLNVLQTADHKVHHTFIEAVCLGILANLMVCLAV | EAVCLGILANLMVCLAVWMSYS | 273.87 | 320.06 | 46.19 | 1.08 |
|  | FIMVLPVAMFVA | LMDKAFIMVLPVAMFVASGFEH | 142.17 | 117.60 | 24.57 | 0.72 |
|  | LTVMNFITDNLIPVTIGNIIGGGLL | ITDNLIPVTIGNIIGGGLLVGLTYWV | 109.35 | 192.43 | 83.08 | 0.64 |
| **3KG2:A** | AYEIWMCIVFAYIGVSVVLF | LAYEIWMCIVFAYIGVSVVLFLVS | 226.50 | 250.87 | 24.37 | 1.72 |
| (26.95) | SGRIVGGVWWFFTLIIISSYT | SGRIVGGVWWFFTLIIISSYTAN | 263.50 | 244.34 | 19.16 | 1.22 |
|  | VAGVFYILVGGLGLAMLVAL | VAGVFYILVGGLGLAMLVALIEFCY | 81.29 | 118.60 | 37.31 | 1.66 |
| **3KP9:A** | ILAILAGLGSLLTAYLTYT | LAILAGLGSLLTAY | 174.87 | 188.43 | 13.56 | 1.50 |
| (37.37) | AEFLGIPTAAVGLLGFLGVLALAVL | IPTAAVGLLGFLGVLALAVLP | 327.74 | 75.79 | 108.05 | 0.85 |
|  | LFGLVSAMTAFEMYMLYLMVA | LFGLVSAMTAFEMYMLYLMVAVLRQF | 152.00 | 194.16 | 42.16 | 1.94 |
|  | CMYCTTAIILVAGLGLVTV | MYCTTAIILVAGLGLVT | 13.97 | 10.64 | 3.33 | 1.39 |
|  | FSYILVAFLTLVTTIGVYANQ | AFSYILVAFLTLVTTIG | 118.58 | 98.85 | 19.73 | 0.73 |
| **3L1L:A** | GLIPVTLMVSGAIMGSGVFLLP | IPVTLMVSGAIMGSGVFLLPANLA | 0.11 | 355.73 | 4.38 | 0.50 |
| (37.43) | IYGWLVTIIGALGLSMVYAK | TGGIAIYGWLVTIIGALGLSMVYAKMSFL | 170.60 | 215.86 | 45.26 | 1.94 |
|  | LGYQTNVLYWLACWIGNIAMVVIGVGYL | GYQTNVLYWLACWIGNIAMVVIGVGYLSYFF | 140.16 | 127.38 | 12.78 | 0.94 |
|  | LTITCVVVLWIFVLLNIVGP | DPWVLTITCVVVLWIFVLLNIVGPK | 255.25 | 285.28 | 30.03 | 1.90 |
|  | MITRVQAVATVLALIPIVGIAVFGW | TRVQAVATVLALIPIVGIAVFGWFWF | 116.44 | 118.96 | 2.52 | 2.59 |
|  | PIATIGGVLIAAVCYVLSTTA | ATIGGVLIAAVCYVLSTTAIMGMIP | 263.60 | 276.33 | 12.73 | 1.66 |
|  | VSFCAAAGCLGSLGGWTLLAGQTAK | DTAGAIVSFCAAAGCLGSLGGWTLL | 197.78 | 70.19 | 127.59 | 0.98 |
|  | VAGLIIVGILMTIFQLSSISPNA | VAGLIIVGILMTIFQLSSISP | 90.60 | 99.50 | 8.91 | 1.56 |
|  | FGLVSSVSVIFTLVPYLYTCAAL | EFGLVSSVSVIFTLVPYLYTCAALLLL | 132.50 | 285.77 | 153.27 | 1.50 |
|  | AYLAVTTIAFLYCIWAVVGSGA | AYLAVTTIAFLYCIWAVVGS | 282.47 | 280.73 | 1.74 | 1.74 |
|  | EVMWSFVTLMVITAMYALN | EVMWSFVTLMVITAMYALNY | 235.84 | 223.35 | 12.49 | 1.59 |
| **3M71:A** | GYFGIPLGLAALSLAW | GYFGIPLGLAALSLAWFH | 154.23 | 189.46 | 35.23 | 0.41 |
| (60.22) | SDVLGIVASAVWILFILMYAY | ARMVSDVLGIVASAVWILFILMYAYKL | 182.26 | 222.71 | 40.45 | 2.36 |
|  | FIALIPITTMLVGDIL | FSFIALIPITTMLVGDILYR | 317.97 | 317.39 | 0.58 | 0.38 |
|  | AEVLIWIGTIGQLLFSTLRVS | NPLIAEVLIWIGTIGQLLFSTLRVS | 208.87 | 257.57 | 48.70 | 1.82 |
|  | SFYLPAVAANFTSA | LPAVAANFTSASSLALLG | 156.26 | 17.36 | 138.90 | 0.30 |
|  | YLFFGAGMIAWIIFEPVLL | YLFFGAGMIAWIIFEPVLLQ | 99.18 | 81.00 | 18.18 | 1.59 |
|  | MGIVLAPAFVCVSAY | MGIVLAPAFVCVSA | 355.76 | 28.65 | 32.89 | 0.68 |
|  | AKILWGYGFLQLFFLLRLF | KILWGYGFLQLFFLLRLFPWI | 84.66 | 34.34 | 50.32 | 0.95 |
|  | GLWAFSFGLASMANSA | IGLWAFSFGLASM | 266.86 | 359.35 | 92.49 | 0.10 |
|  | SIFAFVFSNVMIGLLVLMTIYKLT | VLQGVSIFAFVFSNVMIGLLVLMTI | 171.90 | 316.36 | 144.46 | 2.77 |

| PDB:Chain |  | MemBrain predicted topology |  |  |  |  |
| --- | --- | --- | --- | --- | --- | --- |
| (MAAE) | PDBTM TM helix sequence | predicted TM helix sequence | observed angle | predicted angle | angular error |  |
| **3MK7:A** | QFAIMTVVWGIVGMGLGV | VVRQFAIMTVVWGIVGMGLGVFIAAQ | 237.10 | 275.51 | 38.41 | 1.70 |
| (73.46) | HTNAVIFAFGGCALFATS | FGRLRPLHTNAVIFAFGGCALFATSYYSVQRT | 258.17 | 196.34 | 61.83 | 0.64 |
|  | LAAFTFWGWQLVILLAA | LAAFTFWGWQLVILLAAISLPLGF | 288.40 | 254.32 | 34.08 | 1.55 |
|  | IDILITIVWVAYAVVFF | ELEWPIDILITIVWVAYAVVFFGTLA | 167.20 | 27.14 | 140.06 | 1.13 |
|  | WFFGAFILTVAILHV | GNWFFGAFILTVAILHVVNNLEIPVTAMKSYSL | 148.51 | 233.58 | 85.07 | 0.27 |
|  | HNAVGFFLTAGFLGIM | WWYGHNAVGFFLTAGFLGIMYYFVP | 94.35 | 345.59 | 108.76 | 0.19 |
|  | IVHFWALITVYIWA | YRLSIVHFWALITVYIWAGP | 170.49 | 118.65 | 51.84 | 1.50 |
|  | MSLILLAPSWGGMI | DWAQSLGMVMSLILLAPSWGGMINGMMTL | 125.02 | 37.37 | 87.65 | 1.79 |
|  | FLVVSLAFYGMSTFEGPM | DPILRFLVVSLAFYGMSTFEGPM | 355.46 | 358.79 | 3.33 | 0.58 |
|  | GHVHAGALGWVAMVSI | DWTIGHVHAGALGWVAMVSIGALYHLVPK | 83.48 | 282.67 | 160.81 | 0.65 |
|  | THFWLATIGTVLYIA | GLINTHFWLATIGTVLYIASMWVNGI | 355.53 | 3.90 | 8.37 | 2.41 |
|  | RMIGGAIFFAGMLVMAY | YSFVESLEASHPGFVVRMIGGAIFFAGMLVMAYNTWRTV | 208.14 | 309.44 | 101.30 | 3.63 |
| **3MK7:C** | LTLGTIVALFWLIFA | GYIALLTLGTIVALFWLIFA | 88.43 | 35.38 | 53.05 | 1.82 |
| (72.76) | RWWFLLFIGTLVFGI | WWFLLFIGTLVFGILYLVLYP | 11.76 | 279.28 | 92.48 | 2.01 |
| **3MKT:A** | ATPVLIASVAQTGMGFVD | IKLATPVLIASVAQTGMGFVDTIMA | 97.87 | 53.60 | 44.27 | 2.04 |
| (61.26) | WLPSILFGVGLLMALVPV | AVSIAASIWLPSILFGVGLLMALVPV | 238.18 | 350.09 | 111.91 | 0.64 |
|  | QGLILALLVSVPIIAVLF | QGLILALLVSVPIIAVLFQTQFI | 305.90 | 330.84 | 24.94 | 2.00 |
|  | MHAVIFAVPAYLLFQALRSF | GYMHAVIFAVPAYLLFQALRS | 138.96 | 287.10 | 148.14 | 0.61 |
|  | KPAMVIGFIGLLLNIPL | KPAMVIGFIGLLLNIPLNWIFVYGK | 289.72 | 56.82 | 127.10 | 1.96 |
|  | ATAIVYWIMLLLLLFYIV | VGCGVATAIVYWIMLLLLLFYIV | 41.40 | 52.51 | 11.12 | 1.82 |
|  | IRLFRLGFPVAAALFFEVTLFAVVA | LGFPVAAALFFEVTLFAVVALLVAPLG | 308.24 | 236.73 | 71.51 | 1.80 |
|  | ALNFSSLVFMFPMSIGAA | HQVALNFSSLVFMFPMSIGAAVSIRV | 221.19 | 248.99 | 27.80 | 0.66 |
|  | GLATACITALLTVLFREQIA | AANVGLMTGLATACITALLTVLFREQI | 219.86 | 260.30 | 40.44 | 1.71 |
|  | QLLLFAAIYQCMDAVQVVAAGS | LAMQLLLFAAIYQCMDAVQVVAA | 227.13 | 260.65 | 33.53 | 0.95 |
|  | AIFHRTFISYWVLGLPTGYILGMT | MTAIFHRTFISYWVLGLPTGYILGMTNWL | 252.72 | 220.18 | 32.54 | 1.25 |
|  | GFWLGFIIGLSAAALML | GAKGFWLGFIIGLSAAALMLGQRLY | 330.16 | 268.38 | 61.78 | 1.12 |
| **3MP7:A** | GVALILYYVLAEIPV | RFMWTGVALILYYVLAEI | 224.86 | 181.35 | 43.51 | 1.64 |
| (82.60) | VFSVFMCFFEAAVWI | QRVFSVFMCFFEAAVWILGGAFGR | 226.33 | 224.73 | 1.60 | 1.58 |
|  | ISLFIAAGVSQTILTRSLNP | GSGISLFIAAGVSQTILT | 346.75 | 205.96 | 140.79 | 0.02 |
|  | DMLSVVATIVVFFIVVYF | GGSAPDMLSVVATIVVFFIVVYFE | 270.77 | 300.75 | 29.98 | 2.20 |
|  | NIPIILTFALYANIQLWA | YVSNIPIILTFALYANIQLWARVLDRL | 349.85 | 121.99 | 132.14 | 0.93 |
|  | VRAIVYLILTVIFSLLFG | DNPVRAIVYLILTVIFSLLFGYLWVE | 185.94 | 296.16 | 110.22 | 2.17 |
|  | ALGTGTGILLTVGIL | LGTGTGILLTVGILYR | 61.70 | 181.65 | 119.95 | 0.09 |

| PDB:Chain |  | MemBrain predicted topology |  |  |  |  |
| --- | --- | --- | --- | --- | --- | --- |
| (MAAE) | PDBTM TM helix sequence | predicted TM helix sequence | observed angle | predicted angle | angular error |  |
| **3NYM:A**(87.06) | IASNSLFMAMIYAGNLSLIFD | SLFMAMIYAGNLS | 107.98 | 20.92 | 87.06 | 0.29 |
| **3O0R:B** | YFVFALILFVGQILFGLIMGL | VAKPYFVFALILFVGQILFGLIMGLQYVV | 232.35 | 278.36 | 46.01 | 2.65 |
| (70.23) | ARMVHTNLLIVWLLFGFMGAAY | RMVHTNLLIVWLLFGFMGAAYYLV | 215.74 | 72.01 | 143.73 | 0.19 |
|  | LAWILFWVFAAAGVLTILGYLL | KLAWILFWVFAAAGVLTILGYLLV | 25.55 | 20.10 | 5.45 | 1.75 |
|  | FLEQPTISKAGIVIVALGFLFNVGM | PTISKAGIVIVALGFLFNVGMTV | 189.05 | 170.79 | 18.26 | 0.88 |
|  | VLMTGLIGLALLFLFSFY | AISMVLMTGLIGLALLFLFSF | 289.62 | 272.52 | 17.10 | 0.82 |
|  | WWWVVHLWVEGVWELIMGAIL | LWVEGVWELIMGAILAFVLVK | 326.48 | 147.04 | 179.44 | 0.90 |
|  | YVIIAMALISGIIGTGHH | EKWLYVIIAMALISGIIGTGHH | 125.91 | 119.02 | 6.89 | 0.71 |
|  | LGSVFSALEPLPFFAMVLF | GYWLWLGSVFSALEPLPFFAMVLFAFNT | 169.40 | 51.51 | 117.89 | 1.61 |
|  | AMGTTVMAFLGAGVWGFMHTL | VALWAMGTTVMAFLGAGVWGFMHTLAPV | 165.00 | 243.29 | 78.29 | 0.95 |
|  | LTAAHGHMAFYGAYAMIVMTII | QLTAAHGHMAFYGAYAMIVMTIISYAMP | 65.10 | 245.49 | 179.61 | 0.20 |
|  | WGFWLMTVAMVFITLFLSAA | MWGFWLMTVAMVFITLFLSAAGVLQ | 94.22 | 75.34 | 18.88 | 2.81 |
|  | FYWLREGAGVVFLIGLVAYLL | IFYWLREGAGVVFLIGLVAYLLS | 332.39 | 301.13 | 31.26 | 2.19 |
| **3O7P:A** | LLCSLFFLWAVANNLNDILL | IPFALLCSLFFLWAVANNLN | 1.83 | 2.15 | 0.32 | 1.17 |
| (43.95) | QSAFYFGYFIIPIPAGILM | AGLIQSAFYFGYFIIPIPAGI | 280.07 | 210.30 | 69.77 | 1.49 |
|  | KAGIITGLFLYALGAALFW | KAGIITGLFLYALGAALFWP | 95.27 | 74.71 | 20.56 | 1.86 |
|  | FLVGLFIIAAGLGCLETAANPFV | EIMNYTLFLVGLFIIAAGLGCLETAANP | 266.59 | 160.95 | 105.64 | 1.11 |
|  | NLAQTFASFGAIIAVVFGQ | FASFGAIIAVVFGQSLI | 340.20 | 320.89 | 19.31 | 1.22 |
|  | TPYMIIVAIVLLVALLIML | TPYMIIVAIVLLVALLIMLT | 0.70 | 358.39 | 2.31 | 1.68 |
|  | RWAVLAQFCYVGAQTACWSYL | RWAVLAQFCYVGAQTACWSYLIR | 90.53 | 50.01 | 40.52 | 0.66 |
|  | LTGTMVCFFIGRFTGTWLI | LTGTMVCFFIGRFTGT | 243.60 | 163.39 | 80.21 | 1.52 |
|  | KVLAAYALIAMALCLISAFA | VLAAYALIAMALCLISAFA | 251.68 | 274.58 | 22.90 | 2.14 |
|  | GLIALTLCSAFMSIQYPTIFSLG | VGLIALTLCSAFMSIQYPTIFSL | 334.30 | 308.27 | 26.03 | 0.41 |
|  | YGSSFIVMTIIGGGIVTPVM | GSSFIVMTIIGGGIVTPVM | 317.84 | 221.94 | 95.90 | 0.73 |
|  | TAELIPALCFAVIFIFARF | PTAELIPALCFAVIFIFAR | 94.68 | 50.72 | 43.96 | 1.82 |
| **3OE6:A** | KIFLPTIYSIIFLTGIVGNGLVIL | NKIFLPTIYSIIFLTGIVGNGLVILVMG | 176.76 | 256.15 | 79.39 | 1.58 |
| (49.23) | RLHLSVADLLFVITLPFWAVDA | DKYRLHLSVADLLFVITLPFWAVDA | 56.60 | 53.73 | 2.87 | 0.47 |
|  | GNFLCKAVHVIYTVNLYSSVWILAF | LCKAVHVIYTVNLYSSVWILAFISLD | 242.92 | 254.37 | 11.45 | 0.93 |
|  | YVGVWIPALLLTIPDFIFAN | AEKVVYVGVWIPALLLTIPDFIF | 173.67 | 286.07 | 112.41 | 1.20 |
|  | WVVVFQFQHIMVGLILPGIVILS | VVFQFQHIMVGLILPGIVILSCYCIII | 25.73 | 137.16 | 111.43 | 1.68 |
|  | ILILAFFACWLPYYIGISIDSFILL | LKTTVILILAFFACWLPYYIGISIDSFIL | 71.03 | 84.14 | 13.11 | 1.28 |
|  | HKWISITEALAFFHCCLNPILYA | VHKWISITEALAFFHCCLNPILYAF | 258.75 | 244.79 | 13.96 | 1.43 |
| **3ORG:A** | LRLVCFLTLLGVTAALFIFAVDLAVHGLE | YLLRLVCFLTLLGVTAALFIFAVDLAVH | 247.81 | 265.57 | 17.75 | 0.98 |
| (72.72) | VSGVALCLLSTFWCAVLST | RFAGYILYVVSGVALCLLSTFWCAV | 233.38 | 195.65 | 37.73 | 1.84 |
|  | RVLFAKALGLICAIGG | RVLFAKALGLICAIGGGL | 350.73 | 301.19 | 49.54 | 0.83 |
|  | LAAACAVGLASSFGA | LAAACAVGLASSFGAPLGGVLYSIETIA | 51.75 | 118.54 | 66.79 | 0.26 |
|  | LLYAILGALMGVLGALFIRCVRSIYELR | TQTLLYAILGALMGVLGALFIRCV | 172.94 | 349.65 | 176.71 | 2.66 |
|  | PAGVFVPSFLIGAGFG | GVFVPSFLIGAGFGRLYGELMRVV | 319.42 | 69.99 | 110.57 | 0.51 |
|  | LVPVLISVLLAVIVGNAFN | QIRHLVPVLISVLLAVIVG | 178.35 | 128.39 | 49.96 | 0.55 |

| PDB:Chain |  | MemBrain predicted topology |  |  |  |  |
| --- | --- | --- | --- | --- | --- | --- |
| (MAAE) | PDBTM TM helix sequence | predicted TM helix sequence | observed angle | predicted angle | angular error |  |
| **3P4W:A** | PNIILPMLFILFISWTAFWS | FSYIPNIILPMLFILFISWTAFWS | 82.50 | 70.23 | 12.27 | 1.47 |
| (61.27) | GAIIFMIYLFYFVAVIEVTV | MTYTGAIIFMIYLFYFVAVIEVTVQHY | 359.59 | 109.86 | 110.27 | 1.24 |
| **3P5N:A** | ISMLSAIAFVLTFIKF | SMLSAIAFVLTFIKFPI | 101.18 | 58.26 | 42.92 | 0.81 |
| (51.01) | TLDFSDVPSLLATF | PSLLATFTFGPVAGI | 233.52 | 335.16 | 101.64 | 0.87 |
|  | PFNIIKGIVISIVFILLYRR | IVSGIIPFNIIKGIVISIVFILLYR | 231.60 | 240.07 | 8.47 | 1.92 |
| **3PJZ:A** | IRIVGLLLALFSVTMLAPALVALLY | IRIVGLLLALFSVTMLAPALVALL | 98.92 | 129.82 | 30.90 | 2.26 |
| (43.20) | VPFVTTFFVLLFCGAMCWFPNR | VPFVTTFFVLLFCGAMCWF | 331.40 | 1.90 | 30.50 | 2.22 |
|  | FLIVVLFWTVLGSAGSLPFLIA | DGFLIVVLFWTVLGSAGSLPF | 341.42 | 0.15 | 18.73 | 0.85 |
|  | AILFYRQFLQWFGGMGIIVLAVAI | YRQFLQWFGGMGIIVLAVAILPVLGIGG | 273.49 | 258.17 | 15.32 | 0.64 |
|  | KALWYIYLSLTIACAVAFWLAGMT | TAKALWYIYLSLTIACAVAFWLAGMTPFDAISHS | 166.54 | 247.62 | 81.08 | 2.30 |
|  | YAINLITVVFLLISACNFTLHF | YAINLITVVFLLISACNFTLHF | 55.88 | 68.88 | 13.00 | 1.61 |
|  | EFRAFIFIQVLLFLVCFLLLLK | RAFIFIQVLLFLVCFLLLLKHH | 22.58 | 66.40 | 43.82 | 1.56 |
|  | LFLPVLLLFSSFIGGCAG | PLFLPVLLLFSSFIGG | 291.24 | 163.60 | 127.64 | 0.55 |
|  | WGFFSAYALVFVVCMLGLIATGMD | VDAVWGFFSAYALVFVVCMLGLIATGM | 73.91 | 110.97 | 37.06 | 1.33 |
|  | KAKWVLIVSMLFGRLEIFTLL | DKAKWVLIVSMLFGRLEIFTLLILLTPTFW | 19.16 | 53.10 | 33.94 | 1.45 |
