# Supplementary material for: Lipid exposure prediction enhances the inference of rotational angles of transmembrane helices
Source: BMC Bioinformatics. 2013 Oct 11;14:304. doi: 10.1186/1471-2105-14-304 (PMC3854514; doi:10.1186/1471-2105-14-304)
Supplement: Additional file 6: Table S4 — Evaluation measures used in this work. [file 1471-2105-14-304-S6.docx]

Table S4. Evaluation measures used in this work.

| Measure | Equation |
| --- | --- |
| Pearson correlation coefficient | $\frac{\sum_{i=1}^{N} \left(predicted_{i}-\overline{predicted_{i}}\right)\left(observed_{i}-\overline{observed_{i}}\right)}{\sqrt{\sum_{i=1}^{N} \left(predicted_{i}-\overline{predicted_{i}}\right)^{2}\sum_{i=1}^{N} \left(observed_{i}-\overline{observed_{i}}\right)^{2}}}$ |
| Accuracy | $\left(TN+TP\right)/\left(TP+FP+TN+FN\right)$ |
| Sensitivity | $TP/\left(TP+FN\right)$ |
| Specificity | $TN/\left(TN+FP\right)$ |
| Precision | $TP/\left(TP+FP\right)$ |
