# Supplementary material for: Lipid exposure prediction enhances the inference of rotational angles of transmembrane helices
Source: BMC Bioinformatics. 2013 Oct 11;14:304. doi: 10.1186/1471-2105-14-304 (PMC3854514; doi:10.1186/1471-2105-14-304)
Supplement: Additional file 8: Text S1 — Details of LOOCV performances during model development. [file 1471-2105-14-304-S8.pdf]

An SVM-based predictor for lipid exposure of TM helices

Details of LOOCV performances during model development

In TMexpo, we simply followed conventional grid search within certain parameter ranges and calculated performances of LOOCV to determine the best set of parameters to train the development model. The parameters of the models were optimized by chain-wise LOOCV procedure on the development set. Indeed, this procedure is heavily time-consuming and needs extreme large amount of space to store all models. For classifying burial/exposed status of TM residues, we adopted RBF kernel and searched parameters within all combinations of cost *c* (2^0, 2^1, 2^2, 2^3, 2^4, 2^5) and gamma *g* (2^{-6}, 2^{-5}, 2^{-4}, 2^{-3}). For real-number rASA regression prediction of TM residues, we also adopted RBF kernel and searched parameters within all combinations of cost *c* (2^{-2}, 2^{-1}, 2^0, 2^1, 2^2), gamma *g* (2^{-6}, 2^{-5}, 2^{-4}, 2^{-3}), loss function *p* (10^{-4}, 10^{-3}, 10^{-2}) and tolerance of termination criterion *e* (10^{-3}, 10^{-2}, 10^{-1}). Table A1 and Table A2 demonstrate the performance corresponding to each parameter. Table A1 is sorted by MCC (Matthew's correlation coefficient) value and Table A2 is sorted by PCC (Pearson correlation coefficient) value. Finally, the best set of parameters to train the exposed/buried residues classification model is of cost $c = 2^1$ and gamma $g = 2^{-4}$; and the best set of parameters to train the real-number rASA regression model is of cost $c = 2^{-1}$, gamma $g = 2^{-5}$, loss function $p = 10^{-3}$ and tolerance of termination criterion $e = 10^{-2}$.

Table A1. LOOCV performances correspond to parameters for classifying exposed/buried residues of TM residues. The table is sorted by MCC (Matthew's correlation coefficient) value, and the best set of parameters for model development is selected by the best MCC value.

c (cost)	g (gamma)	MCC	accuracy	sensitivity	specificity	precision
**2**	**0.0625**	**0.5197**	**0.7632**	**0.8039**	**0.7131**	**0.7752**
4	0.0625	0.5183	0.7625	0.8035	0.7121	0.7745
8	0.0625	0.5180	0.7624	0.8033	0.7121	0.7744
16	0.0625	0.5180	0.7624	0.8033	0.7121	0.7744
32	0.0625	0.5180	0.7624	0.8033	0.7121	0.7744
1	0.03125	0.5154	0.7603	0.7832	0.7321	0.7825
2	0.03125	0.5121	0.7587	0.7811	0.7311	0.7814
4	0.03125	0.5113	0.7584	0.7844	0.7264	0.7792
8	0.03125	0.5102	0.7580	0.7847	0.7251	0.7784
16	0.03125	0.5093	0.7575	0.7842	0.7246	0.7780
32	0.03125	0.5093	0.7575	0.7842	0.7246	0.7780
1	0.0625	0.5088	0.7578	0.7984	0.7079	0.7708
2	0.015625	0.5052	0.7550	0.7743	0.7314	0.7801
1	0.015625	0.5014	0.7534	0.7768	0.7246	0.7763
4	0.015625	0.4975	0.7513	0.7717	0.7261	0.7762
8	0.015625	0.4907	0.7481	0.7724	0.7183	0.7714
32	0.015625	0.4906	0.7480	0.7717	0.7188	0.7716
16	0.015625	0.4891	0.7473	0.7713	0.7178	0.7708
2	0.125	0.4284	0.7108	0.9146	0.4601	0.6758
4	0.125	0.4282	0.7107	0.9146	0.4598	0.6757
8	0.125	0.4282	0.7107	0.9146	0.4598	0.6757
16	0.125	0.4282	0.7107	0.9146	0.4598	0.6757
32	0.125	0.4282	0.7107	0.9146	0.4598	0.6757
1	0.125	0.3905	0.6885	0.9320	0.3889	0.6524

Table A2. LOOCV performances correspond to parameters for real-number rASA regression model of TM residues. The table is sorted by PCC (Pearson correlation coefficient) value, and the best set of parameters for model development is selected by the best PCC value. RMSD stands for root-mean-square deviation, and MAE stands for mean absolute error.

c (cost)	g (gamma)	p (loss function)	e (tolerance of termination criterion)	PCC	RMSD	MAE
**0.5**	**0.03125**	**0.001**	**0.01**	**0.6549**	**0.1745**	**0.1280**
0.5	0.03125	0.0001	0.01	0.6548	0.1745	0.1279
0.5	0.03125	0.0001	0.001	0.6548	0.1745	0.1279
0.5	0.03125	0.001	0.001	0.6548	0.1745	0.1280
0.5	0.03125	0.01	0.01	0.6546	0.1745	0.1285
0.5	0.03125	0.01	0.001	0.6546	0.1745	0.1285
0.5	0.03125	0.001	0.1	0.6538	0.1746	0.1286
0.5	0.03125	0.01	0.1	0.6533	0.1747	0.1293
0.5	0.03125	0.0001	0.1	0.6532	0.1748	0.1286
1	0.03125	0.0001	0.1	0.6527	0.1748	0.1302
1	0.03125	0.0001	0.001	0.6526	0.1749	0.1298
1	0.03125	0.001	0.01	0.6526	0.1749	0.1299
1	0.03125	0.001	0.001	0.6526	0.1749	0.1299
1	0.03125	0.0001	0.01	0.6526	0.1749	0.1299
1	0.03125	0.01	0.001	0.6520	0.1749	0.1304
1	0.03125	0.01	0.01	0.6520	0.1750	0.1304
1	0.03125	0.001	0.1	0.6519	0.1750	0.1303
2	0.03125	0.0001	0.1	0.6513	0.1751	0.1305
2	0.03125	0.0001	0.01	0.6512	0.1751	0.1302
2	0.03125	0.001	0.01	0.6512	0.1752	0.1302
2	0.03125	0.0001	0.001	0.6512	0.1752	0.1302
4	0.03125	0.0001	0.01	0.6512	0.1752	0.1302
4	0.03125	0.0001	0.001	0.6511	0.1752	0.1302
2	0.03125	0.001	0.001	0.6511	0.1752	0.1302
4	0.03125	0.001	0.001	0.6511	0.1752	0.1302
4	0.03125	0.001	0.01	0.6511	0.1752	0.1302
4	0.03125	0.0001	0.1	0.6509	0.1752	0.1306
1	0.03125	0.01	0.1	0.6508	0.1752	0.1311
4	0.03125	0.001	0.1	0.6508	0.1752	0.1307
2	0.03125	0.01	0.001	0.6507	0.1752	0.1307
4	0.03125	0.01	0.01	0.6507	0.1752	0.1307
4	0.03125	0.01	0.001	0.6507	0.1752	0.1307
2	0.03125	0.01	0.01	0.6506	0.1752	0.1307
2	0.03125	0.001	0.1	0.6504	0.1753	0.1308
4	0.03125	0.01	0.1	0.6501	0.1753	0.1314

0.25	0.03125	0.01	0.01	0.6499	0.1763	0.1267
0.25	0.03125	0.0001	0.001	0.6499	0.1766	0.1262
0.25	0.03125	0.001	0.01	0.6499	0.1766	0.1262
0.25	0.03125	0.001	0.001	0.6498	0.1766	0.1262
0.25	0.03125	0.01	0.001	0.6498	0.1763	0.1267
0.25	0.03125	0.0001	0.01	0.6498	0.1766	0.1262
2	0.03125	0.01	0.1	0.6497	0.1754	0.1314
0.25	0.03125	0.0001	0.1	0.6493	0.1765	0.1267
0.25	0.03125	0.001	0.1	0.6492	0.1765	0.1267
0.25	0.03125	0.01	0.1	0.6487	0.1764	0.1274
0.5	0.015625	0.0001	0.01	0.6474	0.1773	0.1272
0.5	0.015625	0.001	0.001	0.6473	0.1772	0.1272
0.5	0.015625	0.001	0.01	0.6473	0.1772	0.1272
0.5	0.015625	0.0001	0.001	0.6473	0.1773	0.1272
0.5	0.015625	0.01	0.001	0.6471	0.1770	0.1275
0.5	0.015625	0.01	0.01	0.6471	0.1771	0.1275
1	0.015625	0.0001	0.1	0.6463	0.1769	0.1300
0.5	0.015625	0.001	0.1	0.6461	0.1774	0.1278
1	0.015625	0.01	0.1	0.6458	0.1769	0.1307
0.5	0.015625	0.01	0.1	0.6458	0.1772	0.1281
1	0.015625	0.001	0.1	0.6457	0.1770	0.1303
1	0.015625	0.01	0.01	0.6457	0.1770	0.1303
1	0.015625	0.01	0.001	0.6456	0.1770	0.1303
1	0.015625	0.001	0.01	0.6453	0.1773	0.1301
1	0.015625	0.0001	0.01	0.6452	0.1773	0.1301
0.5	0.015625	0.0001	0.1	0.6452	0.1776	0.1280
1	0.015625	0.001	0.001	0.6452	0.1773	0.1301
1	0.015625	0.0001	0.001	0.6451	0.1773	0.1301
2	0.0625	0.0001	0.01	0.6437	0.1778	0.1348
4	0.0625	0.0001	0.01	0.6437	0.1778	0.1348
1	0.0625	0.0001	0.01	0.6437	0.1778	0.1348
1	0.0625	0.0001	0.001	0.6437	0.1778	0.1348
2	0.0625	0.0001	0.001	0.6437	0.1778	0.1348
4	0.0625	0.0001	0.001	0.6437	0.1778	0.1348
1	0.0625	0.001	0.001	0.6436	0.1778	0.1349
2	0.0625	0.001	0.001	0.6436	0.1778	0.1349
4	0.0625	0.001	0.001	0.6436	0.1778	0.1349
1	0.0625	0.001	0.01	0.6436	0.1778	0.1349
2	0.0625	0.001	0.01	0.6435	0.1778	0.1349
4	0.0625	0.001	0.01	0.6435	0.1778	0.1349
0.25	0.015625	0.01	0.01	0.6430	0.1788	0.1272
2	0.0625	0.001	0.1	0.6430	0.1778	0.1354

4	0.0625	0.001	0.1	0.6430	0.1778	0.1354
1	0.0625	0.001	0.1	0.6430	0.1778	0.1354
2	0.0625	0.0001	0.1	0.6430	0.1778	0.1353
4	0.0625	0.0001	0.1	0.6430	0.1778	0.1353
0.25	0.015625	0.01	0.001	0.6430	0.1789	0.1272
0.5	0.0625	0.0001	0.01	0.6429	0.1779	0.1343
0.5	0.0625	0.0001	0.001	0.6429	0.1779	0.1343
1	0.0625	0.0001	0.1	0.6429	0.1778	0.1353
0.5	0.0625	0.001	0.001	0.6429	0.1780	0.1344
0.5	0.0625	0.001	0.01	0.6428	0.1780	0.1345
0.25	0.015625	0.001	0.1	0.6427	0.1792	0.1274
0.25	0.015625	0.0001	0.1	0.6427	0.1792	0.1275
1	0.0625	0.01	0.001	0.6426	0.1783	0.1362
2	0.0625	0.01	0.001	0.6426	0.1783	0.1362
4	0.0625	0.01	0.001	0.6426	0.1783	0.1362
1	0.0625	0.01	0.01	0.6426	0.1783	0.1363
2	0.0625	0.01	0.01	0.6426	0.1783	0.1363
4	0.0625	0.01	0.01	0.6426	0.1783	0.1363
1	0.0625	0.01	0.1	0.6423	0.1782	0.1369
2	0.0625	0.01	0.1	0.6423	0.1782	0.1369
4	0.0625	0.01	0.1	0.6423	0.1782	0.1369
0.25	0.015625	0.001	0.01	0.6422	0.1792	0.1271
0.25	0.015625	0.001	0.001	0.6422	0.1792	0.1271
0.25	0.015625	0.0001	0.01	0.6422	0.1792	0.1271
0.25	0.015625	0.0001	0.001	0.6422	0.1792	0.1270
0.5	0.0625	0.001	0.1	0.6422	0.1779	0.1351
0.5	0.0625	0.0001	0.1	0.6420	0.1779	0.1350
0.25	0.015625	0.01	0.1	0.6420	0.1790	0.1278
0.5	0.0625	0.01	0.01	0.6420	0.1784	0.1358
0.5	0.0625	0.01	0.001	0.6420	0.1784	0.1358
0.5	0.0625	0.01	0.1	0.6416	0.1783	0.1364
2	0.015625	0.01	0.001	0.6390	0.1788	0.1335
2	0.015625	0.0001	0.01	0.6389	0.1790	0.1334
2	0.015625	0.01	0.01	0.6389	0.1788	0.1335
2	0.015625	0.001	0.01	0.6388	0.1790	0.1334
2	0.015625	0.0001	0.1	0.6388	0.1788	0.1334
2	0.015625	0.001	0.001	0.6387	0.1790	0.1335
2	0.015625	0.0001	0.001	0.6386	0.1790	0.1335
2	0.015625	0.001	0.1	0.6386	0.1788	0.1334
2	0.015625	0.01	0.1	0.6378	0.1788	0.1339
4	0.015625	0.0001	0.1	0.6353	0.1796	0.1345
4	0.015625	0.001	0.1	0.6352	0.1796	0.1345

4	0.015625	0.01	0.1	0.6351	0.1794	0.1345
4	0.015625	0.01	0.01	0.6347	0.1798	0.1346
4	0.015625	0.01	0.001	0.6346	0.1798	0.1347
4	0.015625	0.001	0.01	0.6345	0.1800	0.1346
4	0.015625	0.0001	0.01	0.6344	0.1800	0.1346
4	0.015625	0.001	0.001	0.6343	0.1801	0.1347
4	0.015625	0.0001	0.001	0.6343	0.1801	0.1347
0.25	0.0625	0.0001	0.01	0.6331	0.1807	0.1327
0.25	0.0625	0.0001	0.001	0.6331	0.1807	0.1327
0.25	0.0625	0.001	0.001	0.6330	0.1807	0.1328
0.25	0.0625	0.001	0.01	0.6330	0.1807	0.1329
0.25	0.0625	0.01	0.001	0.6326	0.1808	0.1341
0.25	0.0625	0.01	0.01	0.6325	0.1808	0.1341
0.25	0.0625	0.0001	0.1	0.6324	0.1804	0.1333
0.25	0.0625	0.001	0.1	0.6323	0.1805	0.1335
0.25	0.0625	0.01	0.1	0.6315	0.1807	0.1350
1	0.125	0.0001	0.001	0.5308	0.2080	0.1713
2	0.125	0.0001	0.001	0.5308	0.2080	0.1713
4	0.125	0.0001	0.001	0.5308	0.2080	0.1713
1	0.125	0.0001	0.01	0.5308	0.2080	0.1712
2	0.125	0.0001	0.01	0.5308	0.2080	0.1712
4	0.125	0.0001	0.01	0.5308	0.2080	0.1712
1	0.125	0.001	0.001	0.5308	0.2081	0.1714
2	0.125	0.001	0.001	0.5308	0.2081	0.1714
4	0.125	0.001	0.001	0.5308	0.2081	0.1714
1	0.125	0.001	0.01	0.5307	0.2081	0.1714
2	0.125	0.001	0.01	0.5307	0.2081	0.1714
4	0.125	0.001	0.01	0.5307	0.2081	0.1714
1	0.125	0.01	0.001	0.5306	0.2087	0.1727
2	0.125	0.01	0.001	0.5306	0.2087	0.1727
4	0.125	0.01	0.001	0.5306	0.2087	0.1727
1	0.125	0.01	0.01	0.5306	0.2087	0.1727
2	0.125	0.01	0.01	0.5306	0.2087	0.1727
4	0.125	0.01	0.01	0.5306	0.2087	0.1727
1	0.125	0.0001	0.1	0.5295	0.2078	0.1709
2	0.125	0.0001	0.1	0.5295	0.2078	0.1709
4	0.125	0.0001	0.1	0.5295	0.2078	0.1709
1	0.125	0.001	0.1	0.5295	0.2079	0.1711
2	0.125	0.001	0.1	0.5295	0.2079	0.1711
4	0.125	0.001	0.1	0.5295	0.2079	0.1711
1	0.125	0.01	0.1	0.5292	0.2085	0.1725
2	0.125	0.01	0.1	0.5292	0.2085	0.1725

4	0.125	0.01	0.1	0.5292	0.2085	0.1725
0.5	0.125	0.01	0.001	0.5283	0.2089	0.1722
0.5	0.125	0.001	0.001	0.5282	0.2083	0.1708
0.5	0.125	0.0001	0.001	0.5282	0.2083	0.1707
0.5	0.125	0.01	0.01	0.5282	0.2089	0.1722
0.5	0.125	0.001	0.01	0.5282	0.2083	0.1708
0.5	0.125	0.0001	0.01	0.5282	0.2083	0.1707
0.5	0.125	0.001	0.1	0.5275	0.2081	0.1706
0.5	0.125	0.01	0.1	0.5274	0.2087	0.1720
0.5	0.125	0.0001	0.1	0.5274	0.2081	0.1705
0.25	0.125	0.01	0.1	0.5127	0.2123	0.1695
0.25	0.125	0.01	0.001	0.5125	0.2123	0.1692
0.25	0.125	0.01	0.01	0.5125	0.2123	0.1693
0.25	0.125	0.0001	0.1	0.5123	0.2124	0.1680
0.25	0.125	0.001	0.1	0.5122	0.2124	0.1681
0.25	0.125	0.001	0.01	0.5121	0.2124	0.1680
0.25	0.125	0.001	0.001	0.5120	0.2123	0.1679
0.25	0.125	0.0001	0.01	0.5120	0.2124	0.1678
0.25	0.125	0.0001	0.001	0.5120	0.2124	0.1678
